# Identification of permissive amber suppression sites for efficient non-canonical amino acid incorporation in mammalian cells

**DOI:** 10.1093/nar/gkab132

**Published:** 2021-03-03

**Authors:** Michael D Bartoschek, Enes Ugur, Tuan‐Anh Nguyen, Geraldine Rodschinka, Michael Wierer, Kathrin Lang, Sebastian Bultmann

**Affiliations:** Department of Biology II and Center for Molecular Biosystems (BioSysM), Human Biology and BioImaging, Ludwig-Maximilians-Universität München, Munich 81377, Germany; Department of Biology II and Center for Molecular Biosystems (BioSysM), Human Biology and BioImaging, Ludwig-Maximilians-Universität München, Munich 81377, Germany; Department of Proteomics and Signal Transduction, Max Planck Institute of Biochemistry, Martinsried 82152, Germany; Department of Chemistry, Synthetic Biochemistry, Technical University of Munich, Garching 85748, Germany; Department of Biology II and Center for Molecular Biosystems (BioSysM), Human Biology and BioImaging, Ludwig-Maximilians-Universität München, Munich 81377, Germany; Department of Proteomics and Signal Transduction, Max Planck Institute of Biochemistry, Martinsried 82152, Germany; Department of Chemistry, Synthetic Biochemistry, Technical University of Munich, Garching 85748, Germany; Department of Biology II and Center for Molecular Biosystems (BioSysM), Human Biology and BioImaging, Ludwig-Maximilians-Universität München, Munich 81377, Germany

## Abstract

The genetic code of mammalian cells can be expanded to allow the incorporation of non-canonical amino acids (ncAAs) by suppressing in-frame amber stop codons (UAG) with an orthogonal pyrrolysyl-tRNA synthetase (PylRS)*/*tRNA^Pyl^_CUA_ (PylT) pair. However, the feasibility of this approach is substantially hampered by unpredictable variations in incorporation efficiencies at different stop codon positions within target proteins. Here, we apply a proteomics-based approach to quantify ncAA incorporation rates at hundreds of endogenous amber stop codons in mammalian cells. With these data, we compute iPASS (Identification of Permissive Amber Sites for Suppression; available at www.bultmannlab.eu/tools/iPASS), a linear regression model to predict relative ncAA incorporation efficiencies depending on the surrounding sequence context. To verify iPASS, we develop a dual-fluorescence reporter for high-throughput flow-cytometry analysis that reproducibly yields context-specific ncAA incorporation efficiencies. We show that nucleotides up- and downstream of UAG synergistically influence ncAA incorporation efficiency independent of cell line and ncAA identity. Additionally, we demonstrate iPASS-guided optimization of ncAA incorporation rates by synonymous exchange of codons flanking the amber stop codon. This combination of *in silico* analysis followed by validation in living mammalian cells substantially simplifies identification as well as adaptation of sites within a target protein to confer high ncAA incorporation rates.

## Graphical Abstract

**Graphical Abstract:**
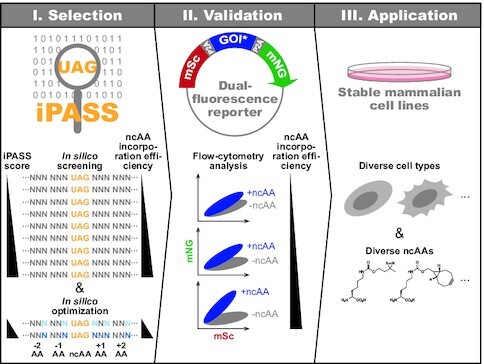
Pipeline to identify or optimize and subsequently validate favorable amber stop codon sequence contexts with high non-canonical amino acid (ncAA) incorporation efficiencies.

## INTRODUCTION

Decoding of in-frame amber stop codons (UAG), generally referred to as amber suppression, enables the translational incorporation of non-canonical amino acids (ncAAs) into target proteins *in vitro* and *in vivo* (1,2). The pyrrolysyl-tRNA synthetase (PylRS, encoded by *PylS*)/tRNA^Pyl^_CUA_ (PylT, encoded by *PylT*) pair from *Methanosarcina* species is one of the most commonly used orthogonal translation systems (OTSs) to incorporate ncAAs at amber stop codons in bacteria (3–5), yeast (6), mammalian cells (7–9) and animals (10–13). This expansion of the genetic code allows site-specific introduction of unique moieties into proteins including bioorthogonal handles for chemical conjugation (14) and photocrosslinkers (15,16) to rationally probe and control protein structure, dynamics, and function in living cells. To date, >100 structurally and functionally diverse ncAAs have been added to the mammalian genetic code (17). The initially low efficiency of ncAA incorporation via amber suppression in mammalian cells has been progressively enhanced by engineering OTS components (18–21) and the eukaryotic release factor 1 (22) as well as by tuning OTS expression levels (22–27) and the generation of stable cell lines (28–30). However, depending on the UAG context, high variations in ncAA incorporation rates are frequently observed in bacteria and mammalian cells (1,2,31–37).

In eukaryotes, the nucleotide frequency around stop codons is non-random. Rather, the nucleotide following the stop codon (+4; stop codon corresponds to nucleotides +1, +2, +3), which together with the stop codon forms a tetranucleotide termination signal, is biased for purines (38–42). This purine bias is especially evident at highly expressed genes and hence has been proposed to promote efficient translational termination (38,42–46). Additionally, stop codon readthrough has been found in prokaryotes, eukaryotes, as well as plant and animal viral RNAs (47). Numerous studies have documented that basal stop codon readthrough by near-cognate tRNAs in eukaryotes is modulated by the flanking sequence context, with a clear influence of the nucleotides downstream of the stop codon (43,48–66). For example, cytosine at +4 (+4 C) is a hallmark of motifs that trigger translational readthrough in higher eukaryotes (43,48,55,58–61,63,64,66).

Previous studies have indicated that basal translational readthrough and the suppression of amber stop codons in mammalian cells are governed by similar (54,67,68) but not identical (63) context-specific effects. In prokaryotes, purines, especially at +4, have been found to boost ncAA incorporation at in-frame amber stop codons (33,34,69). Contradicting these reports, a recent study failed to identify features that reliably predict ncAA incorporation efficiency at amber stop codons in prokaryotes (70). Moreover, the influence of sequence context on the efficiency of translational termination and amber suppression differs between bacteria and mammalian cells (67,68,71). As a result, UAG contexts found to be favorable in prokaryotes cannot reliably be used for the selection of optimal mammal-specific UAG contexts. To date, literature on context effects in mammalian amber suppression is scarce. The few existing studies have been restricted to analysis of just the downstream nucleotide (67,68) or codon (63), at which +4 C was suggested to have a stimulatory effect. In fact, it remains largely unclear to what extent *cis*-acting sequence elements determine amber suppression and ncAA incorporation rates in mammalian cells with an expanded genetic code.

Due to their stringency in quantifying ncAA incorporation rates, dual-fluorescence reporters in combination with flow-cytometry analysis constitute an attractive high-throughput screening platform to analyze amber suppression efficiencies (72). In these systems, expression of two spectrally distinct fluorescent proteins or fluorophore epitopes is coupled via a linker region harboring the amber stop codon. By calculating the ratio between these two fluorescence intensities in the presence and absence of an ncAA, relative amber suppression efficiencies can be robustly quantified (73,74). Currently, dual-fluorescence reporters like *mCherry-TAG-EGFP* in mammalian cells (8) are routinely used to analyze and compare amber suppression efficiencies across different OTSs, ncAAs, and cell lines. However, their applicability for rapid screening of permissive ncAA incorporation sites in target proteins has not yet been explored.

In this study, we sought to streamline the identification of positions permitting high ncAA incorporation efficiencies in mammalian cells with an expanded genetic code. Applying CRISPR/Cas9 or PiggyBac (PB) transposase-mediated genome engineering, we first established mouse embryonic stem cell (mESC) and human embryonic kidney 293T (HEK293T) cell lines stably expressing the orthogonal *Methanosarcina mazei PylS*/*PylT* pair. Using these cell lines we then performed a novel variation of stochastic orthogonal recoding of translation with enrichment (SORT-E) (75) to characterize the entire amber suppressed proteome (amberome) of mammalian cells for the first time. After labeling amber suppressed proteins with a biotin probe and following enrichment by streptavidin pulldown, we used mass spectrometry-based proteomics to systematically assess the efficiency of ncAA incorporation at hundreds of endogenous amber stop codons. With this data, we built a linear regression model of UAG contexts to predict and adjust ncAA incorporation efficiencies *in silico*, which we call iPASS (Identification of Permissive Amber Sites for Suppression; available at www.bultmannlab.eu/tools/iPASS). The resulting iPASS consensus motif suggests that amber suppression efficiency is subject to synergistic context effects mediated by the nucleotides up- and downstream of UAG. To experimentally validate the robustness of iPASS predictions, we developed a dual-fluorescence reporter for the rapid and reproducible quantification of amber suppression efficiencies at individual sequence contexts within a chosen target protein. Using this reporter in flow-cytometry, we analyzed amber suppression at multiple positions within histones H2A and H3, the *de novo* DNA methyltransferase 3B (DNMT3B), as well as at selected synthetic sequence contexts. Our results demonstrate that overall iPASS reliably predicts relative ncAA incorporation efficiencies, which we show to be independent of ncAA as well as cell line identity. Furthermore, we validate iPASS to optimize amber suppression efficiencies at fixed ncAA incorporation sites by silently mutating the two codons following and preceding the amber stop codon. Collectively, iPASS in combination with our dual-fluorescence reporter provides a methodological framework for advancing the applicability of genetic code expansion technologies in mammalian cells.

## MATERIALS AND METHODS

### Cell culture

#### Cell lines

Human embryonic kidney 293T (HEK293T) cells were acquired from the Leibniz Institute – German Collection of Microorganisms and Cell Cultures (DSMZ #ACC635; Braunschweig, GER) and were not further authenticated. J1 mouse embryonic stem cells (mESCs) were a kind gift of En Li and Taiping Chen and were not further authenticated. Cells were cultured under standard conditions (5% CO_2_, 90% humidity, 37°C). Cells were counted after Trypan Blue staining using a Countstar® BioTech Automated Cell Counter system (Alit Life Science). All cell lines regularly tested negative by PCR for Mycoplasma contamination.

HEK293T cells were maintained in Dulbecco's modified Eagle's medium (DMEM; D6429, Sigma-Aldrich) supplemented with 10% fetal bovine serum (FBS; Sigma-Aldrich) and 50 μg/ml gentamycin (47991.01, SERVA Electrophoresis).

J1 mESCs were maintained on 0.2% (w/v) gelatin-coated (G2500, Sigma-Aldrich) dishes in Dulbecco's modified Eagle's medium (DMEM; D6429, Sigma-Aldrich) supplemented with 16% fetal bovine serum (FBS; Sigma-Aldrich), 0.1 mM 2-mercaptoethanol (M3148, Sigma-Aldrich), 2 mM l-glutamine (G7513, Sigma-Aldrich), 1× MEM non-essential amino acids (M7145, Sigma-Aldrich), 100 U/ml penicillin, 100 μg/ml streptomycin (Pen/Strep; P4333, Sigma-Aldrich), homemade recombinant LIF tested for efficient self-renewal maintenance, and 2i (1 μM PD032591 and 3 μM CHIR99021; Axon Medchem).

To maintain expression of the respective transgenes, stable cell lines were continuously cultured under selection pressure using 1 μg/ml puromycin (A1113803, Thermo Fisher Scientific) and/or 1 mg/ml G418 (A2167, AppliChem).

#### Non-canonical amino acids

Three ncAA stock solutions were prepared for use in mammalian cell culture: (i) 100 mM BocK in 100 mM NaOH; (ii) 50 mM DiazK in 100 mM TFA (Trifluoroacetic acid); (iii) 100 mM BcnK in 200 mM NaOH, 15% (v/v) DMSO. All solutions were 0.2 μm sterile filtered and stored at −20°C.

Immediately before adding to cell culture medium, ncAA stock solutions were freshly diluted in 3 volumes of 1 M HEPES (15630056, Thermo Fisher Scientific) to neutralize pH. Within all cell culture experiments, a final concentration of 0.5 mM ncAA was used. For –ncAA control samples, cell culture medium was supplemented with the respective solvent only.

### CRISPR/Cas9 genome engineering

To MIN-tag (*attP* site for Bxb1-mediated recombination; see (76)) the *Gt(ROSA)26Sor* (R26) locus (77,78) in mESCs, sgRNA (R26_sgRNA) targeting R26 exon 1 (NCBI ref. seq. NR_027008.1) was designed using the Benchling CRISPR design online tool (https://benchling.com [Biology Software]; accessed 2015) and cloned into a modified version of the plasmid pSpCas9(BB)-2A-GFP (PX458, a gift from Feng Zhang, Addgene plasmid #48138; (79)), where we fused a truncated form of human Geminin (hGem) to SpCas9 increasing homology-directed repair efficiency (80). A 200 nt ssDNA repair template (R26_toligo; 4 nmole Ultramer™ DNA Oligo, Standard Desalting, Integrated DNA Technologies) was designed with homology arms centered around the MIN-tag. Re-cleavage after repair template incorporation was prevented by co-delivering a CRISPR/Cas9-blocking mutation within the respective sgRNA PAM. To generate the homozygous R26^MIN^ mESC line, 500 000 cells were transfected in a six-well plate with 2.0 μg ssDNA repair template and 0.5 μg SpCas9 plasmid using Lipofectamine3000 (Thermo Fisher Scientific) according to the manufacturer's instructions. 48 h after transfection, GFP positive cells were enriched by fluorescence-activated cell sorting with a BD FACS Aria II (BD FACSDiva Software version 6.1.3, Firmware version 1.6, BD Biosciences). GFP-positive cells were pooled and plated at clonal density into a p100 cell culture dish. After 7 days, single colonies were picked manually using a 10 μl sterile pipette tip, transferred to a 96-well plate (flat bottom), and expanded. To screen for R26^MIN^ clones, 96-well plates were duplicated after cell outgrowth and genomic DNA isolated for screening by PCR and HincII restriction digest as described previously with minor modifications (76). Briefly, cells were washed two times with Dulbecco's PBS (D8537, Sigma-Aldrich), resuspended in 50 μl/well lysis buffer (50 mM TRIS/HCl pH 7.5, 10 mM CaCl_2_, 1.7 μM SDS, 50 μg/ml Proteinase K), frozen at −80°C for 30 min, incubated at 56°C for 3 h, and finally Proteinase K heat inactivated at 85°C for 30 min. 2.5 μl/well of the resulting crude cell lysate were directly subjected to PCR (25 μl/rxn, 0.1 μl MyTaq™ DNA Polymerase, BIO-21107, Bioline) using the external screening primers R26_scr.fwd and R26_scr.rev and following cycling settings: 95°C/5 min – [95°C/30 s – 60°C/30 s – 72°C/30 s] × 45 – 72°C/40 s – 4°C/∞. MIN-tagged clones were identified by restriction fragment analysis of 7.5 μl PCR product using the HincII restriction site located within the MIN-tag (20 μl/rxn, 0.25 μl FastDigest HincII, Thermo Fisher Scientific). R26^MIN^ candidates were further verified by Sanger sequencing (Mix2Seq, Eurofins Genomics) of R26 exon 1 after genomic DNA isolation using the QIAamp DNA Mini Kit (QIAGEN) according to the manufacturer's instructions.

### Generation of stable cell lines

#### Bxb1-mediated recombination

500 000 R26^MIN^ mESCs were transfected in a six-well plate with 1.25 μg of the respective MIN-tag compatible vector harboring an *attB* site and 1.25 μg Bxb1 integrase plasmid pCAG-NLS-HA-Bxb1 (a gift from Pawel Pelczar, Addgene plasmid #51271; (81)) using Lipofectamine3000 (Thermo Fisher Scientific) according to the manufacturer's instructions. After 48 h, mESCs were plated at clonal density in a p100 cell culture dish and 1 mg/ml G418 (A2167, AppliChem) was added. After 7 days, single colonies were picked manually using a 10 µl sterile pipette tip, transferred to a 96-well plate, and expanded. To screen for Bxb1 recombined clones, 96-well plates were duplicated after cell outgrowth and genomic DNA isolated for screening by PCR as described previously with minor modifications (76). Briefly, cells were washed two times with Dulbecco's PBS (D8537, Sigma-Aldrich), resuspended in 50 μl/well lysis buffer (50 mM TRIS/HCl pH 7.5, 10 mM CaCl_2_, 1.7 μM SDS, 50 μg/ml Proteinase K), frozen at −80°C for 30 min, incubated at 56°C for 3 h, and finally Proteinase K heat inactivated at 85°C for 30 min. 2.5 μl/well of the resulting crude cell lysate were directly subjected to PCR (25 μl/rxn, 0.1 μl MyTaq™ Red DNA Polymerase, BIO-21110, Bioline) using the external screening primers R26_scr.fwd and R26_scr.rev in combination with attL_scr.fwd and following cycling settings: 95°C/5 min – [95°C/30 s – 60°C/30 s – 72°C/30 s] × 45 – 72°C/40 s – 4°C/∞. Bxb1 recombined clones stably harboring the respective synthetase (R26^RS^) are identified by the attL_scr.fwd and R26_scr.rev PCR product. To validate stable R26^RS^ clones, genomic DNA was isolated using the QIAamp DNA Mini Kit (QIAGEN) according to the manufacturer's instructions and the PCR repeated on 20 ng purified genomic DNA.

#### PiggyBac transposition

500 000 HEK293T or mESCs were transfected in a six-well plate with 1.875 μg of the respective donor plasmid and 0.625 μg PiggyBac transposase vector (System Biosciences, #PB200PA-1) using Lipofectamine3000 (Thermo Fisher Scientific) according to the manufacturer's instructions. After 48 h, cells were plated at 40% confluency in a p100 cell culture dish and the respective selection antibiotic, 1 μg/ml puromycin (A1113803, Thermo Fisher Scientific) or 1 mg/ml G418 (A2167, AppliChem), was added. Cells were passaged at least two times under selection pressure to generate stable polyclonal pools before commencing experiments. PiggyBac transposition was used to establish HEK293T cells stably expressing the respective synthetase (HEK293T^RS^) and R26^RS^ mESC clones stably expressing the mSc/mNG fluorescent reporter harboring an amber mutated GOI* coding sequence or context* (R26^RS^/PB^GOI*^ or R26^RS^/PB^context*^).

### Transient transfections

300 000 or 40 000 stable HEK293T^RS^ cells per 12- or 96-well, respectively, were seeded into ncAA containing medium 4 h before transfection. 250 000 stable R26^RS^ mESCs were plated per 12-well 2 h before transfection and ncAA was added at transfection. Cells were transfected with 1.0 μg (12-well) or 225 ng (96-well) of the respective plasmid using Lipofectamine3000 (Thermo Fisher Scientific) according to the manufacturer's instructions and incubated for 24 h before flow-cytometry.

### Flow-cytometry data collection and analysis

Transiently transfected R26^RS^ mESCs and HEK293T^RS^ cells were analyzed 24 h after transfection. Stable R26^RS^/PB^GOI*^ or R26^RS^/PB^context*^ mESCs were seeded at 30% confluency into ncAA containing medium in 12- or 96-wells and analyzed after 24 h.

For flow-cytometry, cells grown in 12- or 96-wells were washed with 1 ml or 200 μl Dulbecco's PBS (D8537, Sigma-Aldrich), dissociated with 100 μl or 28 μl Trypsin-EDTA in PBS (T4299, Sigma-Aldrich), and resuspended in 500 or 100 μl FluoroBrite Dulbecco's modified Eagle's medium (DMEM; A1896701, Thermo Fisher Scientific) supplemented with 10% fetal bovine serum (FBS; Sigma-Aldrich) and 100 U/ml penicillin, 100 μg/ml streptomycin (Pen/Strep; P4333, Sigma-Aldrich). Before acquisition, cells from 12-wells were filtered through a 35 μm cell strainer (352235, Corning) and cells in 96-well plates were thoroughly resuspended using a multichannel pipette. Cells were recorded on a BD LSRFortessa (BD FACSDiva Software version 8.0.1, Firmware version 1.4, BD Biosciences) with a BD High Throughput Sampler (HTS, BD Biosciences) for loading of 96-well plates.

Flow-cytometry data of the mSc/mNG dual-fluorescence reporter were processed in three steps with FlowJo (version 10.6.1, BD Biosciences) by (i) gating for single cells excluding debris (FSC-A/SSC-A) and doublets (FSC-A/FSC-H and SSC-A/SSC-H), (ii) gating for transfected/stable cells by excluding mSc negative cells, and (iii) calculating mSc and mNG mean fluorescence intensities (MFIs, see Supplementary Figure S7 and S8 for representative flow-cytometry data). MFIs were further analyzed with RStudio (version1.3.1093, R version 3.6.1; RStudio: Integrated Development Environment for R; RStudio, PBC, Boston, MA; http://www.rstudio.com) using the *tidyverse* (version 1.3.0) (82) and *rstatix* (version 0.6.0; https://rpkgs.datanovia.com/rstatix) R packages. Relative readthrough efficiency (RRE) for samples + or – ncAA and incorporation efficiencies for each position were calculated according to equations from Figure 3B. Flow-cytometry raw data and analysis files are available via FlowRepository (83) with the repository identifier FR-FCM-Z2N3.

### Purification of amber suppressed endogenous proteins by streptavidin pulldown (SORT-E)

#### Proteomic incorporation of BcnK at amber stop codons

1.6 × 10^6^ R26^MIN^ and R26^RS_BcnK^ mESCs or wtHEK293T and HEK293T^RS_BcnK^ cells were seeded per p150 plate and after 2 h 0.5 mM BcnK diluted in 3 volumes of 1 M HEPES (15630056, Thermo Fisher Scientific) was added. After 66–72 h, cells on p150 plates were washed once with Dulbecco's PBS (D8537, Sigma-Aldrich), incubated for 1 h with fresh medium, washed a second time with PBS, and incubated for another 3 h with fresh medium. For harvesting, cells on p150 plates were washed once with PBS, dissociated with 2 ml trypsin–EDTA solution (T3924, Sigma-Aldrich), resuspended in 10 ml fresh medium, and collected by centrifugation at 500 g and 4°C for 5 min. Cell pellets were washed on ice two times by resuspending in 10 ml ice cold PBS and centrifugation at 500 g and 4°C for 5 min. Pellets were flash frozen in liquid N_2_ and stored at −80°C.

#### Full proteome samples

For each sample, 10% of flash-frozen cell pellet from one p150 plate (ca. 2.5 × 10^6^ cells) were lysed in 200 μl lysis buffer (6 M guanidinium Chloride, 100 mM Tris–HCl pH 8.5, 2 mM DTT). Samples were homogenized by pipetting and boiled at 99°C for 10 min with constant shaking at 1400 rpm. After quickly spinning down, samples were sonicated at 4°C for 15 min in 1.5 ml tubes using a Bioruptor^®^ Plus sonication device (Diagenode) with the following settings: high intensity, 30 s on/30 s off cycle. Protein concentrations were then determined using the Pierce™ BCA Protein Assay Kit (23225, Thermo Fisher Scientific) according to the manufacturer's instructions for microplate settings. Meanwhile, samples were alkylated with 40 mM chloroacetamide (CAA) for 20 min at room temperature. Afterwards, 30 μg of lysate was diluted in a total volume of 50 μl lysis buffer supplemented with 40 mM CAA and 2 mM dithiothreitol (DTT). Samples were then diluted 1:10 with digestion buffer (10% acetonitrile, 25 mM Tris–HCl pH 8.5). To each sample 0.6 μg Trypsin (Pierce™ Trypsin Protease, 90058, Thermo Fisher Scientific) and 0.6 μg LysC (Pierce™ LysC Protease, 90051, Thermo Fisher Scientific) was added and proteins were digested overnight at 37°C with constant shaking at 1100 rpm. The next day, protease digestion was stopped by adding 1% (v/v) trifluoroacetic acid (TFA) and samples were loaded on StageTips containing three layers of SDB-RPS matrix (Empore) in a 200 μl pipette tip according to standard protocol (84). After one washing step with 0.1% (v/v) TFA, peptides were eluted into 60 μl of 80% acetonitrile and 2% ammonium hydroxide. Evaporation of the eluates was performed in a SpeedVac centrifuge and peptides were subsequently resuspended in 20 μl of A* buffer (0.1% TFA, 2% acetonitrile) and shook for 10 min at 2000 rpm at room temperature prior to peptide concentration estimations at 280 nm.

#### In vitro chemoselective labeling of BcnK tagged proteomes with biotin-tetrazine conjugate

For each sample, 90% of flash frozen cell pellet from one p150 plate (ca. 22.5 × 10^6^ cells) were lysed on ice with 1 volume of ice cold RIPA buffer (50 mM Tris–HCl pH 8.0, 150 mM NaCl, 0.1% UltraPure™ SDS Solution (24730020, Invitrogen), 0.5% sodium deoxycholate detergent, 1% Triton X-100; freshly add 1× cOmplete™ EDTA-free Protease Inhibitor Cocktail (04693132001, Roche)) and sonicated at 4°C for 20 min in 1.5 ml tubes using a Bioruptor^®^ Plus sonication device (Diagenode) with the following settings: high intensity, 30 s on/30 s off cycle. Lysates were subsequently cleared by centrifugation at 20 000 g and 4°C for 15 min and supernatants collected. Protein concentrations were then determined using the Pierce™ BCA Protein Assay Kit (23225, Thermo Fisher Scientific) according to the manufacturer's instructions for microplate settings. Cleared cell lysates were diluted with RIPA buffer to a final concentration of 3 mg/ml protein and 1 ml lysate was typically used. Therefore, 1 ml lysates (3 mg protein input) were reduced with 2 mM DTT for 30 min on ice and subsequently alkylated with 40 mM chloroacetamide (CAA) for 45 min on ice. 7.5 μM biotin-tetrazine conjugate (2.5 nmol biotin-tetrazine/1 mg protein input) was then added to lysates and incubated overnight at 4°C with end-over-end rotation. The next day, 50 μl aliquots (150 μg protein) were boiled for 10 min at 95°C in 1× Laemmli buffer supplemented with 20 mM DTT as input samples for analysis by western blot.

#### Streptavidin pulldown

60 μl settled resin per sample (binding capacity: 160 μg biotinylated BSA/1 mg protein input) of Pierce™ High Capacity NeutrAvidin™ Agarose (29202, Thermo Fisher Scientific) were washed three times in 3 volumes of RIPA buffer, diluted in RIPA buffer to 120 μl slurry per sample, and added to biotin-tetrazine labeled lysates. Samples were then incubated for 2 h at room temperature with end-over-end rotation. After 2 h, 50 μl aliquots of supernatants were boiled for 10 min at 95°C in 1× Laemmli buffer supplemented with 20 mM DTT as unbound fraction for analysis by western blot. The remaining supernatant was aspirated and agarose beads were washed on ice by resuspending in 1 ml of the following buffers and centrifugation for 3 min at 500 g and 4°C: two times in RIPA buffer, once in 1 M KCl, once in 100 mM Na_2_CO_3_, and twice in urea buffer (2 M urea solution (U4883, Sigma-Aldrich), 50 mM ammonium bicarbonate). During the last washing step, agarose beads were transferred to a fresh 1.5 ml tube. For western blot analysis, 10% of agarose beads were washed two more times in RIPA buffer and proteins eluted by boiling for 10 min at 95°C in 1× Laemmli supplemented with 20 mM DTT and 2 mM biotin. For mass spectrometry analysis, peptides were eluted from beads by resuspending in 200 μl elution buffer (1 M urea solution (U4883, Sigma-Aldrich), 50 mM ammonium bicarbonate) and on-beads-digest with 1.5 μg Pierce™ trypsin protease (0.5 μg trypsin/1 mg protein input; 90058, Thermo Fisher Scientific) for 18–20 h shaking at 30°C and 1300 rpm. Trypsinization was stopped by adding 1% (v/v) trifluoroacetic acid (TFA) and samples were stored at −20°C. Eluted peptides were desalted and concentrated using C18 based StageTips according to standard protocol (84). Evaporation of the eluates was performed in a SpeedVac centrifuge and peptides were subsequently resuspended in 20 μl of A* buffer (0.1% TFA, 2% acetonitrile) and shook for 10 min at 2000 rpm and room temperature prior to peptide concentration estimations at 280 nm.

### LC–MS/MS

#### Acquisition of full proteomes and SORT-E eluates

Each sample was loaded on a 50 cm C18-based reversed phase column (in-house packed with ReproSil-Pur C18-AQ 1.9 μm resin from Dr Maisch a total inner diameter of 75 μm), which was mounted on an EASY-nLC 1200 (Thermo Fisher Scientific) ultra-high pressure system and constantly kept at 60°C. The liquid chromatography was coupled to a Q Exactive HF-X Hybrid Quadrupole-Orbitrap Mass Spectrometer (Thermo Fisher Scientific) via a nano-electrospray source and operational parameters were monitored by SprayQc. Peptides were eluted constantly at around 300 nl/min during a 120 min non-linear ACN gradient. After each set of replicates (R26^MIN^ + wtHEK293T in triplicates and R26^RS_BcnK^ + HEK293T^RS_BcnK^ in quadruplicates) an additional wash step was scheduled. Data-dependent acquisition was applied; after sequential full scans (maximum injection time: 20 ms, resolution: 60 000, target value 3 × 10^6^) the most abundant 12 ions were addressed to MS/MS scans. The *m*/*z* range was limited to 400–1650 *m*/*z*.

The mass spectrometry proteomics data have been deposited to the ProteomeXchange Consortium via the PRIDE (85) partner repository with the dataset identifier PXD019815.

#### Computational analysis of raw MS data of full proteomes and SORT-E samples

Analysis of raw MS data was accomplished by the MaxQuant software package (version 1.6.11.0 (86)). The underlying FASTA files for peak list searches were derived from Uniprot by including both reviewed and unreviewed proteomes (mouse proteome, version October 2018; human proteome, version May 2020). An additional common contaminant list comprising 262 entries was applied using the Andromeda search engine (87). The ‘Match between runs’ option was enabled and the FDR was set to 1%, which applies on protein and peptide level (minimum of seven amino acids). Relative quantification of proteins was accomplished by the MaxLFQ algorithm (88). The cut-off was set to a minimal ratio count of two peptides.

For both full proteome and SORT-E samples the initial MaxQuant output was analyzed by Perseus (version 1.6.2.3). Here, common contaminants and protein groups measured less than twice within at least one set of replicates were filtered out and LFQ values were transformed into log_2_-values.

#### Statistical analysis of full proteomes

For full proteomes, imputation of missing values was based on a gaussian distribution relative to the standard deviations of measured values (width of 0.2 and a downshift of 1.8 standard deviations). Student's *t*-tests of R26^RS_BcnK^ versus R26^MIN^ and HEK293T^RS_BcnK^ versus wtHEK293T were performed with a permutation-based FDR of 0.05 and a minimal log_2_ fold change of 1 (S0).

GO analysis of differentially expressed proteins according to both Student's *t*-tests was performed by using the Panther classification system (89). Here, the up- and downregulated proteins were analyzed together due to the low amount of significantly changing proteins. GO terms with a lower fold change of 2 or a higher *P*-value than 0.05 were excluded.

#### Preprocessing of proteomics data for iPASS linear regression model

SORTE-E samples were normalized for differences in protein expression levels by subtracting the full proteome LFC (expression level) from their matched SORT-E LFC (enrichment in pulldown). The resulting normalized replicates of control (R26^MIN^; wtHEK293T) and amber suppressed samples (R26^RS_BcnK^; HEK293T^RS_BcnK^) were tested for significant difference using a two-sided Student's *t*-test. Proteins with a *P*-value <0.01 were considered significantly enriched. Stop codon identity, sequence context, and GC content around the stop codon (positions −6 to +9) were extracted from coding sequences and cDNA assemblies of *Mus musculus* (GRCm38) and *Homo sapiens* (GRCh38) using custom Python scripts and assigned to the respective proteins. Isoforms of individual proteins were filtered by keeping only unique sequence contexts.

### Linear regression model (iPASS model)

#### Linear regression analysis

Regression analysis was performed as described previously (61). In brief, to predict amber suppression efficiencies for a given UAG context we employed a linear regression model based on the sequence context (SC), the GC content, and their normalized fold changes obtained by SORT-E (see above). The SC included the stop codon itself (positions +1 to +3) and the nucleotide sequences surrounding the stop codon (positions −6 to -1, +4 to +9). Nucleotide sequences were represented by indicator vector coding. Here, 12 × 4 binary vector entries are used to indicate the presence [1] or absence [0] of a nucleotide (A, C, G, or U) at a particular position (−6 to −1, +4 to +9) surrounding the stop codon. Three further entries are reserved to indicate the type of stop codon (UAA, UAG, or UGA; positions +1 to +3) and a separate column for the GC content of the sequence from positions −6 to +9. The resulting feature vectors of all sequences were scaled using the *preProcess* function of the (v6.0–78) R package (90). Regularized ridge regression was performed using the *glmnet* (v2.0–13) R package.

For the estimation of the regression model coefficients, we performed a regularized least-squares (‘ridge’) regression (91). Let **X** be the *n* × *d* matrix of *n* sequence feature vectors with dimensionality *d* and **y** be the (*n*-dimensional) vector of readthrough values associated with the sequences. Then the weight vector **w** = (**X**T**X** + *k* × **I**)−1 × **X**T**y** represents the solution of the linear least-squares problem and *y* = **w**T**x** corresponds to the RTP value *y* for a sequence feature vector **x**. The minimum loo-cv error (lambda) in terms of the sum of squared deviations of predictions from known readthrough values was 0.13 for *k* = 10^0.3^ (∼1.995).

The decoy model was created as described above keeping identical proteins and SCs but randomly reshuffling LFC values.

#### Feature elimination analysis

Starting from the complete iPASS model, we removed the variable corresponding to the minimum sum of squared regression coefficients. The residual error was then calculated for the remaining variables (including the stop codon) as described above. This procedure was repeated until only the stop codon variable was left.

#### Probability logo construction

To construct a probability logo (motif) reflecting sequence contexts for efficient amber suppression, we first generated all possible 12-mer sequence contexts (4^12^) comprised of the nucleotides 6 bp up- and downstream of a central amber stop codon (nucleotides –6 to –1 and +4 to +9; stop codon at +1, +2, +3) *in silico*. After removal of sequences containing in-frame stop codons, we used the iPASS model to predict amber suppression efficiencies of all 13 845 841 *k*-mers. To construct a probability logo, this list of *k*-mers together with their iPASS scores was used as the input for kpLogo (v1.1) (92) with the options *-k 1 -weighted*.

### Supplementary material and methods

Additional material and methods including plasmid construction, chemicals and chemical synthesis, and western blotting are available as supplementary material at NAR online. NMR spectra of synthesized chemicals are depicted in Supplementary Figure S13. Uncropped SDS-gel and blots are presented in Supplementary Figure S14 and S15. Plasmids used and cloned and oligonucleotides used in this study are listed in Supplementary Table S1 and S2 within Supplementary Material and Methods. Plasmids cloned in this study have been deposited at Addgene with the IDs 167491–99.

## RESULTS

### Step-wise generation of stable mESC and HEK293T lines with an expanded genetic code

**Figure 1.**
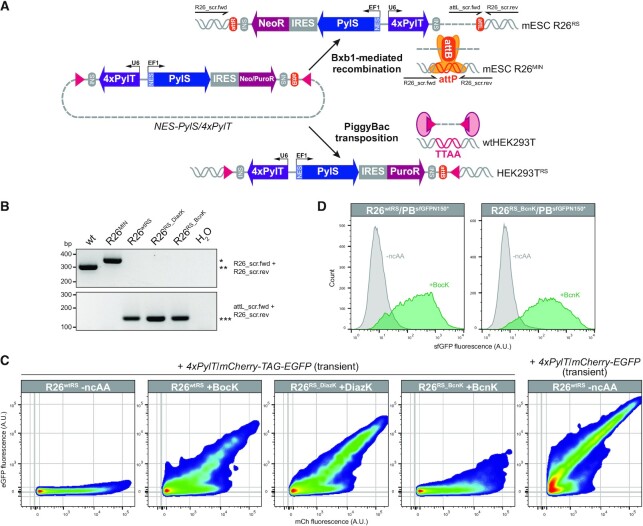
(**A**) Strategy to generate stable cell lines with an expanded genetic code. Bxb1-mediated recombination in mouse embryonic stem cells (mESCs) homozygous (only one allele is depicted) for the MIN-tag within the *Rosa26* locus (R26^MIN^) or PiggyBac (PB) transposition in wild-type human embryonic kidney cells (wtHEK293T). The Bxb1 integrase specifically recombines the attachment sites *attP* and *attB* to generate *attR* and *attL* sites that flank the integrated vector, whereas the PB transposase integrates the cassette that is flanked by inverted terminal repeats (ITRs, indicated as rectangles) into TTAA chromosomal sites. Primer binding sites used for screening of stable R26^RS^ clones are indicated. Abbreviations: *Methanosarcina mazei* tRNA^Pyl^ synthetase (PylS) N-terminally fused to a nuclear export signal (NES), tRNA^Pyl^_CUA_ (PylT), internal ribosomal entry site (IRES), neomycin resistance (NeoR), puromycin resistance (PuroR), constitutive EF1α promoter (EF1), constitutive U6 promoter (U6), insulator (INS). (**B**) Sequential stable integration of the MIN-tag and *NES-PylS/4xPylT* into the *Rosa26* locus (R26) in mESCs. Agarose gel electrophoresis of screening PCRs using the indicated primers. Homozygous integration of the MIN-tag (*attP* site) into R26 results in a 48 bp shift (*) compared to wt (**). Subsequent Bxb1-mediated stable integration of *NES-wtPylS*/*4xPylT* (R26^wtRS^), *NES-PylS_DiazK*/*4xPylT* (R26^RS_DiazK^), or *NES-PylS_BcnK*/*4xPylT* (R26^RS_BcnK^) generates an *attL* binding site (***). (**C**) Variable amber suppression efficiencies in stable mESC lines expressing PylRS variants. R26^wtRS^, R26^RS_DiazK^, or R26^RS_BcnK^ mESCs transiently transfected with the *4xPylT*/*mCherry-TAG-EGFP* reporter construct were cultured for 24 h in the presence of the indicated ncAA (0.5 mM final concentration). The *4xPylT*/*mCherry-EGFP* reporter construct lacking the amber stop codon was transiently transfected as reference of the optimal mCherry/EGFP ratio. For flow-cytometry measurements, 9000 mCh positive single cells per condition were analyzed (for gating strategy and complete panel of dot plots see Supplementary Figure S2). Fluorescence intensities are indicated in arbitrary units (A.U.). (**D**) Efficient incorporation of ncAAs into target proteins in mESCs after stable integration of both *NES-PylS*/*4xPylT* and amber transgene. The *4xPylT*/sfGFP^N150*^ reporter construct harboring the amber stop codon within the *sfGFP* ORF was integrated in R26^wtRS^ or R26^RS_BcnK^ mESCs by PB transposition (R26^wtRS^/PB^sfGFPN150*^ and R26^RS_BcnK^/PB^sfGFPN150*^). Incorporation of the respective ncAA (0.5 mM final concentration) into sfGFP^N150*^ was verified after 48 h by flow-cytometry analysis of 20 000 single cells per condition. Fluorescence intensity of sfGFP is indicated in arbitrary units (A.U.).

Efficient amber suppression in mammalian cells has been reported to depend on high suppressor tRNA expression levels (22,23,25,27), whereas two *PylS* copies are sufficient to expand the genetic code of mice (13). We therefore speculated that efficient amber suppression in mESCs could be achieved by biallelic integration of a construct harboring one copy of *PylS* and four copies of *PylT* into the *Gt(ROSA)26Sor* (R26) genomic safe harbor locus (77,78). To this end, we applied our previously developed multifunctional integrase (MIN) tag genome engineering strategy (76), which leverages Bxb1-mediated recombination between *attB* and *attP* attachment sites (Figure 1A). In a first step, we established a monoclonal mESC line harboring the *attP* site (MIN-tag) within R26 (R26^MIN^) using CRISPR/Cas9 gene editing. Homozygous integration of the MIN-tag into the R26 locus was confirmed by agarose gel electrophoresis and Sanger sequencing (Supplementary Figure S1A+B). In a second step, the MIN-tagged R26 serves as a genetic entry site for the rapid and selective integration of *PylS*/*PylT* pairs. To construct a targeting vector compatible with PB- as well as Bxb1-mediated genomic integration, we modified a previously reported 4x*PylT*/*PylS* vector (30) to include an *attB* attachment site (Figure 1A). The encoded wtPylRS transfers the pyrrolysine analog tert-butoxycarbonyl-l-lysine (BocK, Supplementary Figure S1C) onto PylT in mammalian cells (7). To expand the toolkit of ncAAs that can be incorporated, we also generated targeting vectors encoding two additional *PylS* variants: (i) *PylS_DiazK* to incorporate the ncAA methyl-diazirin-l-lysine (DiazK, Supplementary Figure S1C+D) bearing a diazirine moiety for site-directed photocrosslinking of proteins (6,93–95); (ii) *PylS_BcnK* to incorporate the ncAA bicyclo[6.1.0]nonyne-l-lysine (BcnK, Supplementary Figure S1C) bearing a strained alkyne motif for selective labeling with tetrazine conjugates via inverse electron-demand Diels–Alder cycloaddition (iEDDAC) (96–99). Additionally, we N-terminally tagged wild-type (wt) as well as engineered PylRS variants with a nuclear export signal (NES), which has been reported to enhance amber suppression efficiency up to 15-fold (19). After co-transfecting the targeting vector and a Bxb1 expression plasmid, we selected for stable *NES-PylS*/4x*PylT* integrants using the co-delivered neomycin resistance cassette. We verified that the three *NES-PylS*/4x*PylT* targeting vectors had been inserted into both R26 alleles (R26^RS^, Figure 1B) in each of the newly generated cell lines: (i) *NES-wtPylS*/4x*PylT* (clone: R26^wtRS^); (ii) *NES-PylS_DiazK*/4x*PylT* (clone: R26^RS_DiazK^); and (iii) *NES-PylS_BcnK*/4x*PylT* (clone: R26^RS_BcnK^). In addition, we generated three polyclonal HEK293T lines by PB-mediated stable integration (30) of *NES-PylS*/4x*PylT* cassettes and puromycin selection (HEK293T^RS^, Figure 1A): (i) *NES-wtPylS*/4x*PylT* (cell line: HEK293T^wtRS^); (ii) *NES-PylS_DiazK*/4x*PylT* (cell line: HEK293T^RS_DiazK^); and (iii) *NES-PylS_BcnK*/4x*PylT* (cell line: HEK293T^RS_BcnK^).

To validate amber suppression in stable R26^RS^ cell lines, we transiently transfected a *4xPylT*/*mCherry-TAG-EGFP* reporter construct (30), which expresses full-length mCh-eGFP upon efficient decoding of the amber stop codon. After 24 h in the presence or absence of the respective ncAA, we analyzed mCh and eGFP fluorescence by flow-cytometry (Figure 1C, Supplementary Figure S2). In comparison to the incorporation of BocK in R26^wtRS^ and DiazK in R26^RS_DiazK^, BcnK was less efficiently incorporated in R26^RS_BcnK^, indicated by the lower correlation between mCh and eGFP fluorescence. This difference may be attributable to the reduced PylRS aminoacylation activity of BcnK compared to BocK or DiazK (99). Furthermore, the transfection efficiency of stable R26^RS^ clones was generally low (∼25%, Supplementary Figure S2B). Genomic integration of *PylS/PylT* via the MIN-tag strategy allowed us to use PB transposition in a second step to establish polyclonal pools stably co-expressing multiple copies of the gene of interest harboring an in-frame amber stop codon (GOI*; cell line: R26^RS^/PB^GOI*^). As a proof of principle, we stably integrated a *4xPylT*/sfGFP^N150*^ reporter construct, which harbors the amber stop codon at position 150 of *sfGFP* (30), into R26^wtRS^ and R26^RS_BcnK^ cells. After selection with puromycin, we analyzed expression of full-length sfGFP in the absence or presence of BocK or BcnK by flow-cytometry (Figure 1D). Both R26^RS^/PB^sfGFPN150*^ stable cell lines suppressed the amber stop codon within sfGFP^N150*^ upon induction with the respective ncAA. This demonstrates that two *PylS* copies expressed from the R26 genomic locus of mESCs are sufficient to direct efficient amber suppression. In summary, we established stable and defined mESC clones capable of amber suppression and compatible with PB transposition to genomically integrate *4xPylT*/GOI* expression cassettes.

### A linear regression model of amber stop codon contexts to predict ncAA incorporation efficiencies

Next, we wondered to what extent the nucleotide composition around UAG determines ncAA incorporation efficiencies. Genetic code expansion with *PylS*/*PylT* in HEK293T cells has been reported to suppress endogenous amber stop codons resulting in off-target labeling of the cellular proteome (25,99). In line with these studies, we observed widespread amber suppression of endogenous proteins in the stable R26^RS_BcnK^ mESC clone by in-gel fluorescence analysis of BcnK harboring proteins with a silicon rhodamine-tetrazine conjugate (SiR-Tet) (Supplementary Figure S1E, S3A). We hypothesized that assessment of ncAA incorporation rates in living cells at endogenous UAG contexts would allow us to identify sequence motifs that stimulate amber suppression in a target GOI*. To this end, we leveraged the chemoselective iEDDAC reaction between BcnK and tetrazine-based probes (97,98) to implement a novel adaptation of the SORT-E strategy by Elliott *et al.* (75). Here, we tagged BcnK harboring endogenous proteins with a biotin–tetrazine (Biotin-Tet) probe (Supplementary Figure S1E), which enables amber suppressed proteins to be selectively enriched by streptavidin pulldown and subsequently identified by mass spectrometry (75). Since pulldown by streptavidin initially depends on ncAA incorporation and hence amber suppression, endogenous proteins with UAG contexts permitting high BcnK incorporation rates should be enriched and can be subsequently extracted for bioinformatic analysis.

**Figure 2.**
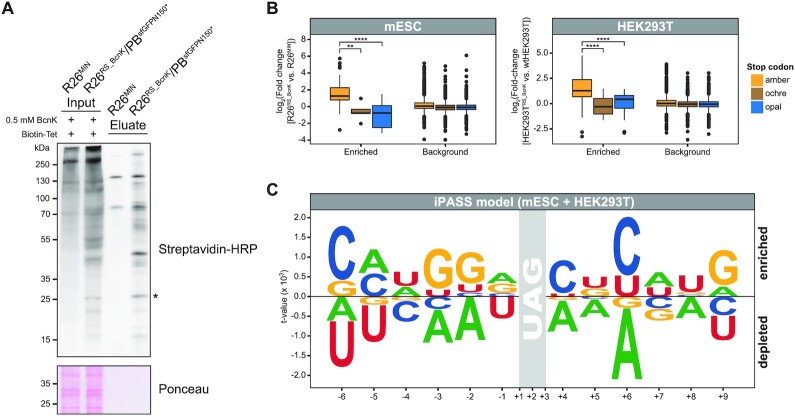
(**A**) Selective streptavidin pulldown of amber suppressed endogenous proteins in stable R26^RS_BcnK^ mESCs after covalent labeling with a biotin-tetrazine (Biotin-Tet) probe (SORT-E approach). Stable R26^RS_BcnK^/PB^sfGFPN150*^ and R26^MIN^ mESCs were cultured for 68 h in the presence of 0.5 mM BcnK, whole cell lysates labeled with Biotin-Tet, and biotinylated proteins captured by streptavidin pulldown. Input and eluate samples were subjected to western blotting using a streptavidin-HRP conjugate and Ponceau S staining as loading control. Amber suppressed sfGFP^N150*^ (*) is indicated. (B, C) After SORT-E and analysis of pulldowns by LC–MS/MS (Supplementary Figure S3C), significantly enriched proteins were identified and a linear regression model of enriched stop codon sequence contexts calculated (Supplementary Figure S5A). (**B**) Selective enrichment of amber suppressed endogenous proteins by SORT-E from R26^RS_BcnK^ mESCs and HEK293T^RS_BcnK^ cells cultured with BcnK. The fold change (PylRS_BcnK versus respective control cell line) of proteins harboring one of the three stop codons (amber, ochre, opal) is depicted for the enriched (*P* < 0.01) and background (*P* > 0.01) fraction in HEK293T and mESCs. PylRS_BcnK and control cell lines were analyzed by LC–MS/MS in biological quadruplicates and triplicates, respectively. ANOVA + Tukey's honestly significant difference post-hoc test: **P < 0.01, ****P < 0.0001. (**C**) Probability logo of 12-mer sequence contexts comprised of the nucleotides 6 bp up- and downstream of the amber stop codon (nucleotides –6 to –1 and +4 to +9; stop codon at +1, +2, +3). Each UAG sequence context (4^12^ sequences) was weighted by its computed iPASS score. Character height corresponds to the *t*-value (two-tailed unpaired two-sample Student's *t*-test) with positive and negative values indicating enriched and depleted sequence contexts, respectively. The iPASS model combines the linear regression analysis of significantly enriched stop codon sequence contexts after SORT-E from both mESCs and HEK293T cells.

We first verified labeling of whole cell lysates with Biotin-Tet by western blot after BcnK incorporation in R26^RS_BcnK^ mESCs (Supplementary Figure S3B). Furthermore, we observed a marked enrichment of Biotin-Tet-labeled endogenous proteins after streptavidin pulldown from R26^RS_BcnK^ lysates compared to those from the R26^MIN^ entry cell line lacking *PylS*/*PylT* (Figure 2A). To specifically evaluate ncAA incorporation rates at endogenous UAG contexts, we cultured R26^MIN^ and R26^RS_BcnK^ mESCs or wtHEK293T and HEK293T^RS_BcnK^ cells in the presence of BcnK for 3 days and, after labeling of cellular lysates with Biotin-Tet, performed streptavidin pulldowns (Supplementary Figure S3C–E). To control for protein expression levels, whole cell lysates were collected in parallel for subsequent full proteome measurements from the same samples (Supplementary Figure S3C). Eluates and full proteomes from PylRS_BcnK and respective control cell lines were then analyzed by LC–MS/MS. Principal component analyses (PCA) of LC–MS/MS data revealed that the global proteome was negligibly altered in response to amber suppression with BcnK (Supplementary Figure S4A), with only 61 and 51 proteins exhibiting altered expression in R26^RS_BcnK^ and HEK293T^RS_BcnK^ cells, respectively (Supplementary Figure S4B). Additionally, GO analysis suggested no significant translational perturbance or clear enrichment of a specific pathway, arguing against a directed cellular response to amber suppression with BcnK (Supplementary Figure S4C). In contrast to the full proteome, streptavidin pulldowns from R26^RS_BcnK^ and HEK293T^RS_BcnK^ cells clustered apart from their respective control cell lines in PCA, indicating enrichment of distinct proteins (Supplementary Figure S4A). To identify amber suppressed proteins, we first normalized the protein abundance measured in each pulldown to that measured in each corresponding full proteome, and then determined enrichment by calculating the mean fold changes in protein levels between PylRS_BcnK and respective control cell lines (Supplementary Figure S5A). Importantly, normalization of pulldowns to full proteomes enables the extent of amber suppression for each detected protein to be determined irrespective of its abundance. Using these parameters, we identified 123 and 101 proteins that were significantly enriched (*P* < 0.01) in mESC R26^RS_BcnK^ and HEK293T^RS_BcnK^ pulldowns, respectively (Supplementary Figure S5A, Supplementary Data 1). The majority of all significantly enriched proteins (69%) are terminated by UAG in contrast to the low proportion (27%) in the background fraction (*P* > 0.01) (Supplementary Figure S5A, Supplementary Data 1). This enrichment compares favorably to the theoretical proportion (23%) of proteins terminating at UAG in mammals (41,100), validating the specificity of the streptavidin pulldown for amber suppressed proteins. Furthermore, fold changes of these proteins were significantly higher compared to proteins containing ochre (UAA) or opal (UGA) stop codons (Figure 2B). Additionally, amber suppressed proteins were enriched independently of their cellular abundance as determined by LC–MS/MS analysis of full proteomes (Supplementary Figure S5B, Supplementary Data 1). Taken together, these data clearly demonstrate that the amberome of mammalian cell lines with an expanded genetic code can be specifically captured and identified by mass spectrometry.

To define the impact of UAG sequence context on amber suppression efficiency, we analyzed the base composition surrounding the termination codons of proteins significantly enriched in pulldowns upon amber suppression. In particular, we focused our analysis on nucleotides 6 base pairs (bp) up- and downstream of the stop codon (nucleotides –6 to –1 and +4 to +9; stop codon at +1, +2, +3) as nucleotides up to the +9 position have been reported to substantially influence translational termination in mammalian cells (60,63). To predict context-specific ncAA incorporation rates at amber stop codons *in silico*, we adapted a linear regression model that has been previously applied to assess genome-wide translational readthrough at stop codons (61). In this approach, significantly enriched stop codon contexts are encoded in a multi-dimensional binary vector space and correlated with their relative fold change determined by SORT-E. In addition to the sequence context, we also included the GC content, which has been reported as one of the most informative structural features governing eukaryotic translational readthrough *in vitro* (64). We first computed linear regression models of UAG contexts separately for mESCs and HEK293T cells. Importantly, HEK293T fold changes measured by SORT-E were linearly correlated with values calculated by the mESC regression model and *vice versa* (Supplementary Figure S5C). This reciprocal validation with data from mESCs or HEK293T cells both confirms the reliability of the regression models to predict relative ncAA incorporation efficiencies and also indicates that amber suppression efficiency is subject to similar context effects in mESCs and HEK293T cells. By combining SORT-E data from mESC and HEK293T cell lines, we then computed a mammal-specific regression model that predicts ncAA incorporation efficiency based on sequence context, which we refer to as iPASS (Identification of Permissive Amber Sites for Suppression).

To extract sequences permitting high amber suppression efficiencies according to iPASS, we weighted each 12-mer amber stop codon context (nucleotides –6 to –1 and +4 to +9) by its iPASS score and computed their probability logo using kpLogo (92) (Figure 2C). Whereas the UAGC tetranucleotide is largely underrepresented as a termination codon in mammalian genes (43), we detected an enrichment of +4 C. Interestingly, +4 C has been described as one of the strongest predictors of translational readthrough by near-cognate tRNAs (63,64,66). Moreover, in the presence of an amber suppressor tRNA, particularly the UAGC tetranucleotide has been reported to permit above-average amber suppression in mammalian cell lines (63,67,68). Additionally, we found purines depleted at the +4 position of efficiently amber suppressed proteins. Interestingly, +4 purines are thought to stabilize formation of the termination complex (101,102) and are generally associated with low translational readthrough in mammalian cells (61,63,66). Remarkably, we detected the strongest nucleotide enrichment at +6 for C as well as depletion of A. In general, distinct enrichment of nucleotides across all positions investigated indicate that ncAA incorporation efficiency is not determined by the identity of a single nucleotide, but rather modulated by a synergistic interplay of nucleotides surrounding the amber stop codon. To further characterize the relative impact of each position on ncAA incorporation efficiency, we performed feature selection by successively removing positions with the smallest contribution to the regression error (Supplementary Figure S5D). Eliminating positions that flank the stop codon gradually increased the regression error, with +4 and –3 having the strongest effect. Therefore, reducing the number of iPASS input values decreases the accuracy of prediction, which highlights the relative importance of flanking sequences on ncAA incorporation efficiency. In summary, by quantifying BcnK incorporation at endogenous amber stop codons in mESCs and HEK293T cells, we developed a linear regression model called iPASS revealing a synergistic influence of the surrounding sequence context on ncAA incorporation efficiency in mammalian cells.

### Development of a dual-fluorescence reporter to identify permissive ncAA incorporation sites

**Figure 3.**
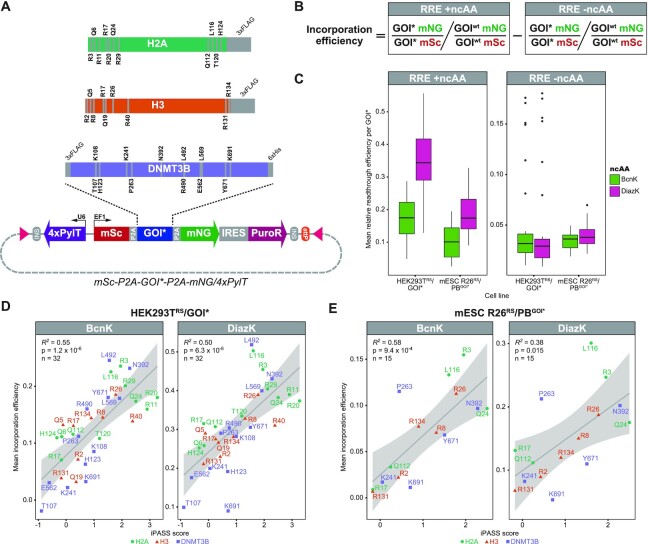
(**A**) Dual-fluorescence reporter construct mSc-P2A-GOI*-P2A-mNG/*4xPylT* to assess ncAA incorporation efficiencies across selected positions within target proteins by flow-cytometry. Expression of the gene of interest with an in-frame amber stop codon (GOI*) is linked to mScarlet (mSc) and mNeonGreen (mNG) expression levels via the self-cleaving peptide 2A (P2A). Amber mutants of the *Mus musculus* histone H2A/H3 with a C-terminal 3xFLAG-tag or the *de novo* DNA methyltransferase 3b (DNMT3B) with an N-terminal 3xFLAG-tag and C-terminal 6xHis-tag were integrated as GOI*. Analyzed amber stop codon positions within each GOI* are indicated. The vector harbors the *attP* attachment site as well as inverted terminal repeats (ITRs, indicated as rectangles) for Bxb1 or PiggyBac (PB) mediated stable integration. Abbreviations: *Methanosarcina mazei* tRNA^Pyl^_CUA_ (PylT), internal ribosomal entry site (IRES), puromycin resistance (PuroR), constitutive EF1α promoter (EF1), constitutive U6 promoter (U6), insulator (INS). (**B**) Formula to calculate the incorporation efficiency of the respective ncAA at each amber stop codon position (GOI*) relative to the wild-type codon (*GOI^wt^*). For each construct, the mean fluorescence intensity (MFI, see also Supplementary Figure S7C and S8C for representative flow-cytometry data) of mNG (GOI* or *GOI^wt^* mNG) is normalized to the respective MFI of mSc (GOI* or *GOI^wt^* mSc). The relative readthrough efficiency (RRE) in the absence of an ncAA (-ncAA) is subtracted from the RRE +ncAA to account for basal translational readthrough over the stop codon that results in full-length peptides lacking the respective ncAA. (**C**) RREs are in total higher in stable HEK293T^RS^ cells and with DiazK. Mean RRE (*n* = 3 biological replicates) at each analyzed GOI* site for DiazK and BcnK (+ncAA) or the -ncAA control was calculated for HEK293T cells stably expressing the respective PylRS variant and transiently transfected with the mSc/mNG fluorescent reporter (HEK293T^RS^/GOI*, *n* = 32) or mESCs stably expressing both PylRS variant and mSc/mNG fluorescent reporter construct (mESC R26^RS^/PB^GOI*^, *n* = 15). Per replicate, mNG and mSc MFIs from ca. 10 000 mSc positive single cells (mSc positive single cell counts are listed in Supplementary Data 2) were acquired by flow-cytometry 24 h after addition of 0.5 mM ncAA (for gating strategy and representative flow-cytometry data see Supplementary Figure S7 and S8). Horizontal black lines within boxes represent median values, boxes indicate the lower and upper quartiles, and whiskers indicate the 1.5 interquartile range. (**D, E**) iPASS reliably predicts relative ncAA incorporation efficiencies at GOI* mutants in mammalian cells. iPASS scores of each GOI* sequence context were correlated with experimentally determined mean incorporation efficiencies (*n* = 3 biological replicates, see also Supplementary Figure S9) of DiazK and BcnK using the mSc-P2A-GOI*-P2A-mNG/*4xPylT* fluorescent reporter in HEK293T^RS^/GOI* (D) or mESC R26^RS^/PB^GOI*^ (E) lines. Coefficient of determination (*R^2^*), *P*-value (*P*), and number (*n*) of analyzed mSc/mNG fluorescent reporters harboring different GOI* are indicated. The 95% confidence interval of the regression line is marked.

To further validate the accuracy of the iPASS model in predicting UAG context-dependent ncAA incorporation efficiencies, we developed a dual-fluorescence reporter to experimentally assess the efficiency of amber suppression across multiple incorporation sites within a target protein in living cells. In combination with flow-cytometry, dual-fluorescence reporters harboring the amber stop codon within the linker region were recently evaluated in yeast to accurately measure ncAA incorporation efficiencies (72). In contrast to previously developed reporters, we constructed a PB and Bxb1-compatible mSc-P2A-GOI*-P2A-mNG/*4xPylT* reporter construct, in which 2A peptides cotranslationally cleave the ncAA-containing protein of interest (POI^ncAA^) from two flanking monomeric fluorescent proteins, mScarlet (mSc) and mNeonGreen (mNG) (Figure 3A). As such, our reporter can be used to assess ncAA incorporation efficiency at a specific site within any user-defined POI^ncAA^. Both mSc and mNG exhibit superior brightness and a favorable maturation speed compared to their conventional spectral counterparts mRFP and GFP/YFP, respectively (103,104). The physiological concentration of a fluorescent protein is directly proportional to its fluorescence intensity (105,106) and amber suppression of the GOI* leads to the equimolar translation of mSc and mNG. Importantly, cotranslational cleavage by 2A peptides uncouples mSc and mNG from POI^ncAA^ turnover rates. Therefore, the ratio between mSc and mNG fluorescence can be calculated to compare amber suppression efficiency between different GOI* mutants independently of the respective POI^ncAA^ stability. Furthermore, translational reinitiation downstream of an in-frame UAG has been reported to occur within the first 70 codons in yeast (107) and the first 160 codons in mammalian cells (108), leading to the leaky expression of N-terminally truncated proteins. By encoding *mSc* upstream of the GOI*, the resulting transcript lacks an in-frame UAG within the first 250 codons following the initial start codon. Hence, translational initiation at secondary start codons downstream of an in-frame UAG should be reduced. This decrease in the aberrant expression of N-terminally truncated POIs might improve yields of POI^ncAA^ that harbor ncAA incorporation sites close to the N-terminus.

We placed amber stop codons into the coding sequences of *H2A*, *H3*, and *Dnmt3b* so that they could also be used for ncAA-mediated crosslinking to identify interaction partners. For H2A and H3, we selected positions within the N- and C-terminal tails of each histone that are in close proximity to post-translationally modified lysine residues, like H3K9, H3K27 or H2AK119 (109). For *Dnmt3b*, we selected weakly conserved positions in the N-terminus and highly conserved residues within interaction surfaces (Supplementary Figure S6). To assess amber suppression at each position, mSc/mNG fluorescent reporter constructs harboring these GOI* amber mutants (H2A*, H3*, or Dnmt3b*) (Figure 3A) were transiently transfected into HEK293T^RS^ cell lines (denoted as HEK293T^RS^/GOI*) or stably integrated into R26^RS^ mESC lines by PB-mediated transposition (denoted as R26^RS^/PB^GOI*^). Cell lines were subsequently cultured in the presence of either 0.5 mM BcnK or DiazK to also assess the incorporation efficiency of an ncAA with a chemical moiety distinct from BcnK. After 24 h, mSc and mNG fluorescence intensities were recorded by flow-cytometry in biological triplicates (Supplementary Figure S7 and S8). Using mSc and mNG mean fluorescence intensities, relative readthrough efficiencies (RREs) (72–74) were calculated for each position in the presence or absence of an ncAA by normalization to the respective wild-type *GOI* (*GOI^wt^*) expression levels (Figure 3B). DiazK and BcnK were better incorporated in HEK293T^RS^/GOI* compared to mESC R26^RS^/PB^GOI*^. In both cell lines, we found that DiazK yielded generally higher RREs than BcnK (Figure 3C), in line with the differences observed after transient transfection of the 4x*PylT*/*mCh-TAG-EGFP* reporter in R26^RS^ (Figure 1C). As readthrough events caused by near-cognate tRNAs might obscure the true efficiency of ncAA incorporation, we sought to account for this by calculating a corrected incorporation efficiency at each position, where the RRE –ncAA is subtracted from the RRE +ncAA (Figure 3B). Single incorporation efficiency measurements were highly reproducible between biological triplicates, confirming the stringency of the mSc/mNG fluorescent reporter assay (Supplementary Figure S9A+B). Within H2A*, H3*, and Dnmt3b* amber mutants, measured incorporation efficiencies maximally varied between 4.2- and 33-fold for BcnK and 2.4- and 11-fold for DiazK (Supplementary Figure S9C). Additionally, these variations seemed to be independent of the distance between UAG position and PolyA tail. We observed incorporation efficiencies to differ more than 2-fold between proximal (e.g. H2A^Q112*^ and H2A^L116*^) and even adjacent (e.g. Dnmt3b^T107*^ and Dnmt3b^K108*^) UAG positions, highlighting the distinct influence of the immediate sequence context on amber suppression by PylT. The varying incorporation efficiencies at selected positions as well as cleavage of 2A peptides were also verified by western blot (Supplementary Figure S10A). Although P2A peptides have been reported to be efficiently cleaved (105), we detected a small fraction of uncleaved fusion proteins for all tested constructs. Furthermore, overall expression of N-terminal mSc in the absence of an ncAA was generally lower in amber mutants compared to wt coding sequences in both HEK293T^RS^/GOI* and mESC R26^RS^/PB^GOI*^ (Supplementary Figure S10B). This reduction in mSc levels might be due to exon-junction complex-independent nonsense-mediated decay (NMD) of these intron-free, nonsense transcripts (110). Conversely, after ncAA addition, amber mutant constructs exhibited up to 2-fold higher mSc levels compared to their respective *GOI^wt^* constructs (Supplementary Figure S10B). These increases in mSc were linearly correlated with changes in mNG intensity and, as such, suppression of the amber stop codon (Supplementary Figure S10C), suggesting that PylT-mediated suppression of in-frame UAG and incorporation of ncAAs stabilize nonsense transcripts and/or enhance translational efficiency in mammalian cells. While the underlying molecular mechanisms remain unclear, this unexpected observation highlights the methodological advantage of dual- over C-terminal single-fluorescence reporters to accurately assess relative incorporation efficiencies by accounting for varying reporter construct expression levels between different conditions. Taken together, we demonstrate that our mSc/mNG fluorescent reporter used in conjunction with flow-cytometry analysis offers a rapid and reliable means for the high-throughput characterization of ncAA incorporation efficiency at different sites within a GOI*.

### Validation of the iPASS model with experimentally determined ncAA incorporation efficiencies

To verify the iPASS model, we directly compared the predicted iPASS score of amber stop codon contexts for each GOI* with their experimental ncAA incorporation efficiencies measured with the mSc/mNG fluorescent reporter in living cells. For both HEK293T^RS^/GOI* and mESC R26^RS^/PB^GOI*^, predicted and measured incorporation efficiencies are linearly correlated, confirming the accuracy and reliability of iPASS in identifying permissive ncAA incorporation sites based on UAG sequence contexts (Figure 3D and E). In general, low-scoring (e.g. H2A^R17*^, H3^R131*^, Dnmt3b^K241*^) and high-scoring (e.g. H2A^R3*^, H3^R26*^, Dnmt3b^N392*^) GOI* contexts were also associated with relatively low or high ncAA incorporation efficiencies, respectively (Figure 3D and E). Of note, some GOI* contexts with similar iPASS scores still varied several fold in their incorporation efficiencies (e.g. Dnmt3b^K691*^ and Dnmt3b^L492*^ in HEK293T^RS^; Figure 3D, Supplementary Figure S9A), indicating the existence of additional factors influencing amber suppression that are not accounted for in the iPASS model. By randomly re-assigning fold changes determined by SORT-E to all detected sequence contexts, we also computed a decoy linear regression model. The decoy model fails to predict measured ncAA incorporation efficiencies in HEK293T and mESC lines (Supplementary Figure S11A and B). This negative control further highlights the specificity of iPASS to predict *bona fide* incorporation efficiencies according to favorable UAG contexts. Importantly, the capacity of iPASS to identify permissive ncAA sites for both BcnK and DiazK incorporation in mESC as well as HEK293T cell lines indicates that relative amber suppression with PylT is predominantly influenced by mRNA context rather than ncAA or cell line identity. Accordingly, incorporation efficiencies measured with the mSc/mNG fluorescent reporter were also linearly correlated between mESCs R26^RS^ and HEK293T^RS^ as well as between BcnK and DiazK (Supplementary Figure S11C and D). Hence, once identified, positions with high incorporation efficiencies can be readily modified with diverse ncAAs across different mammalian cell types. In conclusion, the iPASS model reliably predicts relative ncAA incorporation efficiencies at different UAG sequence contexts in mammalian cell lines expressing the *PylS*/*PylT* pair.

### iPASS guided optimization of ncAA incorporation efficiencies by silent mutation of flanking codons

**Figure 4.**
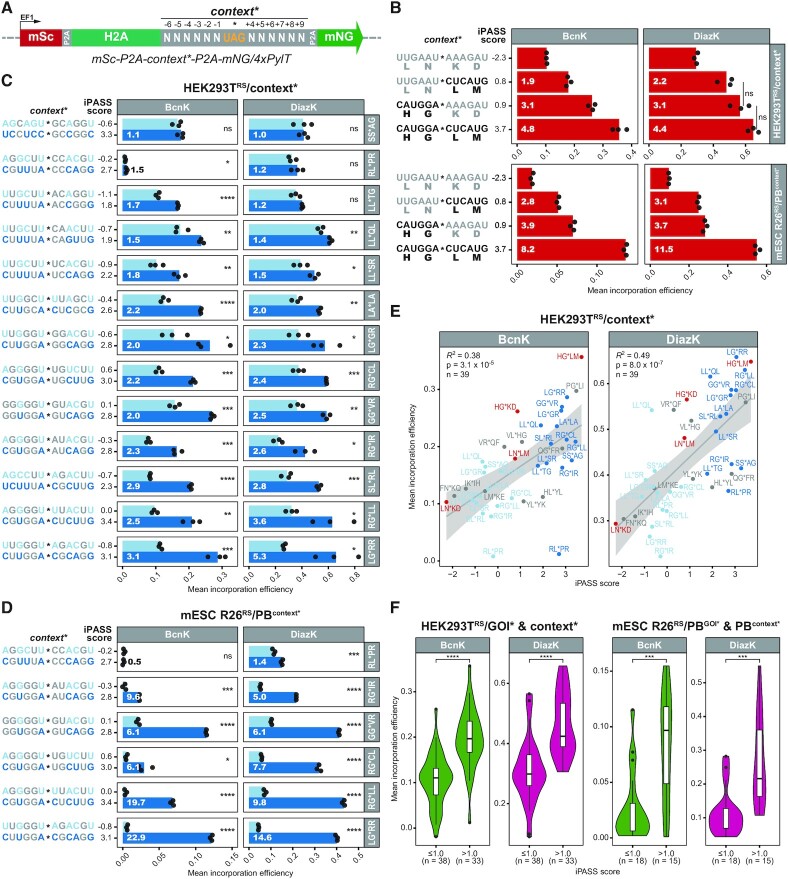
(**A**) Segment of mSc/mNG dual-fluorescence reporter construct mSc-P2A-context*-P2A-mNG/*4xPylT* (see also Figure 3A) to assess ncAA incorporation efficiencies at amber stop codons within selected sequence contexts (context*) by flow-cytometry. The N-terminal 3xFLAG-tag of *H2A^wt^* within the mSc/mNG fluorescent reporter is replaced with context* composed of selected nucleotides (N) 6 bp up- and downstream of the amber stop codon (nucleotides –6 to +9; stop codon at +1, +2, +3). Incorporation efficiencies are calculated according to Figure 3B with context* and *context^wt^* replacing GOI* and *GOI^wt^*, respectively. In *context^wt^* constructs the amber stop codon (UAG) is changed to the lysine codon (AAG) (not shown). Abbreviations: self-cleaving peptide 2A (P2A), constitutive EF1α promoter (EF1). (**B–E**) Mean incorporation efficiencies (*n* = 3 biological replicates) of DiazK or BcnK at context* were calculated using the mSc-P2A-context*-P2A-mNG/*4xPylT* fluorescent reporter in HEK293T cells stably expressing the respective PylRS variant and transiently transfected with the fluorescent reporter (HEK293T^RS^/context*) or mESCs stably expressing both PylRS variant and fluorescent reporter construct (mESC R26^RS^/PB^context*^). Per replicate, mNG and mSc mean fluorescence intensities from 5000 to 10 000 mSc positive single cells (mSc positive single cell counts are listed in Supplementary Data 2) were acquired by flow-cytometry 24 h after addition of 0.5 mM ncAA. (B–D) According to Smith and Yarus (114), the fold change between incorporation efficiencies (IEs) at two different amber stop codon sequence contexts is calculated as {IE(max) × [1-IE(min)]}/{IE(min) × [1 – IE(max)]}. For each context* the nucleotide sequence ±6 bp flanking the amber stop codon (*) and its respective iPASS score are presented. Two-tailed unpaired two-sample Student's *t*-test: ns not significant, *P < 0.05, **P < 0.01, ***P < 0.001, ****P < 0.0001. (**B**) Incorporation efficiency is synergistically influenced by the six nucleotides up- as well as downstream of the amber stop codon and varies several fold between context* with the lowest and highest iPASS score. White number within bars indicates fold change compared to the LN*KD context with the lowest iPASS score. Two-tailed unpaired two-sample Student's *t*-test: ns not significant, all other context* at least *P* < 0.01 (not indicated). (**C, D**) The iPASS tool guides silent mutation of contexts* improving incorporation efficiencies several fold in HEK293T^RS^/context* (C) or mESC R26^RS^/PB^context*^ (D) lines. White number within dark blue bars (optimized context*) indicates fold change compared to the same amino acid context with a lower iPASS score (light blue bars). (**E**) iPASS reliably predicts relative ncAA incorporation efficiencies at context* mutants in mammalian cells. iPASS scores of each context* target site were correlated with experimentally determined mean incorporation efficiencies of DiazK or BcnK in HEK293T^RS^/context* cell lines. Coefficient of determination (*R^2^*), *P*-value (*P*), and number (*n*) of analyzed mSc/mNG fluorescent reporters harboring different context* are indicated. The 95% confidence interval of the regression line is marked. Color coding according to (B)–(D) (gray: additional context* from Supplementary Figure S12A). (**F**) An iPASS score >1.0 generally indicates higher incorporation efficiencies compared to an iPASS score ≤1.0 (Mean[iPASS score] ≈ 1.0). Mean incorporation efficiencies at each analyzed GOI* and context* site for DiazK and BcnK in HEK293T^RS^ or mESC R26^RS^ are grouped according to their iPASS score. Total number (*n*) of analyzed mSc/mNG fluorescent reporters in each group are indicated. Inside violin plots horizontal black lines within boxes represent median values, boxes indicate the lower and upper quartiles, and whiskers indicate the 1.5 interquartile range. Two-tailed unpaired two-sample Student's *t*-test: ***P < 0.001, ****P < 0.0001.

After generally assessing the predictive power of iPASS, we next wondered whether ncAA incorporation efficiencies can be improved by silently mutating the codons flanking the amber stop codon. To this end, we replaced the C-terminal 3xFLAG-tag of *H2A^wt^* within the mSc/mNG dual-fluorescence reporter (Figure 3A) with selected nucleotide contexts spanning the 6 bp up- and downstream of the amber stop codon (contexts*) (Figure 4A). As before, these mSc-P2A-context*-P2A-mNG/4xPylT reporters were transiently transfected into HEK293T^RS^ (denoted as HEK293T^RS^/context*) or stably integrated into R26^RS^ mESCs by PB-mediated transposition (denoted as R26^RS^/PB^context*^) to identify context* dependent variations in BcnK or DiazK incorporation efficiencies by flow-cytometry analysis.

We first asked whether the nucleotides preceding or following the amber stop codon determine incorporation efficiency. Within the ribosome, the codons preceding the amber stop codon have already been decoded into amino acids or are bound in the ribosomal P-site by a peptidyl-tRNA, whereas downstream codons have yet to be translated. This biomechanical difference between up- and downstream contexts might differentially affect PylT decoding at amber stop codons. However, the iPASS model suggests a synergistic influence of the surrounding sequence context on ncAA incorporation efficiency (Figure 2C). To better understand the relative importance of up- versus downstream nucleotides, we applied iPASS to extract the context* with the lowest or highest iPASS score. Additionally, we exchanged either the up- or downstream sequence with the lowest iPASS score for the sequence with the highest iPASS score, thereby constructing two chimeric contexts* with intermediate iPASS scores (Figure 4B). Compared to the context* with the lowest iPASS score, incorporation efficiencies significantly increased for both chimeric contexts*. In particular, replacing the preceding nucleotides improved incorporation efficiencies to a greater extent than the nucleotides following the amber stop codon. However, fully replacing the low-score with the high-score context* increased incorporation efficiencies even further, up to 4.8- or 11.5-fold in HEK293T^RS^ or mESCs R26^RS^, respectively. Although the preceding nucleotides may have a greater impact, these results confirm that both up- and downstream nucleotides synergistically influence ncAA incorporation efficiency.

We then tested whether iPASS can be applied to optimize amber suppression within a given amino acid sequence by silently mutating the two codons flanking the stop codon. This strategy would be useful in amber suppression applications where the ncAA incorporation site is usually fixed, such as the installation of post-translationally modified ncAAs or the incorporation of ncAAs to probe enzyme active sites. For this, we analyzed context* pairs displaying a minimal difference of 2.4 in iPASS score after iPASS guided synonymous codon exchange. BcnK as well as DiazK incorporation significantly increased in 10 out of 13 iPASS optimized contexts* in HEK293T^RS^/context* cells, ranging from 1.4- up to 5.3-fold (Figure 4C). Furthermore, fold changes of five out of six selected context* pairs in stable mESC R26^RS^/PB^context*^ cell lines were generally higher than in HEK293T^RS^ cells, ranging between 5.0- and 22.9-fold (Figure 4D). Notably, even though iPASS optimization did not improve incorporation efficiencies for all contexts*, we did not detect a reduced ncAA incorporation efficiency after iPASS guided optimization. Consistent with reports that +4 C permits above-average amber suppression in mammalian cells (63,67,68), we observed in HEK293T^RS^ and mESCs R26^RS^ the highest fold changes for iPASS optimized contexts* in which +4 A or +4 U was exchanged with +4 C. Accordingly, the overall efficiency of DiazK incorporation into all GOI* (Supplementary Figure S9A+B) and context* (Figure 4B-D, Supplementary Figure S12A) mutants analyzed was significantly higher for +4 C than for the remaining three +4 nucleotides (Supplementary Figure S12B). At the same time, iPASS also successfully optimized contexts* without altering the +4 base, further confirming that the influence of nucleotides on amber suppression extends over the +4 position. Taken together, we demonstrate that an approximate iPASS score difference of 2.5–3.0 after synonymous exchange of codons flanking the amber stop codon usually increases ncAA incorporation efficiencies.

To further validate the iPASS model, we compared the experimentally determined incorporation efficiencies of different contexts* with their respective iPASS scores, revealing a maximal coefficient of determination (*R^2^*) of 0.49 and 0.56 in HEK293T^RS^ and mESCs R26^RS^ lines, respectively (Figure 4E, Supplementary Figure S12C). Interestingly, also translational readthrough at contexts* by near-cognate tRNAs in the absence of ncAAs was linearly correlated with suppression by PylT upon ncAA addition, although with in total lower *R^2^* values compared to iPASS, ranging from 0.24 to 0.43 (Supplementary Figure S12D+E). This correlation argues for overall similar context effects in amber suppression by PylT and near-cognate tRNAs. Additionally, incorporation efficiencies of both ncAAs as well as both analyzed cell lines were linearly correlated, further confirming that amber suppression efficiencies are largely independent of ncAA and cell line identity (Supplementary Figure S12F and G). Across all GOI* and contexts* analyzed with the mSc/mNG fluorescent reporter, an iPASS score >1.0 (approximate mean of iPASS score ranging from –2.3 to 3.7) is generally associated with significantly increased incorporation efficiencies (Figure 4F). Hence, an iPASS score cut-off of 1.0 should be applied when screening for permissive amber suppression sites in a target open reading frame. In summary, iPASS not only reliably aids the identification of permissive amber suppression sites, but also guides the silent mutation of flanking codons to optimize ncAA incorporation at a selected site.

## DISCUSSION

Readthrough of amber stop codons in mammalian cells by near-cognate tRNAs is governed by the identity of flanking nucleotides (51,54,55,61,63,66) with a limited dataset indicating similar but not identical context effects in the decoding capacity of an amber suppressor tRNA (63). Depending on the location of the amber stop codon within a given sequence, these context effects lead to highly variable ncAA incorporation rates. Here, we have not only investigated to which extent UAG contexts influence ncAA incorporation rates but also provide a streamlined workflow that combines analysis *in silico* and in living mammalian cells to quickly and reliably identify permissive ncAA incorporation sites.

We combine two previously described strategies (30,76) to establish a vector system that is compatible with both site-specific recombination by Bxb1 as well as PB transposition. We then use this system to expand the genetic code of both murine and human cell lines. In particular, we leverage Bxb1-mediated recombination to integrate the *PylS*/*PylT* pair at the genomic safe harbor R26 in mESCs and subsequently apply PB transposition to genomically integrate multiple copies of the GOI*. This stepwise approach has the advantage that the tRNA synthetase expression level should be lower in comparison to GOI* and *PylT* expression levels, which has been reported to enhance amber suppression efficiency in mammalian cells (27). Collectively, we demonstrate that with this strategy stable and defined mESC clones that efficiently suppress amber stop codons can be established. Of note, in contrast to a recent report applying amber suppression with BocK (111), we observed no directed cellular response to amber suppression with BcnK. This discrepancy might be due to more stringent filtering of our full proteome data and the lower incorporation efficiency of BcnK compared to BocK.

To date, application of dual-fluorescence reporters has been limited to comparisons of amber suppression efficiencies among different OTSs and hosts (72–74) but not GOI* mutants. Reporters such as *mCherry-TAG-EGFP* are useful to assess amber suppression efficiencies of newly developed synthetases and ncAAs. However, adaptation and application of these reporters in mammalian cells to quickly compare ncAA incorporation rates among defined sites within a target protein has not been explored. Our mSc/mNG fluorescent reporter bearing a P2A flanked GOI* in combination with high-throughput analysis by flow-cytometry reproducibly yields these context-specific ncAA incorporation efficiencies. For all GOI* and both ncAAs tested, the reporter detects several fold differences in ncAA incorporation efficiency even between close-by amber sites. Importantly, turnover rates of fluorescent proteins are uncoupled via self-cleaving 2A peptides to selectively evaluate ncAA incorporation independently of POI^ncAA^ stability. Furthermore, this vector system can be readily used to generate mammalian cell lines stably expressing a tag-free POI^ncAA^ for downstream applications. Taken together, the sensitive and consistent quantification of ncAA incorporation efficiencies renders the mSc/mNG fluorescent reporter a valuable tool to rapidly identify highly permissive ncAA incorporation sites.

To date, optimization of genetic code expansion in mammalian cells has been mostly focused on intrinsic properties of the OTS itself, for instance by engineering OTS components or tuning their expression levels. UAG context-dependent variations in ncAA incorporation efficiencies, on the other hand, require time-consuming cloning and screening of multiple amber mutants for each individual POI^ncAA^. We reasoned that quantifying ncAA incorporation rates at endogenous amber stop codons might provide generalizable insights into the relationship between UAG flanking sequences and ncAA incorporation efficiencies. By adapting SORT-E (75) with BcnK and Biotin-Tet to amber codons, we endogenously probe BcnK incorporation at hundreds of potential sequence contexts in mESCs and HEK293T cells. As our regression model encompasses the six nucleotides up- and downstream of significantly enriched amber stop codon contexts, the potential contribution of extended RNA secondary structures on ncAA incorporation efficiency is omitted. However, both regression models calculated for HEK293T or mESCs reciprocally correlate with experimental mESC or HEK293T SORT-E data, justifying this focus on close-by nucleotides. Furthermore, this restriction to the proximal nucleotides widely facilitates the selective modification of UAG contexts to adopt high ncAA incorporation efficiencies. By combining the HEK293T and mESC regression model, we formulate iPASS to predict relative ncAA incorporation efficiencies at amber stop codons based on the surrounding nucleotide context. Probability logo representation of all possible sequence contexts weighted by their iPASS score indicates that nucleotides up- as well as downstream of the stop codon affect ncAA incorporation. Removing nucleotide positions within iPASS gradually reduced the accuracy of relative ncAA incorporation efficiency prediction, which excluded potential overfitting of the iPASS model. By separately optimizing up- and downstream UAG contexts using iPASS and measuring their suppression efficiencies with the mSc/mNG fluorescent reporter, we experimentally confirm that nucleotides on both sides of UAG govern the efficiency of amber suppression. Thus, the identity of nucleotides flanking the amber stop codon synergistically influences ncAA incorporation efficiencies in mammalian cells.

It is tempting to speculate that UAG contexts with high translational readthrough also boost ncAA incorporation rates. However, the iPASS probability logo, despite encompassing the readthrough promoting feature +4 C, widely differs at the majority of nucleotide positions from similar linear regression analyses of readthrough motifs in human cells (61,66). Additionally, these previously reported stop codon contexts are enriched for the opal codon and hence motifs that might not promote translational readthrough in the context of amber stop codons. The iPASS motif also differs from bacterial UAG contexts with high amber suppression efficiencies (33), confirming distinct context effects between pro- and eukaryotes. An established amber stop codon context that confers strong translational readthrough *in vitro* and *in vivo* in eukaryotes is the consensus sequence +1 UAG CAR YYA (48–51,53,55,64), a readthrough motif which has been originally identified in the tobacco mosaic virus (TMV) as +1 UAG CAA UUA (112). However, the iPASS motif, although enriched for +4 C and +7 UUA, is depleted of +5 A and especially +6 R. Interestingly, translational readthrough at UAG contexts in the absence of an ncAA was yet roughly correlated with their suppression efficiency. Consistent with a previous report (63), this result indicates that amber suppression by PylT and translational readthrough by near-cognate tRNAs are influenced by similar but not identical flanking sequence preferences. Hence, the relative ncAA incorporation efficiency at target sites might benefit from sequence features permitting strong translational readthrough. Importantly, the identity of eukaryotic near-cognate tRNAs and their preferences for specific stop tetranucleotides have been reported to be interdependent (62,65). This observation implies that context-specific differences in the capacity of PylT to decode in-frame UAGs might be at least partially attributable to intrinsic PylT properties. Therefore, stop codon contexts would have to be specifically adapted to promote either readthrough by near-cognate tRNAs or suppression by PylT. Accordingly, iPASS might not reliably predict ncAA incorporation efficiencies at amber stop codons by OTSs other than the *PylS*/*PylT* pair in mammalian cells. However, our strategy of regression analysis after SORT-E together with the fluorescent reporter assay can easily be expanded for other OTSs as well as to opal, ochre, or quadruplet codons to identify favorable sequence contexts for efficient decoding.

Using incorporation efficiencies measured with the mSc/mNG fluorescent reporter, we validate the iPASS model to predict relative ncAA incorporation rates *in silico* depending on the UAG context. In particular, we experimentally determined ncAA incorporation efficiencies across multiple sites within a GOI* as well as at a fixed context* site with varying nucleotide compositions. In both experimental setups, analysis of UAG contexts with iPASS accurately reveals approximately half of the variation in amber suppression efficiency by PylT in mammalian cells, suggesting that efficient suppression is largely governed by sequence context. However, we detected some positions at which iPASS did not accurately predict ncAA incorporation efficiencies. Hence, additional factors not covered by iPASS might influence context-specific efficiency of amber suppression by PylT, such as mRNA abundance, translational speed and pausing, or structural and more distant sequence features surrounding UAG. For instance, besides close-by nucleotides also RNA secondary structures (64) and sequence features more than 6 bp downstream of an in-frame stop codon (60) were reported to promote eukaryotic translational readthrough. In general, however, UAG contexts with an iPASS score >1.0 confer significantly higher ncAA incorporation rates than contexts with a lower iPASS score. This iPASS guided pre-selection considerably reduces the number of amber mutants that must be cloned and screened to identify permissive ncAA incorporation sites.

Moreover, iPASS can be used to reliably optimize amber suppression efficiencies by synonymous codon exchange. In general, only three amino acids (Arg, Leu, Ser) allow for synonymous codon exchange by modifying the first base and only one (Ser) by modifying the second base. This inherent limitation in synonymous codons available for exchange might restrict the capacity of iPASS to improve amber suppression efficiencies at some fixed sites. However, in 6 out of 13 contexts* tested in HEK293T^RS^ cells, optimization by iPASS resulted in significantly improved ncAA incorporation efficiencies without modifying the +4 or +5 base, further highlighting that ncAA incorporation efficiency is influenced by nucleotides beyond these positions. Notably, iPASS also optimized incorporation efficiencies in three out of five contexts* without altering +4 purines, which are thought to stabilize formation of the termination complex (101,102) and are generally associated with reduced translational readthrough (63,66) and suppression (63,67) of an in-frame UAG. Here, we demonstrate that an iPASS score difference >2.5 after synonymous codon exchange generally results in an up to several fold increase in ncAA incorporation efficiency.

Lastly, both measured and predicted relative incorporation efficiencies seem to be independent of cell line and ncAA identity. This finding is consistent with the hypothesis that context effects in amber suppression depend on fundamental properties of mammalian translation (67). Additionally, in *Escherichia coli* the relative ncAA incorporation rate at defined mRNA contexts has been reported to be generally independent of ncAA size and chemical reactivity (31,33,113). Therefore, predicted incorporation efficiencies can be first validated using standard ncAAs and cell lines before continuing with specialized ncAAs and sophisticated cell lines like mESCs.

To the best of our knowledge, the iPASS regression model provides the first characterization of flanking sequences that permit high amber suppression and hence ncAA incorporation rates in mammalian cells. In combination with the mSc/mNG dual-fluorescence reporter, our pipeline streamlines the identification of permissive ncAA incorporation sites. This will greatly facilitate ncAA-based crosslinking and labeling experiments, enabling researchers to select the most efficient sites while reducing the total number of amber mutants that have to be cloned and tested. To assist with the preselection of ncAA incorporation sites in mammalian cell lines expressing the *PylS*/*PylT* pair, we developed the iPASS web-tool that can be accessed at www.bultmannlab.eu/tools/iPASS. The tool additionally guides the design of silent mutations of the nucleotide positions flanking the amber stop codon to improve ncAA incorporation rates. This functionality will be very useful in applications where the incorporation site is fixed, such as the selective installation of ncAAs mimicking post-translational modifications.

## DATA AVAILABILITY

Plasmids cloned in this study have been deposited at Addgene with the IDs 167491–99. Mass spectrometry proteomics data are available via ProteomeXchange with the dataset identifier PXD019815. Flow-cytometry raw data are available via FlowRepository with the dataset identifier FR-FCM-Z2N3. Significantly enriched proteins in the streptavidin pulldown, their relative expression level according to LC–MS/MS full proteome data, encoded stop codon, and molecular weight are available in Supplementary_data_1.xlsx. Data from flow-cytometry of the mSc/mNG dual-fluorescence reporter constructs and respective analysis by iPASS (nucleotide context, iPASS score, mSc positive single cell count, mean fluorescence intensity, (mean) relative readthrough efficiency, (mean) incorporation efficiency) are available in Supplementary_data_2.xlsx. Significantly changed proteins in full proteomes used for GO enrichment analysis are available in Supplementary_data_3.csv. Data to compute iPASS (mean LFC, *P*-value, sequence context) are available in Supplementary_data_4.csv.

## Supplementary Material

gkab132_Supplemental_Files

## Notes

Present address: Geraldine Rodschinka, Max Planck Institute of Biochemistry, Martinsried 82152, Germany.

Present address: Michael Wierer, Proteomics Research Infrastructure, University of Copenhagen, Copenhagen 2200, Denmark.
