# Supplementary material for: Identification of permissive amber suppression sites for efficient non-canonical amino acid incorporation in mammalian cells
Source: Nucleic Acids Res. 2021 Mar 3;49(11):e62. doi: 10.1093/nar/gkab132 (PMC8216290; doi:10.1093/nar/gkab132)
Supplement: gkab132_Supplemental_Files [file gkab132_supplemental_files.zip › Supplementary_Figures (5).pdf]

Identification of permissive amber suppression sites for efficient non-canonical amino acid incorporation in mammalian cells

Michael D. Bartoschek, Enes Ugur, Tuan-Anh Nguyen, Geraldine Rodschinka, Michael Wierer, Kathrin Lang and Sebastian Bultmann

SUPPLEMENTARY FIGURES

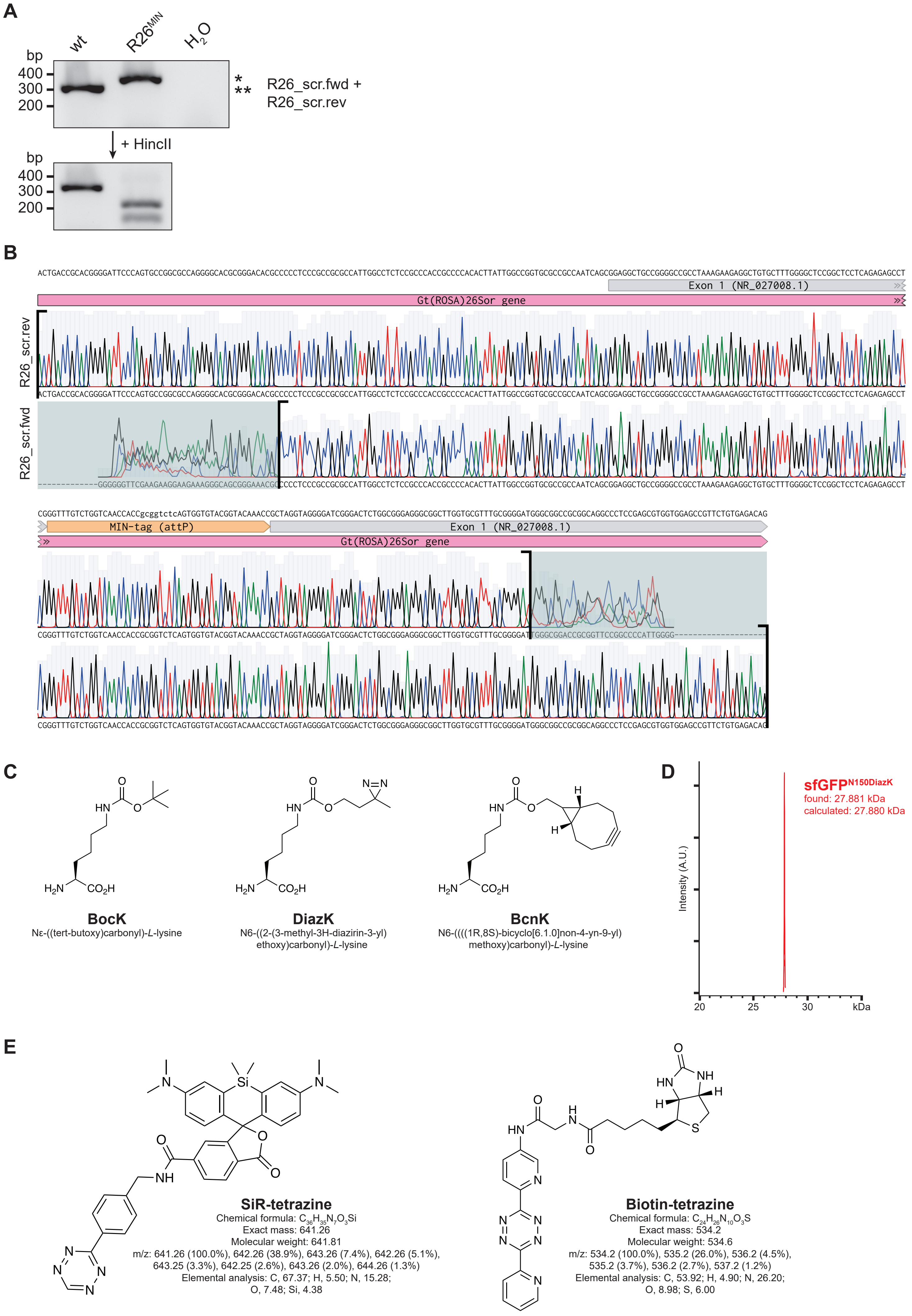
A
wt
R26MIN
H2O
bp
400
300
200
*
**
R26_scr.fwd +
R26_scr.rev
+ HincII
B
Exon 1 (NR_027008.1)
Gt(ROSA)26Sor gene
R26_scr.rev
R26_scr.fwd
MIN-tag (attP)
C
BocK
Nε-((tert-butoxy)carbonyl)-L-lysine
DiazK
N6-((2-(3-methyl-3H-diazirin-3-yl)ethoxy)carbonyl)-L-lysine
BcnK
N6-((((1R,8S)-bicyclo[6.1.0]non-4-yn-9-yl)methoxy)carbonyl)-L-lysine
D
sfGFPN150DiazK
found: 27.881 kDa
calculated: 27.880 kDa
Intensity (A.U.)
20
25
30
kDa
E
SiR-tetrazine
Chemical formula: C36H35N7O3Si
Exact mass: 641.26
Molecular weight: 641.81
m/z: 641.26 (100.0%), 642.26 (38.9%), 643.26 (7.4%), 642.26 (5.1%), 643.25 (3.3%), 642.25 (2.6%), 643.26 (2.0%), 644.26 (1.3%)
Elemental analysis: C, 67.37; H, 5.50; N, 15.28; O, 7.48; Si, 4.38
Biotin-tetrazine
Chemical formula: C24H26N10O3S
Exact mass: 534.2
Molecular weight: 534.6
m/z: 534.2 (100.0%), 535.2 (26.0%), 536.2 (4.5%), 535.2 (3.7%), 536.2 (2.7%), 537.2 (1.2%)
Elemental analysis: C, 53.92; H, 4.90; N, 26.20; O, 8.98; S, 6.00

Supplementary Figure 1: The MIN-tag is stably integrated into the *Rosa26* locus (R26^{MIN}) in mESCs. **(A)** Agarose gel electrophoresis of screening PCRs using the indicated oligonucleotides from Fig. 1A. Homozygous integration of the MIN-tag (*attP* site) into R26 results in a 48 bp shift (*) compared to wt (**) and introduces a HincII restriction site resulting in complete digest of the screening PCR (lower panel). **(B)** Sanger sequencing of screening PCR amplifying R26 exon 1 harboring the MIN-tag using R26_scr.rev (upper lane) or R26_scr.fwd (lower lane) as sequencing primer. **(C)** Non-canonical amino acids BocK, DiazK, and BcnK used in this study. **(D)** DiazK is selectively incorporated in response to the amber stop codon in mammalian cells. Stable HEK293T^{RS_DiazK} were transiently transfected with *4xPylT/sfGFP*^{N150*} and cultured for 72 h with 0.5 mM DiazK. Selective incorporation of DiazK was confirmed by full-length electrospray ionization mass spectrometry (ESI-MS) of immunoaffinity purified sfGFP^{N150DiazK}. **(E)** Tetrazine conjugates used in this study. Silicon rhodamine (SiR).

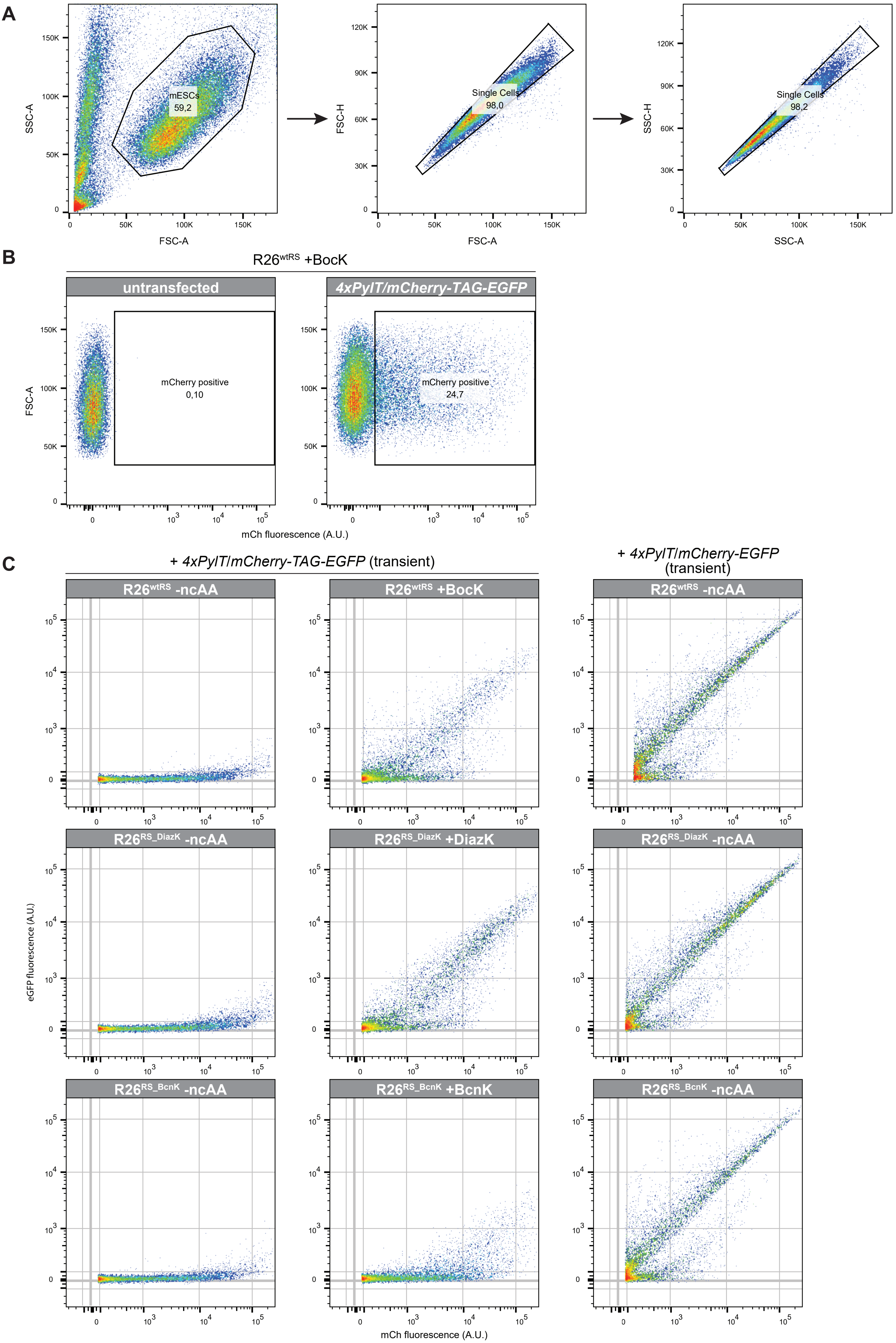
A
mESCs
59,2
SSC-A
FSC-A
Single Cells
98,0
FSC-H
FSC-A
Single Cells
98,2
SSC-H
SSC-A
B
R26wtRS +BocK
untransfected
4xPylT/mCherry-TAG-EGFP
mCherry positive
0,10
mCherry positive
24,7
FSC-A
mCh fluorescence (A.U.)
C
+ 4xPylT/mCherry-TAG-EGFP (transient)
+ 4xPylT/mCherry-EGFP (transient)
R26wtRS -ncAA
R26wtRS +BocK
R26wtRS -ncAA
R26RS_DiazK -ncAA
R26RS_DiazK +DiazK
R26RS_DiazK -ncAA
R26RS_BcnK -ncAA
R26RS_BcnK +BcnK
R26RS_BcnK -ncAA
eGFP fluorescence (A.U.)
mCh fluorescence (A.U.)

Supplementary Figure 2: Representative flow-cytometry data of R26^{wtRS} mESCs transiently transfected with *4xPylT/mCherry-TAG-EGFP* or *4xPylT/mCherry-EGFP*. **(A+B)** Illustration of gating strategy to analyze expression of *4xPylT/mCherry-TAG-EGFP* by flow cytometry after transient transfection of R26^{RS} clones. Here exemplified by stable mESC clone R26^{wtRS} transiently transfected with *4xPylT/mCherry-TAG-EGFP* and cultured for 24 h with 0.5 mM BocK. Debris (FSC-A/SSC-A) and doublets (FSC-A/FSC-H and SSC-A/SSC-H) were excluded (A) and 9,000 mCh positive single cells (B, right panel) per sample analyzed. Untransfected R26^{wtRS} were used as mCh negative control (B, left panel). Numbers indicate the percentage of events within gates. Fluorescence intensities are indicated in arbitrary units (A.U.). Abbreviations: Forward scatter area (FSC-A), forward scatter height (FSC-H), side scatter area (SSC-A), side scatter height (SSC-H). **(C)** Dot plots from Fig. 1C with respective -ncAA controls or transient transfection of the *4xPylT/mCherry-EGFP* reporter as reference of the optimal mCherry/EGFP ratio. R26^{wtRS}, R26^{RS_DiazK}, or R26^{RS_BcnK} mESCs were transiently transfected with *4xPylT/mCherry-TAG-EGFP*, cultured for 24 h without or with 0.5 mM of the indicated ncAA, and 9,000 mCh positive single cells per condition analyzed by flow-cytometry. Fluorescence intensities are indicated in arbitrary units (A.U.).

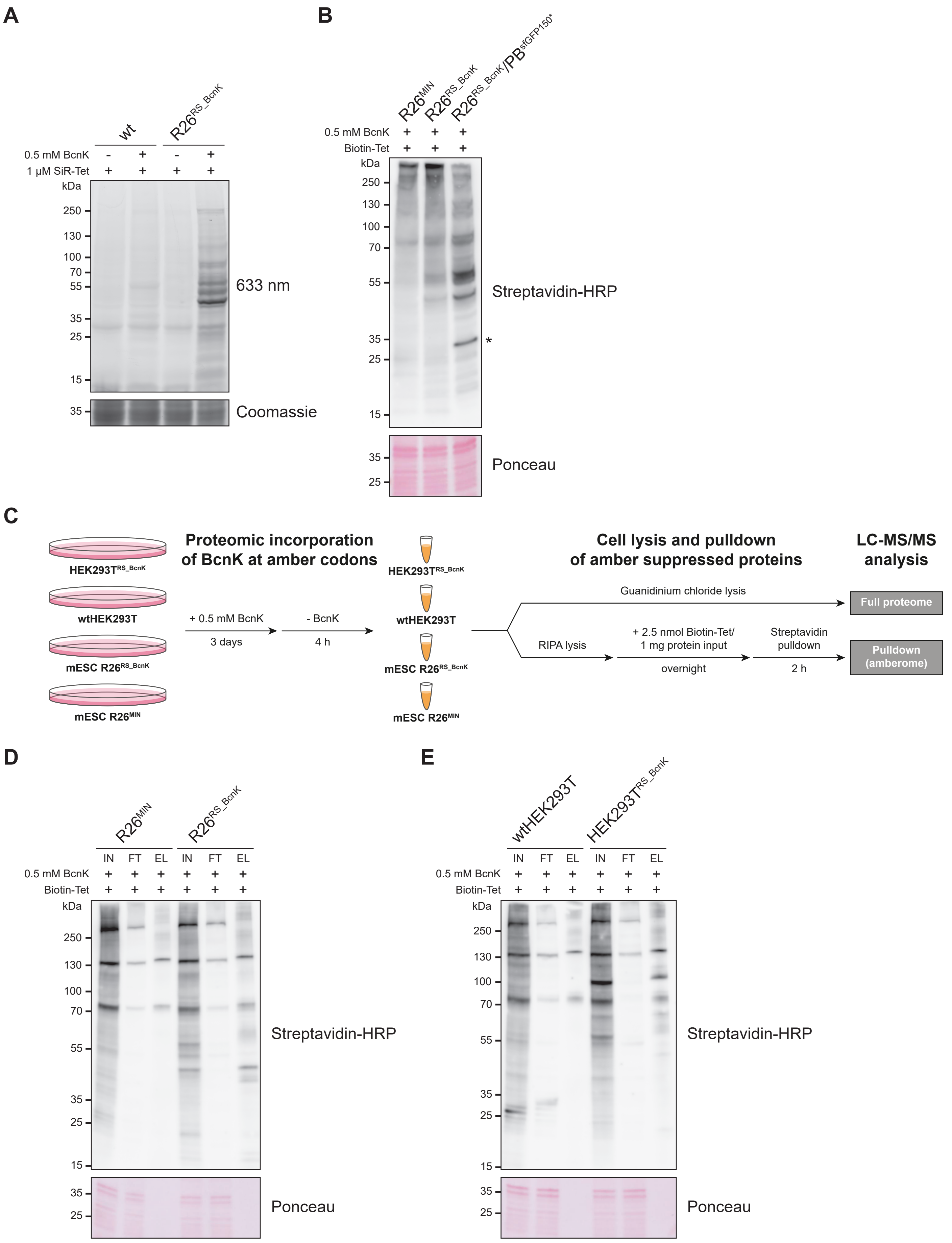
A
wt
R26RS_BcnK
0.5 mM BcnK
1 µM SiR-Tet
kDa
250
130
100
70
55
35
25
15
633 nm
35
Coomassie
B
R26MIN
R26RS_BcnK
R26RS_BcnK/PBsfGFP150*
0.5 mM BcnK
Biotin-Tet
kDa
250
130
100
70
55
35
25
15
Streptavidin-HRP
*
35
25
Ponceau
C
HEK293TRS_BcnK
wtHEK293T
mESC R26RS_BcnK
mESC R26MIN
Proteomic incorporation of BcnK at amber codons
+ 0.5 mM BcnK
3 days
- BcnK
4 h
HEK293TRS_BcnK
wtHEK293T
mESC R26RS_BcnK
mESC R26MIN
Cell lysis and pulldown of amber suppressed proteins
Guanidinium chloride lysis
RIPA lysis
+ 2.5 nmol Biotin-Tet/ 1 mg protein input
overnight
Streptavidin pulldown
2 h
LC-MS/MS analysis
Full proteome
Pulldown (amberome)
D
R26MIN
R26RS_BcnK
IN
FT
EL
0.5 mM BcnK
Biotin-Tet
kDa
250
130
100
70
55
35
25
15
Streptavidin-HRP
35
25
Ponceau
E
wtHEK293T
HEK293TRS_BcnK
IN
FT
EL
0.5 mM BcnK
Biotin-Tet
kDa
250
130
100
70
55
35
25
15
Streptavidin-HRP
35
25
Ponceau

Supplementary Figure 3: (A) Proteome-wide amber suppression in mESCs with a stable expanded genetic code. Stable R26^{RS_BcnK} and wt mESCs were cultured for 42 h with 0.5 mM BcnK. Proteins that incorporated BcnK were labeled in cell culture for 30 min with 1 µM fluorophore-tetrazine (SiR-Tet) conjugate. In-gel fluorescence of whole cell lysates was detected at 633 nm. Coomassie staining is presented as loading control. **(B)** Amber suppressed proteome in stable R26^{RS_BcnK} mESCs can be labeled with biotin-tetrazine (Biotin-Tet). R26^{MIN}, R26^{RS_BcnK}, and R26^{RS_BcnK}/PB^{sfGFPN150*} mESCs were cultured for 68 h with 0.5 mM BcnK. Whole cell lysates were labeled overnight at 4°C with 2.5 nmol Biotin-Tet conjugate per 1 mg protein input. Samples were subjected to Western blotting and Ponceau S staining as loading control. Biotinylated proteins were detected using a streptavidin-HRP conjugate. Amber suppressed sfGFP^{N150*} (*) is indicated. **(C)** Schematic workflow of stochastic orthogonal recoding of translation with enrichment (SORT-E) adapted from Elliott et al. (1) to identify endogenous proteins that are amber suppressed (amberome). HEK293T and mESC lines stably expressing PylRS_BcnK (HEK293T^{RS_BcnK} or R26^{RS_BcnK}) and respective control cell lines were cultured for 3 days with BcnK. After wash-out of excess free BcnK for 4 h, cell pellets were split for (i) full proteome analysis and (ii) streptavidin pulldown after overnight labeling of RIPA lysates with Biotin-Tet. Samples were finally analyzed by mass spectrometry (LC-MS/MS). **(D+E)** Quality control gel of selective streptavidin pulldown (replicate 1) of amber suppressed endogenous proteins for analysis by LC-MS/MS (SORT-E approach). Stable R26^{RS_BcnK} and R26^{MIN} mESCs (D) or HEK293T^{RS_BcnK} and wtHEK293T (E) cell lines were cultured for 66 h in the presence of 0.5 mM BcnK, amber suppressed proteins in whole cell lysates covalently labeled overnight at 4°C with 2.5 nmol Biotin-Tet conjugate per 1 mg protein input, and biotinylated proteins captured by streptavidin pulldown. Input (IN), flow-through (FT), and eluate (EL) samples were subjected to Western blotting using a streptavidin-HRP conjugate and Ponceau S staining as loading control.

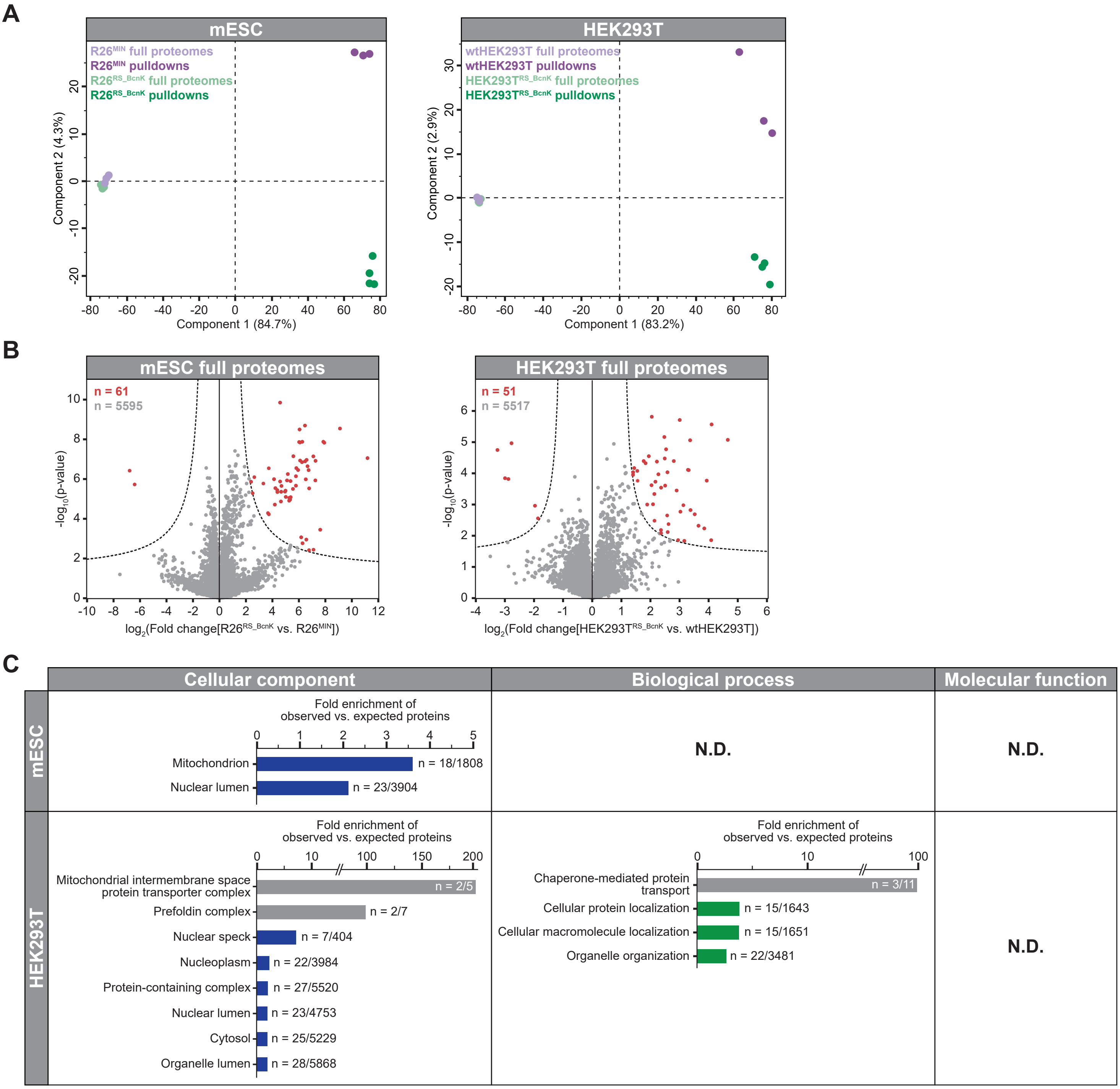
A
mESC
R26MIN full proteomes
R26MIN pulldowns
R26RS_BcnK full proteomes
R26RS_BcnK pulldowns
Component 2 (4.3%)
Component 1 (84.7%)
HEK293T
wtHEK293T full proteomes
wtHEK293T pulldowns
HEK293TRS_BcnK full proteomes
HEK293TRS_BcnK pulldowns
Component 2 (2.9%)
Component 1 (83.2%)
B
mESC full proteomes
n = 61
n = 5595
-log10(p-value)
log2(Fold change[R26RS_BcnK vs. R26MIN])
HEK293T full proteomes
n = 51
n = 5517
-log10(p-value)
log2(Fold change[HEK293TRS_BcnK vs. wtHEK293T])
C
Cellular component
Biological process
Molecular function
mESC
Fold enrichment of observed vs. expected proteins
Mitochondrion
n = 18/1808
Nuclear lumen
n = 23/3904
N.D.
N.D.
HEK293T
Fold enrichment of observed vs. expected proteins
Mitochondrial intermembrane space protein transporter complex
n = 2/5
Prefoldin complex
n = 2/7
Nuclear speck
n = 7/404
Nucleoplasm
n = 22/3984
Protein-containing complex
n = 27/5520
Nuclear lumen
n = 23/4753
Cytosol
n = 25/5229
Organelle lumen
n = 28/5868
Fold enrichment of observed vs. expected proteins
Chaperone-mediated protein transport
n = 3/11
Cellular protein localization
n = 15/1643
Cellular macromolecule localization
n = 15/1651
Organelle organization
n = 22/3481
N.D.

Supplementary Figure 4: (A-C) The global proteome in R26^{RS_BcnK} mESCs and HEK293T^{RS_BcnK} is negligibly altered with no specific cellular response upon amber suppression. **(A)** Principal component analyses (PCA) of full proteome and pulldown LC-MS/MS data from mESC and HEK293T samples. **(B)** Volcano plots depicting fold change and significance between full proteomes from mESC and HEK293T samples (PylRS_BcnK vs. respective control cell line). The number (n) of unchanged (grey) and significantly changed (red) proteins is indicated. **(C)** Gene ontology (GO) enrichment analysis of significantly changed proteins from (B) using the PANTHER classification system. The number (n) of detected and total proteins within each GO term ($p < 0.001$, see also Sup. Data 3 for details) are indicated. Note that the GO terms "mitochondrial intermembrane space protein transporter complex", "prefoldin complex", and "chaperone-mediated protein transport" (grey) only consist of relatively few total proteins and therefore can be considered as non-significant. All other GO terms only represent general categories. N.D. = not detected.

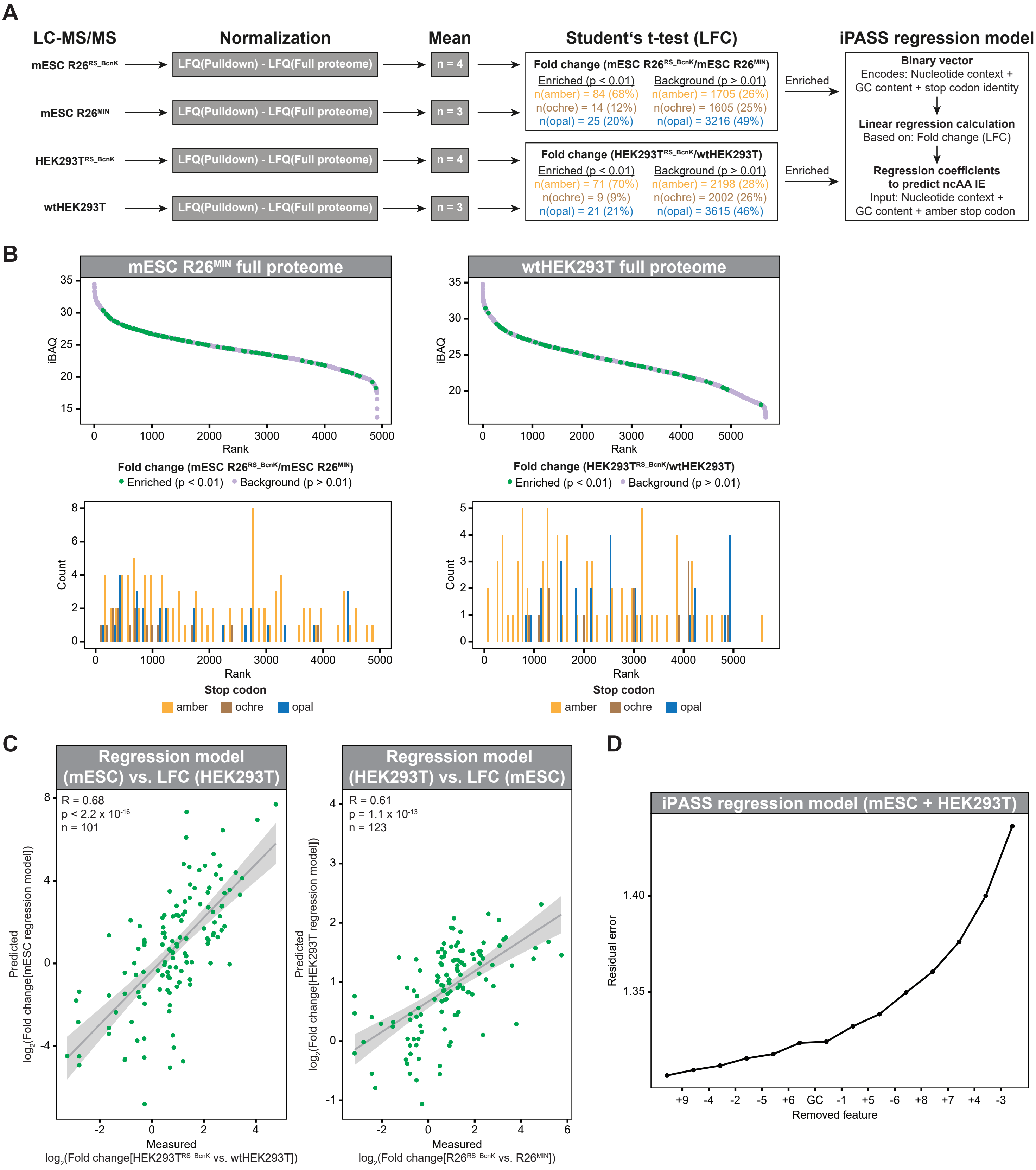
A
LC-MS/MS
Normalization
Mean
Student's t-test (LFC)
iPASS regression model
mESC R26RS_BcnK
mESC R26MIN
HEK293TRS_BcnK
wtHEK293T
LFQ(Pulldown) - LFQ(Full proteome)
n = 4
n = 3
Fold change (mESC R26RS_BcnK/mESC R26MIN)
Enriched (p < 0.01)
n(amber) = 84 (68%)
n(ochre) = 14 (12%)
n(opal) = 25 (20%)
Background (p > 0.01)
n(amber) = 1705 (26%)
n(ochre) = 1605 (25%)
n(opal) = 3216 (49%)
Fold change (HEK293TRS_BcnK/wtHEK293T)
Enriched (p < 0.01)
n(amber) = 71 (70%)
n(ochre) = 9 (9%)
n(opal) = 21 (21%)
Background (p > 0.01)
n(amber) = 2198 (28%)
n(ochre) = 2002 (26%)
n(opal) = 3615 (46%)
Enriched
Binary vector
Encodes: Nucleotide context + GC content + stop codon identity
Linear regression calculation
Based on: Fold change (LFC)
Regression coefficients to predict ncAA IE
Input: Nucleotide context + GC content + amber stop codon
B
mESC R26MIN full proteome
wtHEK293T full proteome
iBAQ
Rank
Fold change (mESC R26RS_BcnK/mESC R26MIN)
Fold change (HEK293TRS_BcnK/wtHEK293T)
Enriched (p < 0.01)
Background (p > 0.01)
Count
Stop codon
amber
ochre
opal
C
Regression model (mESC) vs. LFC (HEK293T)
R = 0.68
p < 2.2 x 10-16
n = 101
Predicted log2(Fold change[mESC regression model])
Measured log2(Fold change[HEK293TRS_BcnK vs. wtHEK293T])
Regression model (HEK293T) vs. LFC (mESC)
R = 0.61
p = 1.1 x 10-13
n = 123
Predicted log2(Fold change[HEK293T regression model])
Measured log2(Fold change[R26RS_BcnK vs. R26MIN])
D
iPASS regression model (mESC + HEK293T)
Residual error
Removed feature
+9 -4 -2 -5 +6 GC -1 +5 -6 +8 +7 +4 -3

Supplementary Figure 5: (A) Identification of suppressed endogenous amber stop codons in R26^{RS_BcnK} mESC and HEK293T^{RS_BcnK} cells in three steps to compute a linear regression model: (i) streptavidin pulldowns were normalized to their respective full proteome samples by subtraction of label free quantification (LFQ) values (imputation of missing partner value) to account for relative amber suppression levels (e.g. weak amber suppression of highly expressed protein vs. strong amber suppression of weakly expressed protein) and variations in protein expression between replicates and cell lines; (ii) mean values of these normalized fold changes for PylRS_BcnK (quadruplicates) and control (triplicates) cell lines were calculated; (iii) calculation of $\log_2$(fold change) (LFC): significantly enriched ($p < 0.01$) proteins upon amber suppression with BcnK were identified by Student's t-test between mESC R26^{RS_BcnK} and R26^{MIN} or HEK293T^{RS_BcnK} and wtHEK293T normalized mean fold change values. For each stop codon (amber, ochre, opal), the number (n) and relative fraction (%) of identified proteins within the enriched and background fraction is indicated. Sequence contexts around the termination codons (up to 6 bp up- and downstream) of significantly enriched proteins were subsequently extracted to calculate a linear regression model. To calculate regression coefficients, nucleotide context, stop codon identity, and GC content were encoded in a binary vector and correlated with their experimental LFC values. **(B-D)** Samples were processed and analyzed as described in Sup. Fig. 3C and 5A. **(B)** Significantly enriched proteins identified by SORT-E are enriched independent of their cellular abundance. Upper panel: Full proteomes of mESC R26^{MIN} (left) and wtHEK293T (right) are plotted according to their rank and respective Intensity Based Absolute Quantification (iBAQ) values with significantly ($p < 0.01$) enriched proteins (see Sup. Fig. 5A) being highlighted (green). Lower panel: Histograms (bin width = 100) of ranks of significantly enriched proteins from above. **(C)** Linear regression models reliably predict LFC as a readout of ncAA incorporation efficiency in mammalian cells. Experimentally determined LFC values from HEK293T cells (left) and mESCs (right) were correlated with LFC values predicted by mESC (left) and HEK293T (right) specific regression models, respectively. Pearson correlation coefficient (R), p-value (p), and number (n) of significantly enriched stop codon sequence contexts (see Sup. Fig. 5A) that were used as input for prediction are indicated. The 95% confidence interval of the regression line is marked. **(D)** Successive elimination of features encoded within the iPASS model (combined linear regression analysis of significantly enriched stop codon sequence contexts from both mESCs and HEK293T cells) gradually decreases accuracy of the linear regression and hence prediction of ncAA incorporation efficiency. Features with the smallest contribution to the residual error were successively removed from the regression analysis (from left to right). The x-axis indicates step-wise removed features (nucleotide positions or GC content).

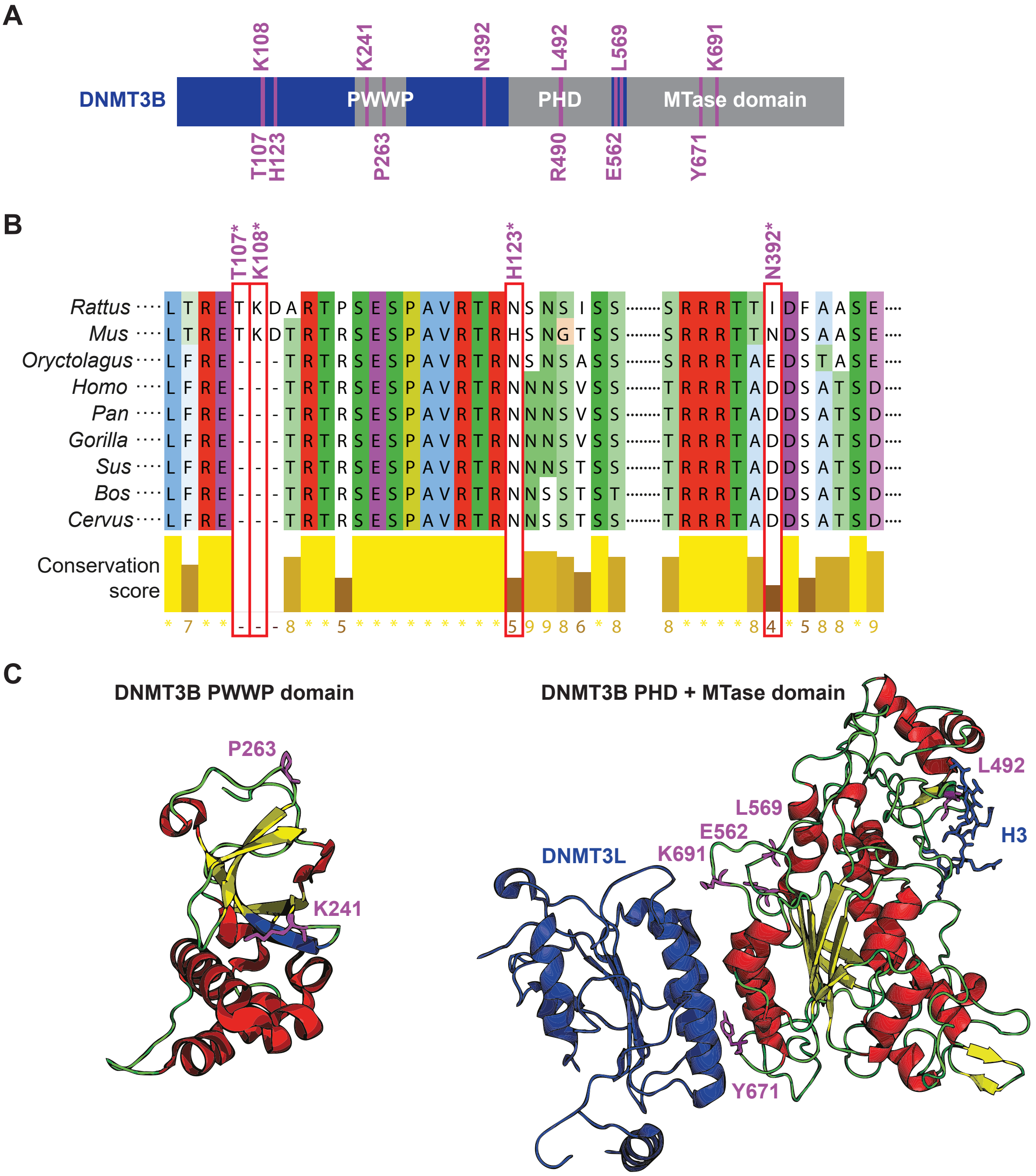
A
DNMT3B
PWWP
PHD
MTase domain
K108
K241
N392
L492
L569
K691
T107
H123
P263
R490
E562
Y671
B
T107*
K108*
H123*
N392*
Rattus LTRETKDARTPSESPAVRTRNSNSISS SRRRTTIDFAASE
Mus LTRETKDTRTRSESPAVRTRHSNGTSS SRRRTTNDSAASE
Oryctolagus LFRE---TRTRSESPAVRTRNSNSASS SRRRTAEDSTASE
Homo LFRE---TRTRSESPAVRTRNNNSVSS TRRRTADDSATSD
Pan LFRE---TRTRSESPAVRTRNNNSVSS TRRRTADDSATSD
Gorilla LFRE---TRTRSESPAVRTRNNNSVSS TRRRTADDSATSD
Sus LFRE---TRTRSESPAVRTRNNNSTSS TRRRTADDSATSD
Bos LFRE---TRTRSESPAVRTRNNSSTST TRRRTADDSATSD
Cervus LFRE---TRTRSESPAVRTRNNSSTSS TRRRTADDSATSD
Conservation score
* 7 * * - - - 8 * * 5 * * * * * * * * * 5 9 9 8 6 * 8 8 * * * * 8 4 * 5 8 8 * 9
C
DNMT3B PWWP domain
DNMT3B PHD + MTase domain
P263
K241
DNMT3L
K691
E562
L569
L492
H3
Y671

Supplementary Figure 6: Selection of 11 ncAA incorporation sites in DNMT3B. **(A)** Schematic representation of *Mus musculus* DNMT3B. The PWWP, PHD, and methyltransferase (MTase) domain as well as position of amber stop codons are indicated. **(B)** Multiple sequence alignment of *M. musculus* DNMT3B amino acids 103-129 and 386-398 (NCBI ref. seq. NP_001258673.1) with DNMT3B from indicated species: *Rattus norvegicus* (NCBI ref. seq. NP_001003959.1), *Oryctolagus cuniculus* (NCBI ref. seq. XP_017197180.1), *Homo sapiens* (NCBI ref. seq. NP_008823.1), *Pan troglodytes* (NCBI ref. seq. XP_016793181.1), *Gorilla gorilla gorilla* (NCBI ref. seq. XP_004062033.1), *Sus scrofa* (NCBI ref. seq. NP_001335829.1), *Bos taurus* (NCBI ref. seq. NP_861529.2), *Cervus elaphus hippelaphus* (NCBI ref. seq. OWK03372.1). Amino acids with a low conservation score and selected for ncAA incorporation are marked with red boxes. **(C)** Crystal structure of *M. musculus* DNMT3B PWWP domain (PDB ID: 1KHC (2); PWWP motif colored in blue) and *M. musculus* DNMT3B PHD and MTase domain in complex with DNMT3L and histone H3 (modelled with SWISS-MODEL workspace (3); model based on PDB ID: 4U7T (4)). Selected amber stop codon positions are indicated in purple.

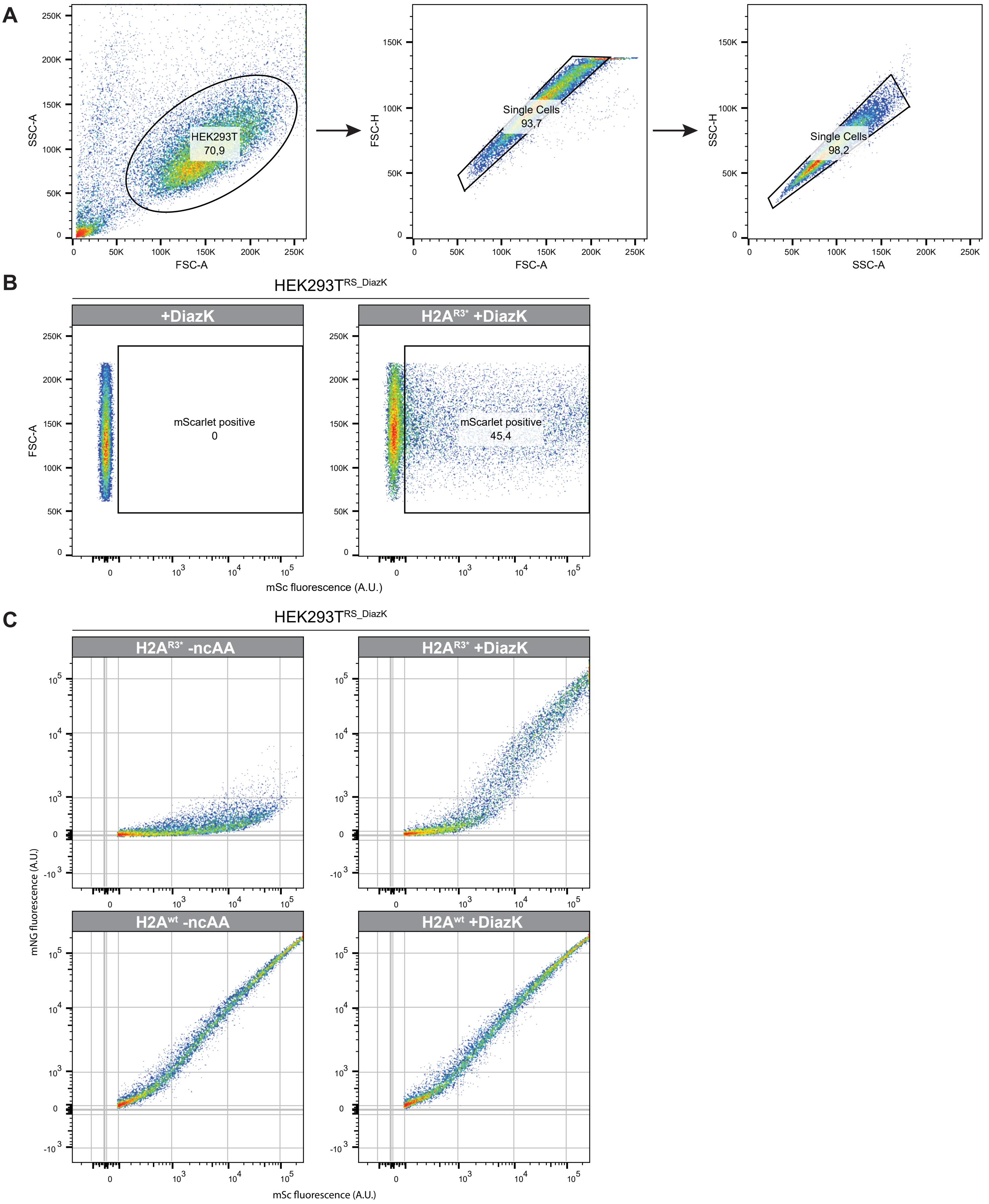

A
SSC-A
FSC-A
HEK293T
70,9
FSC-H
FSC-A
Single Cells
93,7
SSC-H
SSC-A
Single Cells
98,2
B
HEK293TRS_DiazK
+DiazK
H2AR3* +DiazK
FSC-A
mScarlet positive
0
mScarlet positive
45,4
mSc fluorescence (A.U.)
C
HEK293TRS_DiazK
H2AR3* -ncAA
H2AR3* +DiazK
H2Awt -ncAA
H2Awt +DiazK
mNG fluorescence (A.U.)
mSc fluorescence (A.U.)

Supplementary Figure 7: Representative flow-cytometry data of HEK293T^{RS}/GOI*. **(A+B)** Illustration of gating strategy to analyze expression of the mSc/mNG fluorescent reporter by flow cytometry after transient transfection of HEK293T^{RS} polyclonal pools. Here exemplified by HEK293T^{RS_DiazK} transiently transfected with the mSc/mNG fluorescent reporter harboring H2A^{R3*} (HEK293T^{RS_DiazK}/H2A^{R3*}) and cultured for 24 h with 0.5 mM DiazK. Debris (FSC-A/SSC-A) and doublets (FSC-A/FSC-H and SSC-A/SSC-H) were excluded (A) and ca. 10,000 mSc positive single cells (B, right panel) per replicate analyzed (mSc positive single cell counts per replicate are listed in Sup. Data 2). Untransfected HEK293T^{RS_DiazK} were used as mSc negative control (B, left panel). Numbers indicate percentage of events within gates. Fluorescence intensities are indicated in arbitrary units (A.U.). Abbreviations: Forward scatter area (FSC-A), forward scatter height (FSC-H), side scatter area (SSC-A), side scatter height (SSC-H). **(C)** Representative dot plots depicting mSc and mNG fluorescence intensities to assess incorporation efficiencies of ncAAs across amber stop codon positions within a *GOI** in HEK293T^{RS} polyclonal pools according to Fig. 3B. Here exemplified by HEK293T^{RS_DiazK}/H2A^{R3*} or HEK293T^{RS_DiazK}/H2A^{wt} cultured for 24 h in the presence or absence of 0.5 mM DiazK. Ca. 10,000 mSc positive single cells per condition were analyzed to calculate mean fluorescence intensities (MFIs) of mSc and mNG. Fluorescence intensities are indicated in arbitrary units (A.U.).

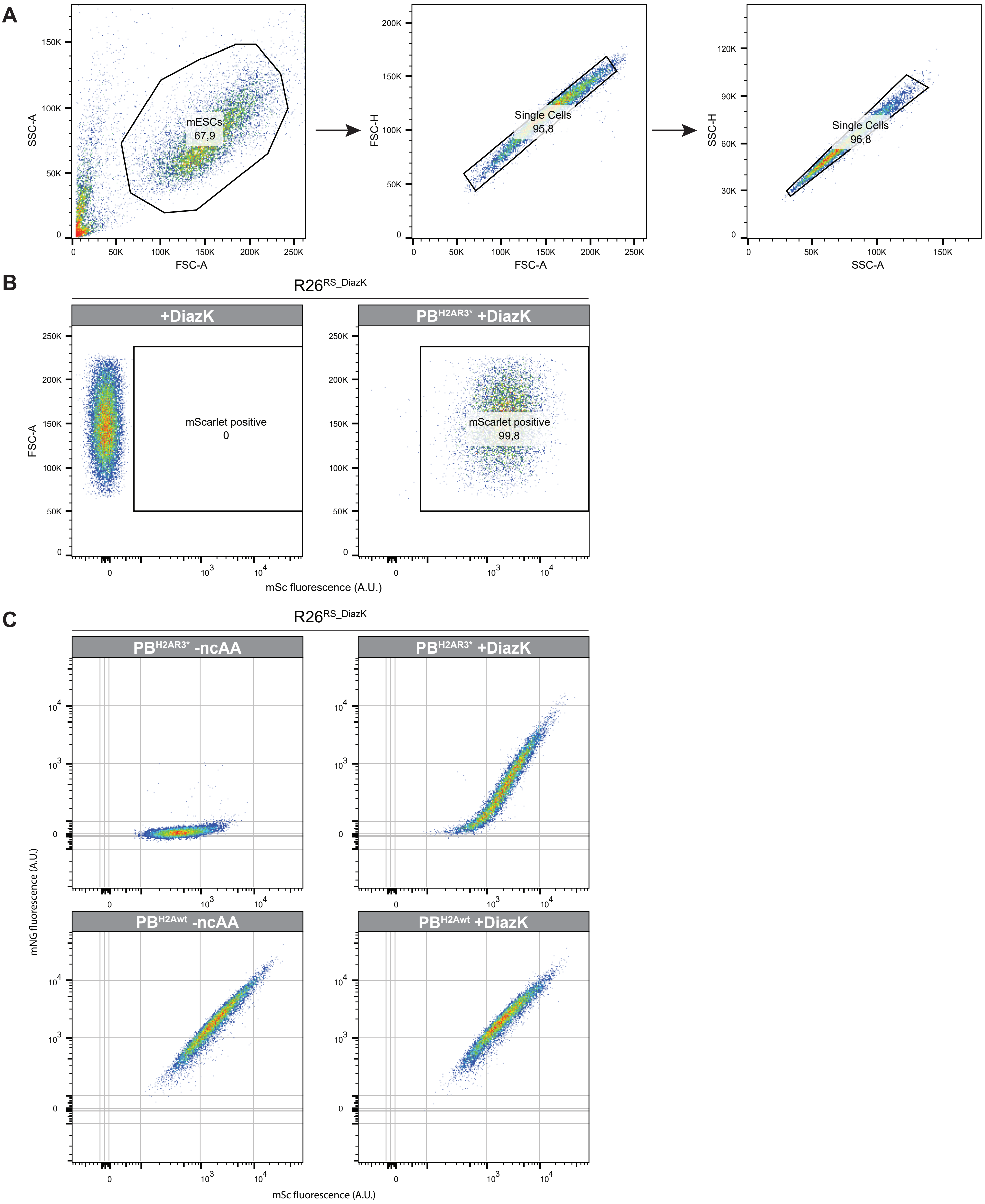

A
mESCs
67,9
SSC-A
FSC-A
Single Cells
95,8
FSC-H
FSC-A
Single Cells
96,8
SSC-H
SSC-A
B
R26RS_DiazK
+DiazK
PBH2AR3* +DiazK
mScarlet positive
0
mScarlet positive
99,8
FSC-A
mSc fluorescence (A.U.)
C
R26RS_DiazK
PBH2AR3* -ncAA
PBH2AR3* +DiazK
PBH2Awt -ncAA
PBH2Awt +DiazK
mNG fluorescence (A.U.)
mSc fluorescence (A.U.)

Supplementary Figure 8: Representative flow-cytometry data of R26^{RS}/PB^{GOI*} mESCs. **(A+B)** Illustration of gating strategy to analyze expression of the mSc/mNG fluorescent reporter by flow cytometry after PB mediated genomic integration into R26^{RS} mESC clones. Here exemplified by mESC clone R26^{RS_DiazK} stably expressing the mSc/mNG fluorescent reporter harboring H2A^{R3*} (R26^{RS_DiazK}/PB^{H2AR3*}) and cultured for 24 h with 0.5 mM DiazK. Debris (FSC-A/SSC-A) and doublets (FSC-A/FSC-H and SSC-A/SSC-H) were excluded (A) and ca. 10,000 mSc positive single cells (B, right panel) per replicate analyzed (mSc positive single cell counts per replicate are listed in Sup. Data 2). The R26^{RS_DiazK} entry clone for PB transposition is used as mSc negative control (B, left panel). Numbers indicate percentage of events within gates. Fluorescence intensities are indicated in arbitrary units (A.U.). Abbreviations: Forward scatter area (FSC-A), forward scatter height (FSC-H), side scatter area (SSC-A), side scatter height (SSC-H). **(C)** Representative dot plots depicting mSc and mNG fluorescence intensities to assess incorporation efficiencies of ncAAs across amber stop codon positions within a *GOI** in R26^{RS} mESC clones according to Fig. 3B. Here exemplified by stable R26^{RS_DiazK}/PB^{H2AR3*} or R26^{RS_DiazK}/PB^{H2Awt} cultured for 24 h in the presence or absence of 0.5 mM DiazK. Ca. 10,000 mSc positive single cells per condition were analyzed to calculate mean fluorescence intensities (MFIs) of mSc and mNG. Fluorescence intensities are indicated in arbitrary units (A.U.).

A

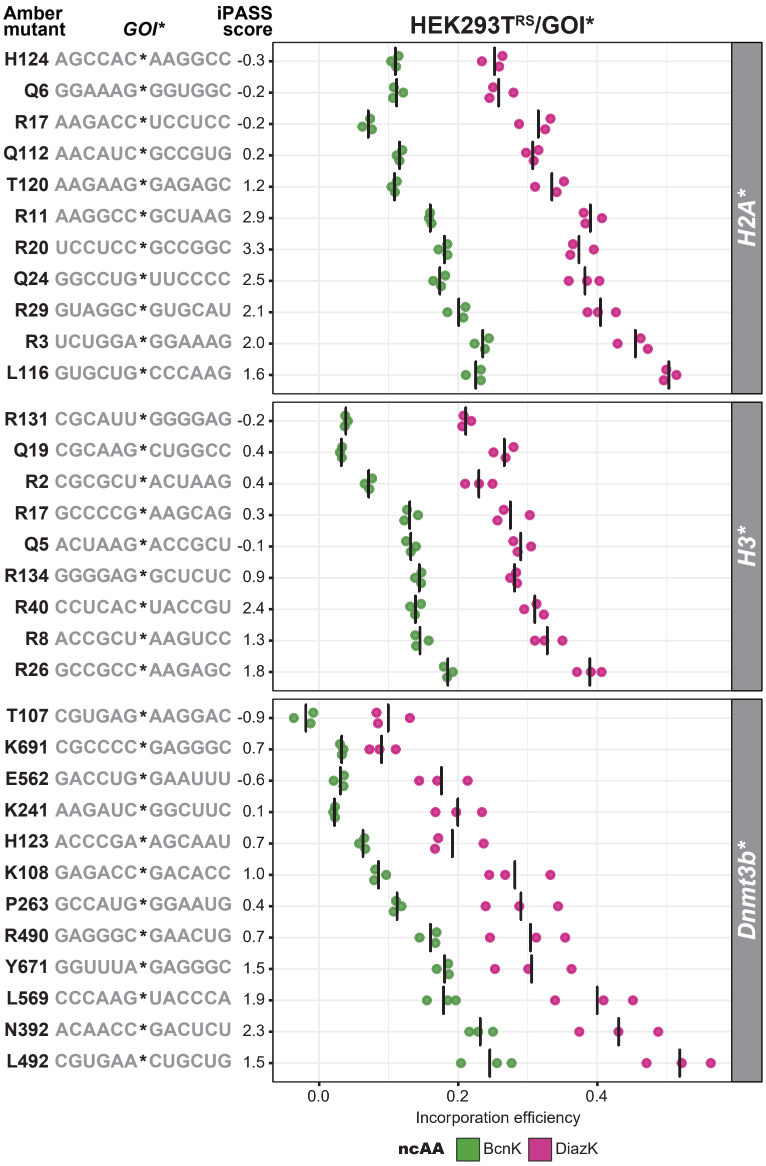

B

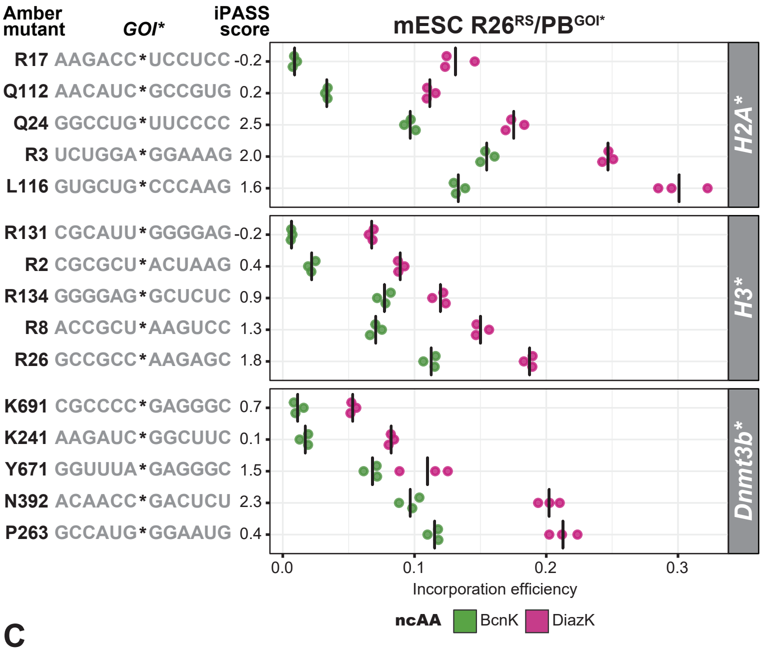

C

ncAA	Cell line	GOI*	n	Mean IE (min)	Mean IE (max)	Fold change
BcnK	HEK293T^{RS_BcnK}	*H2A**	11	0.07	0.24	4.2
		*H3**	9	0.03	0.19	7.6
		*Dnmt3b**	12	0.01	0.25	33.0
	mESC R26^{RS_BcnK}	*H2A**	5	0.01	0.15	17.5
		*H3**	5	0.01	0.11	12.2
		*Dnmt3b**	5	0.01	0.12	13.5
DiazK	HEK293T^{RS_DiazK}	*H2A**	11	0.25	0.50	3.0
		*H3**	9	0.21	0.39	2.4
		*Dnmt3b**	12	0.09	0.52	11.0
	mESC R26^{RS_DiazK}	*H2A**	5	0.11	0.30	3.5
		*H3**	5	0.07	0.19	3.1
		*Dnmt3b**	5	0.05	0.21	5.1

Supplementary Figure 9: (A+B) Position dependent variability of ncAA incorporation efficiency in HEK293T cells stably expressing the respective PylRS and transiently transfected with the mSc/mNG fluorescent reporter ($HEK293T^{RS}$/GOI*; A) or mESCs stably expressing both PylRS and mSc/mNG fluorescent reporter (mESC $R26^{RS}/PB^{GOI*}$; B). Incorporation efficiency for BcnK and DiazK at each *GOI** site (*H2A**, *H3**, or *Dnmt3b** amber mutants) was measured for n = 3 biological replicates and calculated according to Fig. 3B. Vertical black lines represent mean values. For each *GOI** the nucleotide sequence +/- 6 bp flanking the amber stop codon (*) and its respective iPASS score are presented. Per replicate, mNG and mSc mean fluorescence intensities from ca. 10,000 mSc positive single cells were acquired by flow-cytometry 24 h after addition of 0.5 mM ncAA. mSc positive single cell counts per replicate are listed in Sup. Data 2. **(C)** Table summarizing for each ncAA, cell line, and *GOI** the number (n) of analyzed mSc/mNG fluorescent reporters harboring different *GOI**, the minimal and maximal mean incorporation efficiency (IE) across all *GOI** analyzed, and their respective fold change. According to Smith and Yarus (5), the fold change between incorporation efficiencies at two different amber stop codon sequence contexts is calculated as {IE(max)x[1-IE(min)]}/{IE(min)x[1-IE(max)]}. Note that the BcnK mean incorporation efficiency for $HEK293T^{RS\_BcnK}/Dnmt3b^{T107*}$ was negative (see Sup. Fig. 9A) but set to 0.01 as minimal value to allow calculation of the respective fold change.

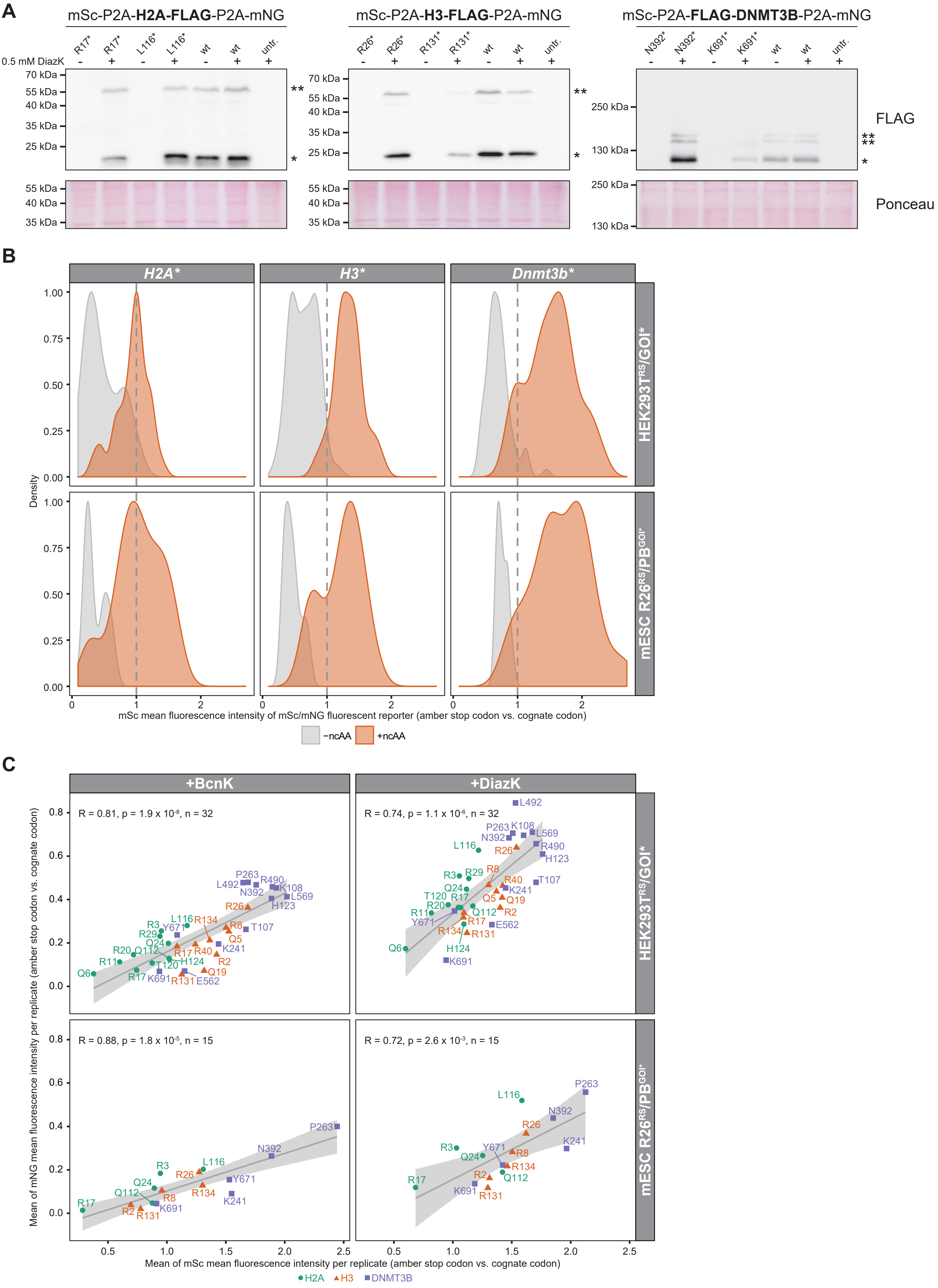

A
mSc-P2A-H2A-FLAG-P2A-mNG
mSc-P2A-H3-FLAG-P2A-mNG
mSc-P2A-FLAG-DNMT3B-P2A-mNG
0.5 mM DiazK
R17* R17* L116* L116* wt wt untr.
R26* R26* R131* R131* wt wt untr.
N392* N392* K691* K691* wt wt untr.
70 kDa
55 kDa
40 kDa
35 kDa
25 kDa
250 kDa
130 kDa
FLAG
Ponceau
B
H2A*
H3*
Dnmt3b*
HEK293T^RS/GOI*
mESC R26^RS/PB^GOI*
Density
mSc mean fluorescence intensity of mSc/mNG fluorescent reporter (amber stop codon vs. cognate codon)
−ncAA
+ncAA
C
+BcnK
+DiazK
R = 0.81, p = 1.9 x 10⁻⁸, n = 32
R = 0.74, p = 1.1 x 10⁻⁶, n = 32
R = 0.88, p = 1.8 x 10⁻⁵, n = 15
R = 0.72, p = 2.6 x 10⁻³, n = 15
Mean of mNG mean fluorescence intensity per replicate (amber stop codon vs. cognate codon)
Mean of mSc mean fluorescence intensity per replicate (amber stop codon vs. cognate codon)
H2A
H3
DNMT3B

Supplementary Figure 10: (A) Incorporation efficiency measured by flow-cytometry is reflected on the protein level. Stable HEK293T^{RS_DiazK} were transfected with the indicated amber mutant reporter construct and cultured for 24 h with 0.5 mM DiazK. Analyzed amber stop codon positions are exemplary for low (H2A^{R17*}, H3^{R131*}, DNMT3B^{K691*}) and high (H2A^{L116*}, H3^{R26*}, DNMT3B^{N392*}) incorporation efficiencies. Whole cell lysates were subjected to immunoblotting using FLAG antibody and Ponceau S staining as loading control. Fully cleaved (*) and peptides with uncleaved P2A (**) are indicated. Untransfected control sample (untr.). **(B)** Expression of amber mutants from reporter construct is disproportionately increased upon ncAA addition relative to wt. mSc mean fluorescence intensity (MFI) distribution for all *H2A**, *H3**, and *Dnmt3b** amber mutants (*GOI**) analyzed in this study (see Fig. 3A) relative to the respective wild-type *H2A*, *H3*, and *Dnmt3b* construct (GOI^{wt}) in the absence (-ncAA) or presence (+ncAA) of an ncAA (BcnK as well as DiazK are plotted together). mSc MFI +ncAA or -ncAA of each analyzed amber mutant (n = 3 biological replicates) was normalized to the respective mSc MFI +ncAA or -ncAA of the wild-type construct for HEK293T cells stably expressing the respective PylRS and transiently transfected with the mSc/mNG fluorescent reporter (HEK293T^{RS}/GOI*) or mESCs stably expressing both PylRS and mSc/mNG reporter (mESC R26^{RS}/PB^{GOI*}). The dashed line indicates mSc MFI(*GOI**) = mSc MFI(GOI^{wt}). Per replicate, mSc MFI from ca. 10,000 mSc positive single cells was acquired by flow-cytometry 24 h after transfer to 0.5 mM ncAA containing medium. HEK293T^{RS}/GOI*: n = 22 (*H2A**), n = 18 (*H3**), n = 24 (*Dnmt3b**). mESC R26^{RS}/PB^{GOI*}: n = 10 (*H2A**, *H3**, *Dnmt3b**). **(C)** Disproportionate increase in mSc MFI relative to GOI^{wt} is correlated with amber suppression in HEK293T^{RS} cells and R26^{RS} mESC clones. Mean (n = 3 biological replicates) mSc and mNG MFIs of HEK293T^{RS}/GOI* or mESC R26^{RS}/PB^{GOI*} were calculated for each position and normalized to the respective GOI^{wt}. Pearson correlation coefficient (R), p-value (p), and number (n) of analyzed mSc/mNG fluorescent reporters harboring different *GOI** are indicated. The 95% confidence interval of the regression line is marked.

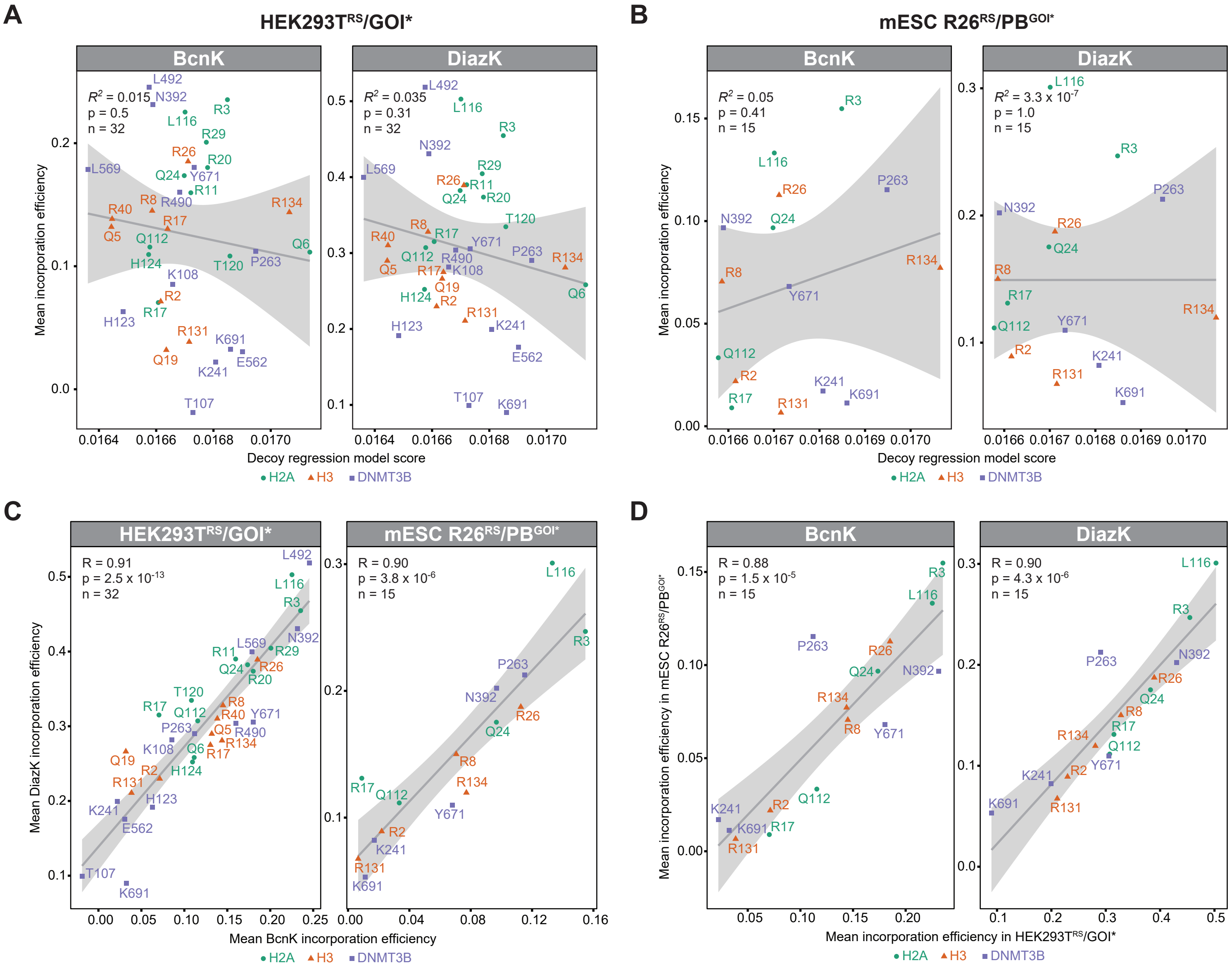

A
HEK293T^{RS}/GOI*
BcnK
R^2 = 0.015
p = 0.5
n = 32
DiazK
R^2 = 0.035
p = 0.31
n = 32
Mean incorporation efficiency
Decoy regression model score
H2A
H3
DNMT3B
B
mESC R26^{RS}/PB^{GOI*}
BcnK
R^2 = 0.05
p = 0.41
n = 15
DiazK
R^2 = 3.3 x 10^{-7}
p = 1.0
n = 15
Mean incorporation efficiency
Decoy regression model score
H2A
H3
DNMT3B
C
HEK293T^{RS}/GOI*
R = 0.91
p = 2.5 x 10^{-13}
n = 32
mESC R26^{RS}/PB^{GOI*}
R = 0.90
p = 3.8 x 10^{-6}
n = 15
Mean DiazK incorporation efficiency
Mean BcnK incorporation efficiency
H2A
H3
DNMT3B
D
BcnK
R = 0.88
p = 1.5 x 10^{-5}
n = 15
DiazK
R = 0.90
p = 4.3 x 10^{-6}
n = 15
Mean incorporation efficiency in mESC R26^{RS}/PB^{GOI*}
Mean incorporation efficiency in HEK293T^{RS}/GOI*
H2A
H3
DNMT3B

Supplementary Figure 11: (A-D) Mean incorporation efficiencies (n = 3 biological replicates) of DiazK and BcnK at each site were calculated for HEK293T^{RS}/GOI* or mESC R26^{RS}/PB^{GOI*} using +ncAA and -ncAA RREs from Fig. 3C (also see Sup. Fig. 9C+D). **(A+B)** A decoy model fails to predict relative ncAA incorporation efficiencies into target sites in mammalian cells. Relative incorporation efficiencies at each target site predicted by a decoy regression model were correlated with experimentally determined mean incorporation efficiencies of DiazK and BcnK using the mNG/mSc fluorescent reporter in HEK293T^{RS}/GOI* (A) or mESC R26^{RS}/PB^{GOI*} (B) lines. To calculate the decoy regression model, combined linear regression analysis of randomly selected stop codon sequence contexts from both mESCs and HEK293T cells was performed: Experimental log_2(fold change) (LFC) values determined by SORT-E (see Sup. Fig. 5A) were randomly re-assigned to all sequence contexts detected and a linear regression model calculated after extracting the respective significantly enriched sequence contexts. Nucleotide context, stop codon identity, and GC content were encoded in a binary vector and correlated with their randomly associated LFC value to calculate regression coefficients. Stop codons were encoded as a single nucleotide position. Coefficient of determination (R^2), p-value (p), and number (n) of analyzed mSc/mNG fluorescent reporters harboring different *GOI** are indicated. The 95% confidence interval of the regression line is marked. **(C+D)** Relative incorporation efficiency in *GOI** is in general independent of ncAA (C) and cell line (D) identity. Experimentally determined mean incorporation efficiencies were correlated between ncAAs (C) or cell lines (D). Pearson correlation coefficient (R), p-value (p), and number (n) of analyzed mSc/mNG fluorescent reporters harboring different *GOI** are indicated. The 95% confidence interval of the regression line is marked.

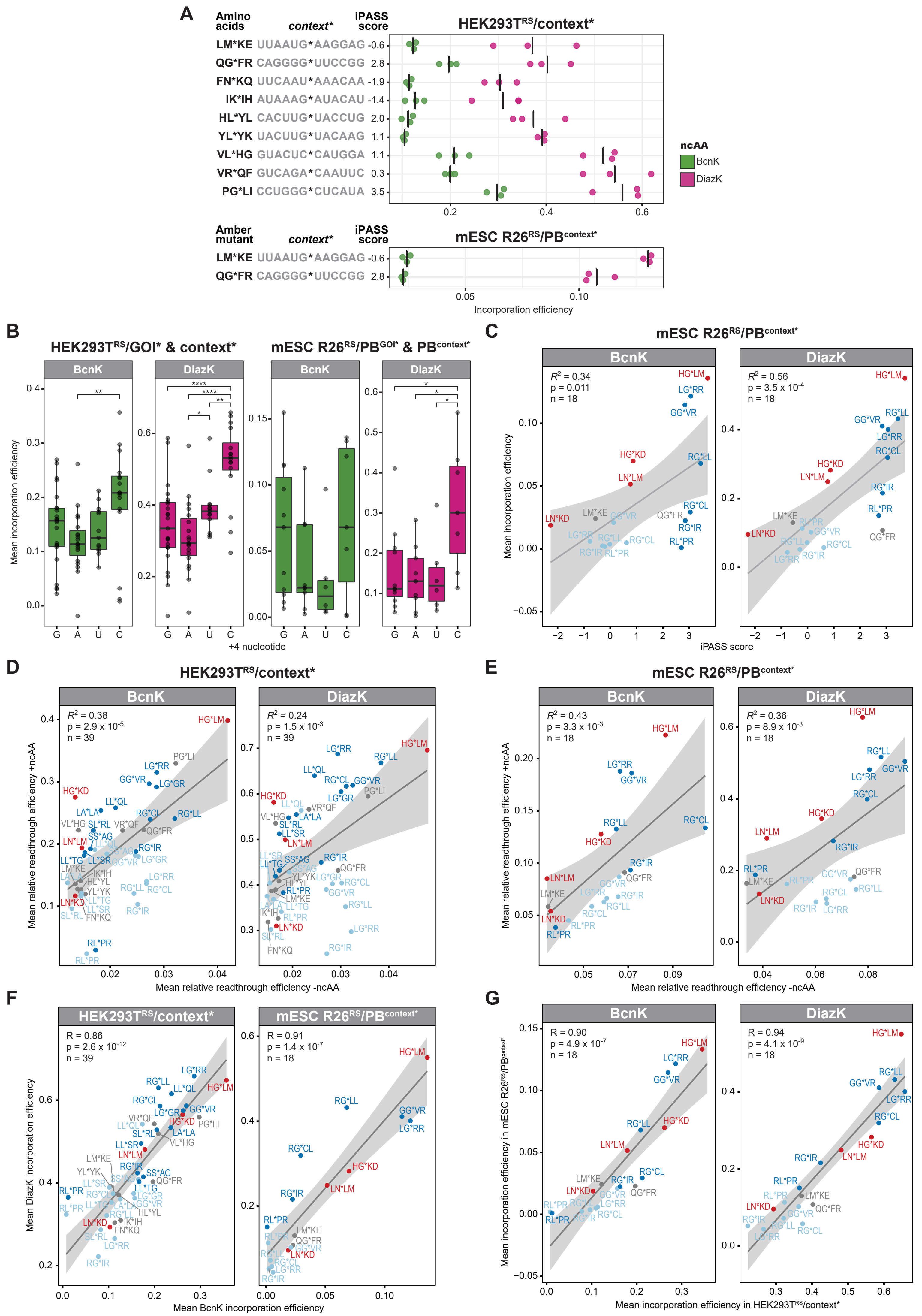

A
Amino acids
context*
iPASS score
HEK293T^RS/context*
LM*KE UUAAUG*AAGGAG -0.6
QG*FR CAGGGG*UUCCGG 2.8
FN*KQ UUCAAU*AAACAA -1.9
IK*IH AUAAAG*AUACAU -1.4
HL*YL CACUUG*UACCUG 2.0
YL*YK UACUUG*UACAAG 1.1
VL*HG GUACUC*CAUGGA 1.1
VR*QF GUCAGA*CAAUUC 0.3
PG*LI CCUGGG*CUCAUA 3.5
ncAA
BcnK
DiazK
Amber mutant
mESC R26^RS/PB^context*
LM*KE UUAAUG*AAGGAG -0.6
QG*FR CAGGGG*UUCCGG 2.8
Incorporation efficiency
B
HEK293T^RS/GOI* & context*
mESC R26^RS/PB^GOI* & PB^context*
BcnK
DiazK
Mean incorporation efficiency
+4 nucleotide
C
mESC R26^RS/PB^context*
R² = 0.34
p = 0.011
n = 18
R² = 0.56
p = 3.5 x 10⁻⁴
n = 18
Mean incorporation efficiency
iPASS score
D
HEK293T^RS/context*
R² = 0.38
p = 2.9 x 10⁻⁵
n = 39
R² = 0.24
p = 1.5 x 10⁻³
n = 39
Mean relative readthrough efficiency +ncAA
E
mESC R26^RS/PB^context*
R² = 0.43
p = 3.3 x 10⁻³
n = 18
R² = 0.36
p = 8.9 x 10⁻³
n = 18
Mean relative readthrough efficiency -ncAA
F
HEK293T^RS/context*
mESC R26^RS/PB^context*
R = 0.86
p = 2.6 x 10⁻¹²
n = 39
R = 0.91
p = 1.4 x 10⁻⁷
n = 18
Mean DiazK incorporation efficiency
Mean BcnK incorporation efficiency
G
R = 0.90
p = 4.9 x 10⁻⁷
n = 18
R = 0.94
p = 4.1 x 10⁻⁹
n = 18
Mean incorporation efficiency in mESC R26^RS/PB^context*
Mean incorporation efficiency in HEK293T^RS/context*

Supplementary Figure 12: (A-G) Efficiencies (n = 3 biological replicates) of BcnK or DiazK incorporation at *context** amber mutants were calculated (according to Fig. 3B) for HEK293T cells stably expressing the respective PylRS and transiently transfected with the *mSc-P2A-context*-P2A-mNG/4xPylT* fluorescent reporter ($HEK293T^{RS}$/context*) or mESCs stably expressing both PylRS and *mSc-P2A-context*-P2A-mNG/4xPylT* fluorescent reporter (mESC $R26^{RS}/PB^{context*}$). Per replicate, mNG and mSc mean fluorescence intensities from 5,000 - 10,000 mSc positive single cells were acquired by flow-cytometry 24 h after addition of 0.5 mM ncAA. mSc positive single cell counts per replicate are listed in Sup. Data 2. **(A)** *Context** target sites additionally analyzed to amber stop codon contexts from Fig. 4B-D. Vertical black lines represent mean values. For each *context** the nucleotide sequence +/- 6 bp flanking the amber stop codon (*) and its respective iPASS score are presented. **(B)** The UAGC tetranucleotide termination signal generally confers higher incorporation efficiencies than UAGG, UAGA, and UAGU. Mean incorporation efficiencies at each analyzed *GOI** and *context** site are grouped according to the +4 nucleotide for DiazK and BcnK in $HEK293T^{RS}$ (n = 23 (+4G), n = 19 (+4A), n = 13 (+4U), n = 16 (+4C)) or mESC $R26^{RS}$ (n = 11 (+4G), n = 9 (+4A), n = 6 (+4U), n = 7 (+4C)). Horizontal black lines within boxes represent median values, boxes indicate the lower and upper quartiles, and whiskers indicate the 1.5 interquartile range. Two-tailed unpaired two-sample Student's t-test: * $p < 0.05$, ** $p < 0.01$, **** $p < 0.0001$, others not significant. **(C-E)** Coefficient of determination (R^2), p-value (p), and number (n) of analyzed mSc/mNG fluorescent reporters harboring different *context** are indicated. The 95% confidence interval of the regression line is marked. Color coding according to Fig. 4B-D (grey: additional *context** from Sup. Fig. 12A). **(C)** The iPASS model reliably predicts relative ncAA incorporation efficiencies at selected *context** mutants in mammalian cells. iPASS scores of each *context** target site were correlated with experimentally determined mean incorporation efficiencies of DiazK or BcnK in mESC $R26^{RS}/PB^{context*}$ lines. **(D+E)** Degree of translational readthrough by near-cognate tRNAs at *context** amber stop codons in $HEK293T^{RS}$/context* cells (D) and $R26^{RS}/PB^{context*}$ mESCs (E) is correlated with their overall suppression by PylT in the presence of BcnK or DiazK. Mean relative readthrough efficiencies (RREs) +ncAA and -ncAA at each *context** site are depicted. **(F+G)** Relative incorporation efficiency in *context** is in general independent of ncAA (F) and cell line (G) identity. Experimentally determined mean incorporation efficiencies were correlated between ncAAs (F) or cell lines (G). Pearson correlation coefficient (R), p-value (p), and number (n) of analyzed mSc/mNG fluorescent reporters harboring different *context** are indicated. The 95% confidence interval of the regression line is marked. Color coding according to Fig. 4B-D (grey: additional *context** from Sup. Fig. 12A).

A

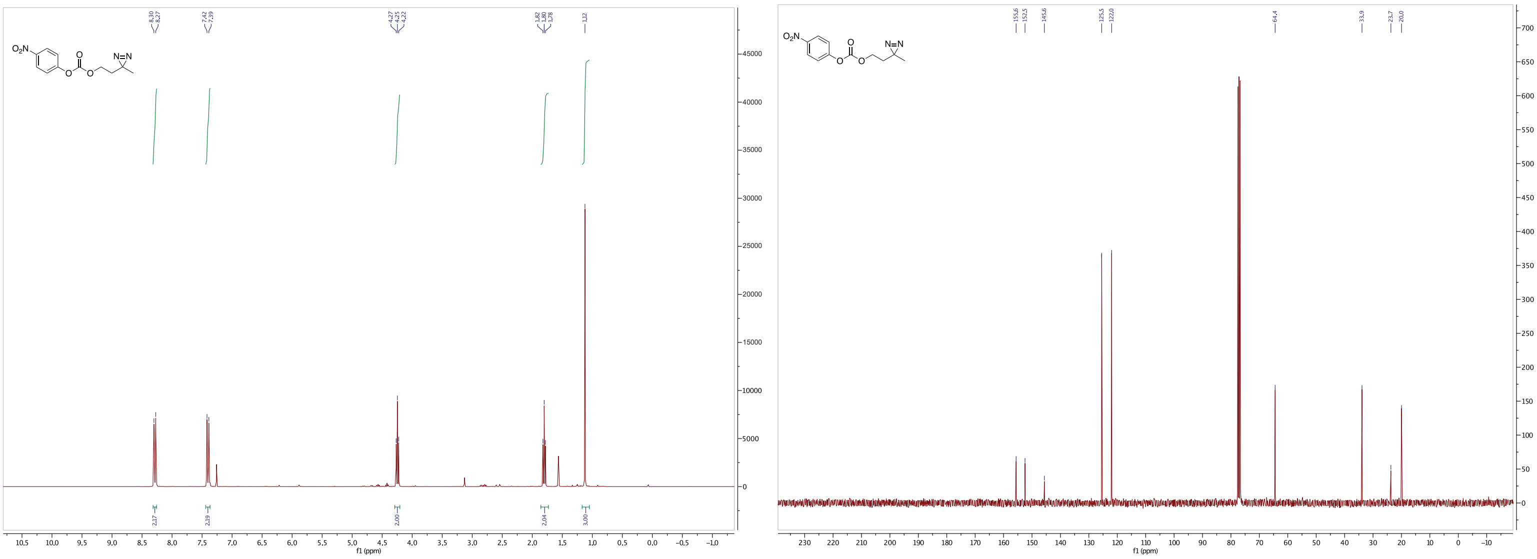

B

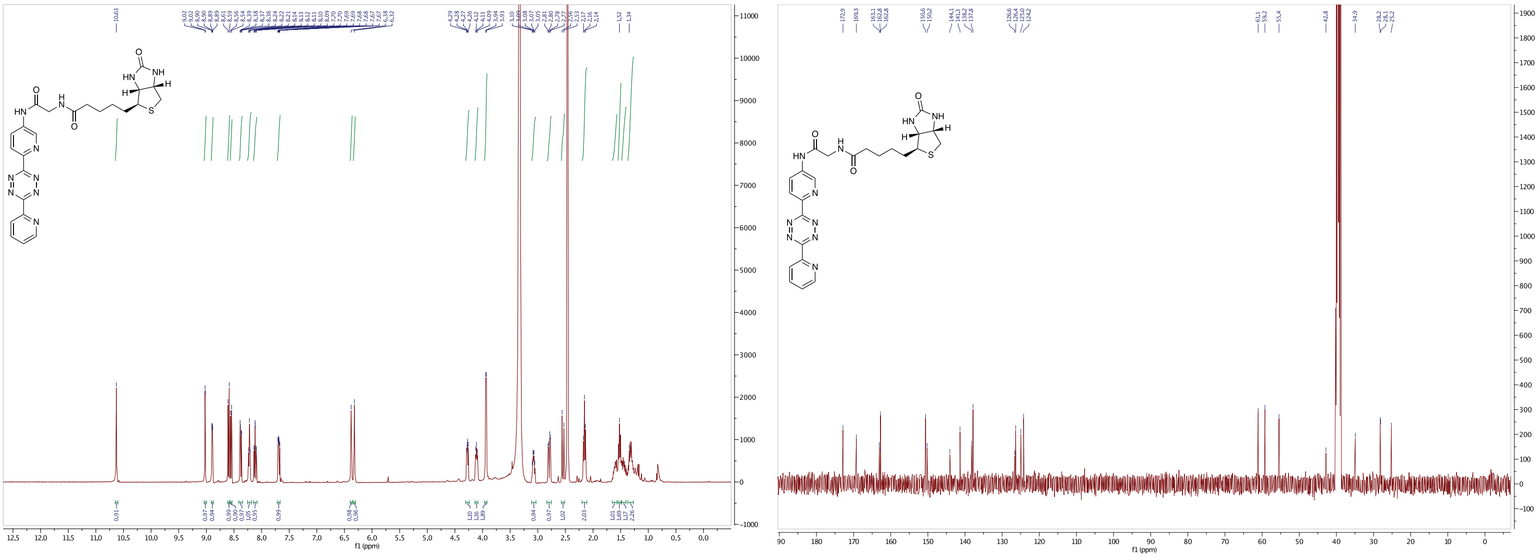

Supplementary Figure 13: (A+B) Nuclear magnetic resonance (NMR) spectra of 2-(3-Methyldiazirin-3-yl)-ethyl (4-nitrophenyl) carbonate (A) or Biotin-tetrazine conjugate (B).

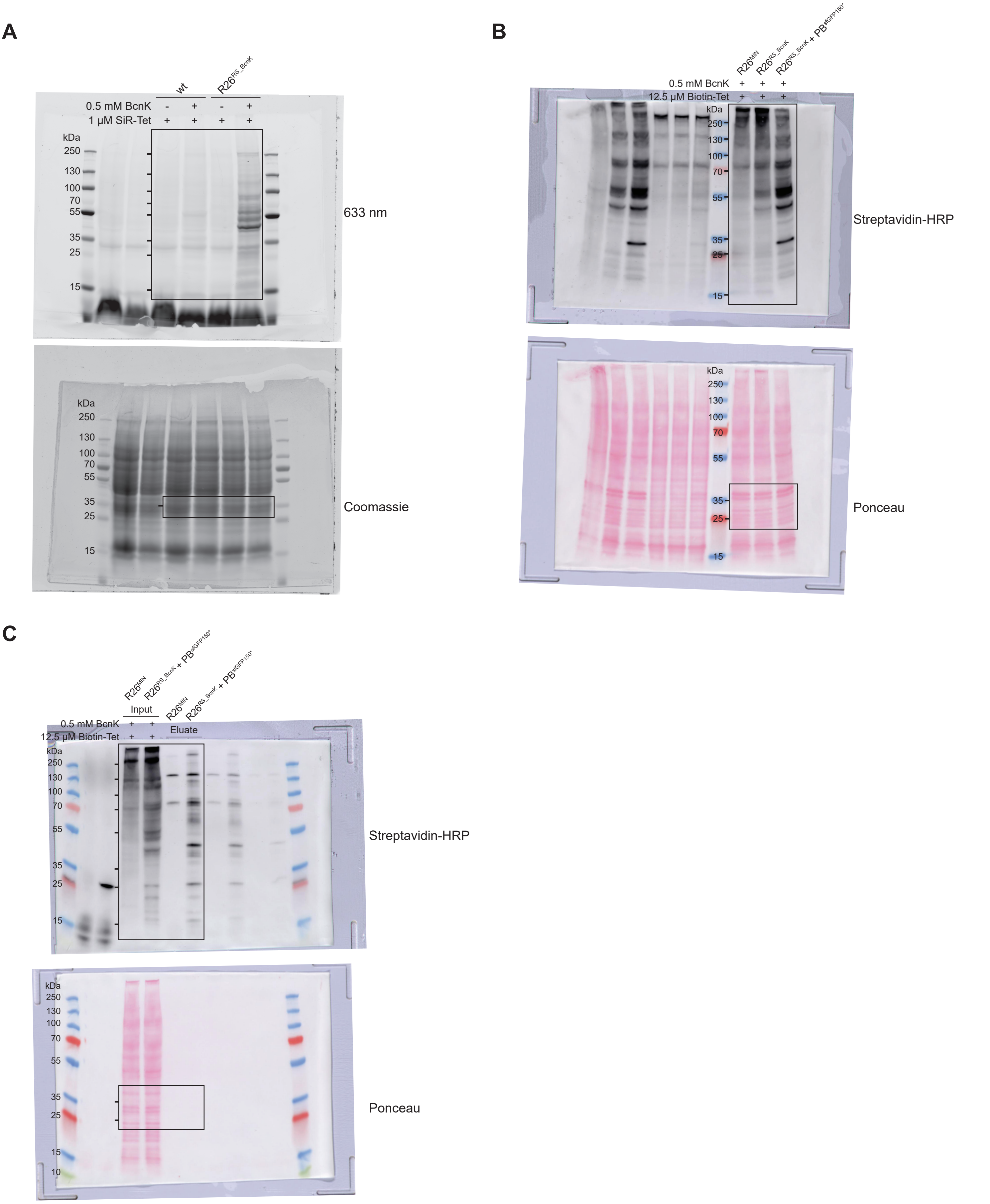
A
wt
R26RS_BcnK
0.5 mM BcnK - + - +
1 µM SiR-Tet + + + +
kDa
250
130
100
70
55
35
25
15
633 nm
Coomassie
B
R26MIN
R26RS_BcnK
R26RS_BcnK + PBsfGFP150*
0.5 mM BcnK + + +
12.5 µM Biotin-Tet + + +
Streptavidin-HRP
Ponceau
C
R26MIN
R26RS_BcnK + PBsfGFP150*
Input
Eluate
0.5 mM BcnK + +
12.5 µM Biotin-Tet + +
10
Streptavidin-HRP
Ponceau

Supplementary Figure 14: (A) Uncropped SDS-Polyacrylamide gel scanned at 633 nm and respective Coomassie staining from Sup. Fig. 3A. **(B)** Uncropped Western blot and respective Ponceau S staining from Sup. Fig. 3B. **(C)** Uncropped Western blot and respective Ponceau S staining from Fig. 2A. **(A-C)** Cropped areas are indicated by black boxes.

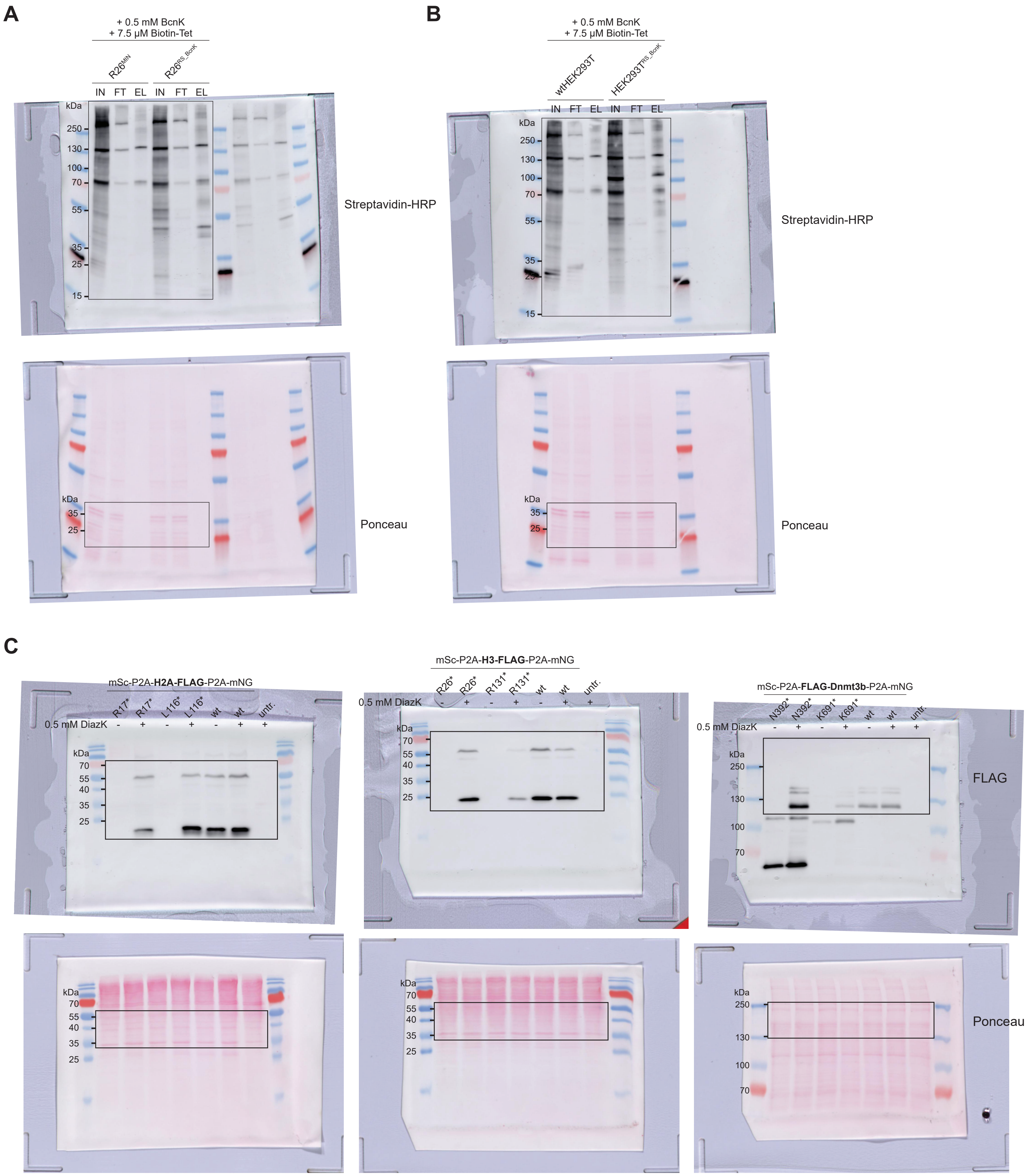
A
+ 0.5 mM BcnK
+ 7.5 µM Biotin-Tet
R26MIN
R26RS_BcnK
IN FT EL IN FT EL
kDa
250
130
100
70
55
35
25
15
Streptavidin-HRP
kDa
35
25
Ponceau
B
+ 0.5 mM BcnK
+ 7.5 µM Biotin-Tet
wtHEK293T
HEK293TRS_BcnK
IN FT EL IN FT EL
kDa
250
130
100
70
55
35
25
15
Streptavidin-HRP
kDa
35
25
Ponceau
C
mSc-P2A-H2A-FLAG-P2A-mNG
R17* R17* L116* L116* wt wt untr.
0.5 mM DiazK - + - + - + +
kDa
70
55
40
35
25
mSc-P2A-H3-FLAG-P2A-mNG
R26* R26* R131* R131* wt wt untr.
0.5 mM DiazK - + - + - + +
kDa
70
55
40
35
25
mSc-P2A-FLAG-Dnmt3b-P2A-mNG
N392* N392* K691* K691* wt wt untr.
0.5 mM DiazK - + - + - + +
kDa
250
130
100
70
FLAG
kDa
70
55
40
35
25
kDa
70
55
40
35
25
kDa
250
130
100
70
Ponceau

Supplementary Figure 15: (A-C) Uncropped Western blot and respective Ponceau S staining from Sup. Fig. 3D (A), Sup. Fig. 3E (B), and Sup. Fig. 10A (C). Cropped areas are indicated by black boxes.

REFERENCES

1. Elliott,T.S., Bianco,A., Townsley,F.M., Fried,S.D. and Chin,J.W. (2016) Tagging and Enriching Proteins Enables Cell-Specific Proteomics. *Cell Chem Biol*, **23**, 805–815.

2. Qiu,C., Sawada,K., Zhang,X. and Cheng,X. (2002) The PWWP domain of mammalian DNA methyltransferase Dnmt3b defines a new family of DNA-binding folds. *Nat. Struct. Biol.*, **9**, 217–224.

3. Arnold,K., Bordoli,L., Kopp,J. and Schwede,T. (2006) The SWISS-MODEL workspace: a web-based environment for protein structure homology modelling. *Bioinformatics*, **22**, 195–201.

4. Guo,X., Wang,L., Li,J., Ding,Z., Xiao,J., Yin,X., He,S., Shi,P., Dong,L., Li,G., *et al.* (2015) Structural insight into autoinhibition and histone H3-induced activation of DNMT3A. *Nature*, **517**, 640–644.

5. Smith,D. and Yarus,M. (1989) Transfer RNA structure and coding specificity. I. Evidence that a D-arm mutation reduces tRNA dissociation from the ribosome. *J. Mol. Biol.*, **206**, 489–501.
