# Supplementary material for: Identification of permissive amber suppression sites for efficient non-canonical amino acid incorporation in mammalian cells
Source: Nucleic Acids Res. 2021 Mar 3;49(11):e62. doi: 10.1093/nar/gkab132 (PMC8216290; doi:10.1093/nar/gkab132)
Supplement: gkab132_Supplemental_Files [file gkab132_supplemental_files.zip › Supplementary_Material_and_Methods (1).pdf]

Identification of permissive amber suppression sites for efficient non-canonical amino acid incorporation in mammalian cells

Michael D. Bartoschek, Enes Ugur, Tuan-Anh Nguyen, Geraldine Rodschinka, Michael Wierer, Kathrin Lang and Sebastian Bultmann

SUPPLEMENTARY MATERIAL AND METHODS

Plasmid construction

General

For PCR amplification, restriction digest, or ligation, Phusion DNA polymerase (F530, Thermo Fisher Scientific), FastDigest enzymes (Thermo Fisher Scientific), or T4 DNA ligase (EL0011, Thermo Fisher Scientific) were used according to the manufacturer's instructions. For homology directed assembly of multiple DNA fragments (Gibson cloning), the NEBuilder HiFi DNA Assembly Master Mix (E2621, New England Biolabs) was used according to the manufacturer's instructions. SmartLadder (MW-1700-10, Eurogentec) and SmartLadderSF (MW-1800-04, Eurogentec) were used as molecular weight markers. DNA concentrations were measured on NanoPhotometer NP80 (Implen). For assembly of oligonucleotides to generate dsDNA fragments, 40 µM of the respective forward and reverse oligonucleotides were annealed in 1x NEBuffer 4 (B7004S, New England Biolabs) and following cycling settings: 95°C/5min - [T-1°C/60s] x 91 - 4°C/∞. The eukaryotic amber suppression plasmids pPiggyBac_4×U6-PylT(U25C)/EF1-MmPylS-IRES-Neo, pPiggyBac_4×U6-PylT(U25C)/EF1-mCherry-TAG-EGFP-IRES-Neo, and pPiggyBac_4×U6-PylT(U25C)/EF1-sfGFP(150TAG)-IRES-Puro for generation of stable cell lines by PiggyBac transposition were a gift from Jason W. Chin (1). All plasmids generated and used in this study were verified by Sanger sequencing (Mix2Seq, Eurofins Genomics) and are listed in Sup. Tab. 1. Oligonucleotides were purchased from Integrated DNA Technologies (IDT) and are listed in Sup. Tab. 2. All plasmids cloned in this study are available on Addgene (##167491-99; www.addgene.org; see Sup. Tab. 1).

NES-PylS vectors (NES-PylS/4xPylT)

To mutate the *Methanosarcina mazei* wildtype pyrrolysyl-tRNA synthetase (wtPylRS; encoded by *wtPylS*) in pPiggyBac_4×U6-PylT(U25C)/EF1-MmPylS-IRES-Neo, the respective DNA fragment flanked by Bpu1102I and NotI sites was replaced by restriction cloning with a dsDNA repair template (gBlocks® Gene Fragments, Integrated DNA Technologies) harboring the respective mutations for DiazK (*PylS_DiazK*: Y306M, L309A, C348A) or BcnK (*PylS_BcnK*: Y306G, C348V) aminoacylation. Evolved *M. barkeri PylS_DiazK* or *PylS_BcnK* coding sequences were originally reported for the incorporation of lysine-based amino acids harboring bromoalkyl chains (2) or methyl-substituted tetrazine (3), respectively (note: mutation sites in *M. barkeri PylS* differ by -35 codons from *M. mazei PylS* used in this study). *M. barkeri PylS_BcnK* was recently demonstrated to efficiently incorporate BcnK in mammalian cells (4). LC-MS analysis of $sfGFP^{N150DiazK}$ confirmed selective incorporation of DiazK in response to the amber stop codon in HEK293T cells expressing PylRS_DiazK (Sup. Fig. 1D).

To construct the PiggyBac and MIN-tag recombination compatible vector PB_attB_wtPylRS-Neo_4xPylT, a dsDNA fragment harboring the *attB* site for Bxb1 (BttB) and the *attP* site for ϕC31 (CttP) mediated recombination was

assembled by annealing of BttB_CttP.fwd with BttB_CttP.rev. The annealed *attB* fragment was then cloned into the Eco32I site of pPiggyBac_4×U6-PylT(U25C)/EF1-MmPylS-IRES-Neo via cut-ligation.

To N-terminally tag PylRSs with a nuclear export signal (NES: NH_2-ACPVPLQLPPLERLTLD-COOH) (5), the NES dsDNA fragment was assembled by annealing NES.fwd with NES.rev. PylRSs were subcloned using XbaI and BamHI sites into pUC57-Gent (a gift from Martin Parniske, Addgene plasmid #54338; (6)) generating pUC57-Gent_PylRS and amplified using NES-PylRS.fwd and NES-PylRS.rev with overhangs for subsequent DNA assembly with the NES fragment. The annealed NES fragment was then cloned into the amplified pUC57-Gent_PylRS backbone by Gibson assembly generating pUC57-Gent_NES-PylRS vectors. The wtPylRS cassette in PB_attP_wtPylRS-Neo_4xPylT was finally replaced with NES-PylRS cassettes from pUC57-Gent_NES-PylRS vectors by restriction cloning using XbaI and BamHI sites generating the three PB_attB_NES-PylRS-Neo_4xPylT plasmids PB_attB_NES-wtPylRS-Neo_4xPylT, PB_attB_NES-PylRS_DiazK-Neo_4xPylT, and PB_attB_NES-PylRS_BcnK-Neo_4xPylT.

The neomycin cassette in PB_attB_NES-PylRS-Neo_4xPylT vectors was exchanged with puromycin from PB_attP_mSc-P2A-GOI*-3xFLAG-P2A-mNG-Puro_4xPylT by restriction cloning using NotI and KspAI sites generating PB_attB_NES-wtPylRS-Puro_4xPylT, PB_attB_NES-PylRS_DiazK-Puro_4xPylT, and PB_attB_NES-PylRS_BcnK-Puro_ 4xPylT.

mCherry-EGFP control reporter construct (4xPylT/mCherry-EGFP)

To remove the amber stop codon in the pPiggyBac_4×U6-PylT(U25C)/EF1-mCherry-TAG-EGFP-IRES-Neo reporter, the mCherry-TAG-EGFP cassette was subcloned using XbaI and BamHI sites into pUC57-Gent generating pUC57-Gent_mCherry-TAG-EGFP. Subsequently, the amber stop codon was removed by overlap extension PCR using mCh-GFP.fwd and mCh-GFP.rev generating pUC57-Gent_mCherry-EGFP. The mCherry-TAG-EGFP cassette in pPiggyBac_4×U6-PylT(U25C)/EF1-mCherry-TAG-EGFP-IRES-Neo was finally exchanged with mCherry-EGFP from pUC57-Gent_mCherry-EGFP by restriction cloning using XbaI and BamHI sites.

mSc/mNG dual-fluorescence reporter (mSc-P2A-GOI-P2A-mNG/4xPylT)*

To construct the amber suppression reporter PB_attP_mSc-P2A-GOI*-3xFLAG-P2A-mNG-Puro_4xPylT, in which the gene of interest harboring the amber stop codon (GOI*) is flanked by the bright fluorescent proteins mScarlet (mSc; (7)) and mNeonGreen (mNG; (8)) separated by P2A self-cleaving peptides optimized for high cleavage efficiency (9), the parts XbaI-mSc-P2A-GOI-3xFLAG-EcoRI, EcoRI-P2A-NheI, and NheI-mNG-NotI were synthesized as dsDNA fragments (gBlocks® Gene Fragments, Integrated DNA Technologies) and subcloned into the RruI site of pUC57-Gent by cut-ligation. Using the respective flanking restriction sites, these dsDNA fragments were cloned into XbaI and NotI linearized pPiggyBac_4×U6-PylT(U25C)/EF1-MmPylS-IRES-Neo that was prior to this step modified as follows: (1) The *attP* site for Bxb1 (BttP) and the *attB* site for ϕC31 (CttB) mediated recombination were integrated by annealing CttB_BttP.fwd with CttB_BttP.rev and subsequent cloning into the Eco32I site via cut-ligation; (2) The SfaAI site was mutated by annealing SfaAI_mut.fwd with SfaAI_mut.rev and subsequent cloning into the SfaAI site via cut-ligation; and finally (3) The neomycin cassette was replaced by puromycin by amplifying the IRES-Puro cassette of pPiggyBac_4×U6-PylT(U25C)/EF1-sfGFP(150TAG)-IRES-Puro using Puro_NotI.fwd and Puro_SalI.rev, subcloning into the RruI site of pUC57-Gent by cut-ligation generating pUC57-Gent_IRES-Puro, and exchanging the IRES-Neo cassette with IRES-Puro from pUC57-Gent_IRES-Puro by restriction cloning using NotI and SalI sites.

H2A, H3*, and Dnmt3b* amber mutants*

Mus musculus H2A (NCBI ref. seq. NM_175660.3) and *H3.2* (NCBI ref. seq. NM_178203.2) were synthesized as dsDNA fragments (gBlocks® Gene Fragments, Integrated DNA Technologies) and subcloned into the RruI site of pUC57-Gent by cut-ligation. Amber mutations were integrated into *H2A* and *H3* by overlap extension PCR on pUC57-Gent_H2A or pUC57-Gent_H3 using the respective oligonucleotides (H2A_XX*.fwd/.rev and H3_XX*.fwd/.rev from Sup. Tab. 2). H2A or H3 with amber mutations were then amplified using H2A_P2A.fwd and H2A_FLAG.rev or H3_P2A.fwd and H3_FLAG.rev with overhangs for subsequent DNA assembly. PB_attP_mSc-P2A-H2A-3xFLAG-P2A-mNG-Puro_4xPylT and PB_attP_mSc-P2A-H3-3xFLAG-P2A-mNG-Puro_4xPylT amber mutant vectors were finally assembled by Gibson cloning into SfaAI and XhoI linearized PB_attP_mSc-P2A-GOI*-3xFLAG-P2A-mNG-Puro_4xPylT.

To generate PB_attP_mSc-P2A-3xFLAG-Dnmt3b-His-P2A-mNG-Puro_4xPylT amber mutant vectors, first the TAG stop codon in the attB-GFP-Dnmt3b1-Poly(A) vector coding for *M. musculus* DNMT3B (Addgene plasmid #65531, (10)) was mutated to TAA by overlap extension PCR using Dnmt3b_TAA.fwd and Dnmt3b_TAA.rev. To C-terminally tag DNMT3B with a 6xHis-tag, *Dnmt3b* was subsequently amplified from the vector with Dnmt3b_His.fwd and Dnmt3b_His.rev and subcloned into the RruI site of pUC57-Gent by cut-ligation. The Dnmt3b-His cassette was then flanked with SfaAI and EcoRI sites by amplification with Dnmt3b_SfaAI.fwd and Dnmt3b_EcoRI.rev and cut-ligation into the RruI site of pUC57-Gent generating pUC57-Gent_Dnmt3b-His. To N-terminally tag Dnmt3b-His with a 3xFLAG-tag, pUC57-Gent_Dnmt3b-His was amplified using Dnmt3b_FLAG.fwd and Dnmt3b_FLAG.rev and subsequently assembled by Gibson cloning with the 3xFLAG fragment that was amplified from p3xFLAG-CMV-7 (E7408, Sigma-Aldrich) using FLAG.fwd and FLAG.rev. Amber mutations were integrated into *Dnmt3b* by overlap extension PCR on pUC57-Gent_3xFLAG-Dnmt3b-His using the respective oligonucleotides (D3b_XXX*.fwd/.rev from Sup. Tab. 2). 3xFLAG-Dnmt3b-His fragments with amber mutations were finally cloned into PB_attP_mSc-P2A-GOI*-3xFLAG-P2A-mNG-Puro_4xPylT using SfaAI and EcoRI.

context amber mutants (mSc-P2A-context*-P2A-mNG/4xPylT)*

To construct the PB_attP_mSc-P2A-context*-P2A-mNG-Puro_4xPylT amber suppression reporters, the 3xFLAG-tag flanked by XhoI and EcoRI sites in the PB_attP_mSc-P2A-wtH2A-3xFLAG-P2A-mNG-Puro_4xPylT vector was replaced by restriction cloning with *context** dsDNA fragments that were prior to that assembled by annealing the respective Co_X.X_XXXX(*).fwd with Co_X.X_XXXX(*).rev oligonucleotide (Sup. Tab. 2; X.X, XXXX, and * indicate iPASS score, encoded amino acids, and amber mutant respectively).

Chemicals and chemical synthesis

General information

All chemicals and solvents were obtained from commercial suppliers and used without further purification unless otherwise stated. N_ε-(*tert*-Butoxycarbonyl)-*L*-Lysine (BocK) was obtained from Carbolution and N6-((((1R,8S)-Bicyclo[6.1.0]non-4-yn-9-yl)methoxy)carbonyl)-*L*-lysine (BcnK) was purchased from Synaffix respectively and prepared in stock solutions for further experiments without any additional treatment. The synthesis of diazirine-Lysine (DiazK) was performed according to a previously published protocol (11) with modified conditions (see below). The synthesis of the tetrazine was performed as previously published (12).

Flash column chromatography used for product purification was performed on silica gel 60 (230-400 mesh). Thin-layer chromatography (TLC) was performed on Merck Millipore silica gel 60 F-254 plates. The developed silica plates were visualized by UV light (254 nm) and/or staining with ninhydrin.

Small Molecule LC-MS was performed on an Agilent Technologies 1260 Infinity LC-MS system with a Phenomenex AerisTM Peptide XB-C18 column (100 x 2.1 mm, 3.6 µm) coupled to a 6310 Quadrupole spectrometer. Samples were analysed both in positive and negative mode followed by UV absorbance at 193, 254 and/or 280 nm.

Reverse phase HPLC purification was carried out on a Shimadzu LC-20AT Prominence system with a Phenomenex Luna C18, 5 µm (4.6 x 250 mm) column. The solvent system consists of buffer A (MilliQ H_2O + 0.1 % FA) and buffer B (MeCN + 0.1 % FA), which were used without filtration.

NMR spectra were recorded on a Bruker 500 UltraShieldTM. Chemical shifts (δ), reported in ppm, are referenced to the residual proton and carbon solvent signals. Coupling constants (J) are reported in Hertz (Hz) while peak multiplicities are described as follows: s (singlet), bs (broad singlet), d (doublet), dd (doublet of doublets), td (triplet of doublets), t (triplet), m (multiplet).

Synthesis of 2-(3-Methyldiazirin-3-yl)-ethan-1-ol

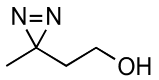

To 4-Hydroxy-2-butanone (4 g, 45 mmol) on ice was added NH_3 (7 M in MeOH, 35 mL). After 3 h, hydroxylamine-O-sulfonic acid (5.65 g, 1.1 eq., 50 mmol) was added and the reaction was stirred overnight, gradually allowed to warm up to room temperature. The mixture was filtered and washed (with MeOH) through Celite, the solvent removed and the crude intermediate taken up in MeOH (40 mL) and Et_3N (8 mL) and stirred on ice. I_2 was added portion-wise until the reaction mixture maintained a dark yellow/brown color. After 3 h, the solvent was removed and taken up in Et_2O (150 mL). The organic phase was washed with 1 M HCl (80 mL). The aqueous phase was extracted again with Et_2O (150 mL). The combined organic phase was washed with 20% (w/v) $Na_2S_2O_3$ (100 mL) and brine (100 mL). The organic phase was dried over Na_2SO_4, filtered and removed under reduced pressure to yield a dark yellow oil (yield: 1.88 g, 42%). The crude product was used without further purification. Chemical shifts were according to literature (11).

Synthesis of 2-(3-Methyldiazirin-3-yl)-ethyl (4-nitrophenyl) carbonate

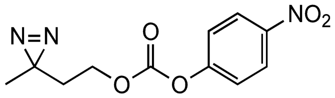

To a solution of 2-(3-Methyldiazirin-3-yl)-ethan-1-ol (1.88 g, 18.78 mmol) in DCM (100 mL) on ice was added 4-nitrophenyl chloroformate (4.54 g, 1.2 eq.) and pyridine (1.82 mL, 1.2 eq.) and the reaction was stirred overnight, gradually allowed to warm up to room temperature. The reaction mixture was concentrated under reduced pressure and purified by flash chromatography (10 -> 15% EtOAc in pentane) to obtain the final product as a yellow oil (yield: 3.08 g, 62%).

^{1}H NMR (300 MHz, $CDCl_3$) δ = 1.12 (s, 3H), 1.80 (t, J = 6.4 Hz, 2H), 4.25 (t, J = 6.4 Hz, 2H), 7.40 (d, J = 9.2 Hz, 2H), 8.29 (d, J = 9.2 Hz, 2H). **^{13}C NMR (75 MHz, $CDCl_3$)** δ = 20.0, 23.7, 33.9, 64.4, 122.0, 125.5, 145.6, 152.5, 155.6. NMR spectra are depicted in Sup. Fig. 13A.

Synthesis of N[2]-(tert-butoxycarbonyl)-N[6]-((2-(3-methyl-3H-diazirin-3-yl)ethoxy)carbonyl)-l-lysine

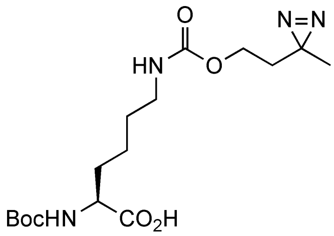

To a solution of 2-(3-Methyldiazirin-3-yl)-ethyl (4-nitrophenyl) carbonate (11.61 mmol, 3.08 g) in dioxane (30 mL) was added Nα-Boc-Lysine (3.43 g, 1.2 eq.) and Et_3N (3.22 mL, 2.0 eq.) and the reaction was stirred overnight at room temperature. The reaction mixture was concentrated under reduced pressure and purified by flash column chromatography (2% MeOH in DCM -> 5% MeOH in DCM + 0.5% AcOH) to yield the final product as a colorless oil (2.58 g, 60%). Chemical shifts were according to literature (11).

LC-MS (m/z): calc for $C_{16}H_{28}N_4O_6$ [M-H$^+$]: 371.2; found: 371.1.

Synthesis of N[6]-((2-(3-methyl-3H-diazirin-3-yl)ethoxy)carbonyl)-l-lysine TFA salt (DiazK)

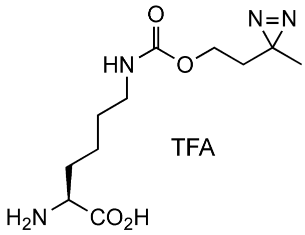

To a stirred solution of N[2]-(tert-butoxycarbonyl)-N[6]-((2-(3-methyl-3H-diazirin-3-yl)ethoxy) carbonyl)-L-lysine (4.84 g) in DCM (28 mL) on ice was added TFA (7 mL) and H_2O (1 mL). After 3 h, the solvent was removed, the crude product precipitated in ice-cold Et_2O and pelleted by centrifugation. This was repeated twice and the final product was lyophilised to obtain the final product as a white powder (yield: 4.63 g, 96%, TFA salt). Chemical shifts were according to literature (11).

LC-MS (m/z): calc for. $C_{11}H_{20}N_4O_4$ [M+H$^+$]: 273.2; found: 273.2.

Synthesis of Biotin-tetrazine conjugate

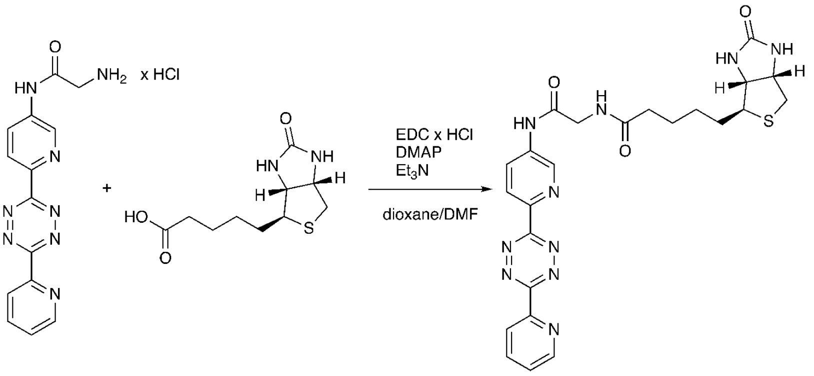

To a solution of tetrazine (1.0 eq., 18.4 mg, 53.4 µmol) and D-biotin (1.7 eq.) were added EDCxHCl (2.2 eq.), DMAP (0.1 eq.) and Et_3N (3.4 eq.) in dioxane/DMF (5:1, 6 mL in total) and the reaction was stirred over two days at room temperature until LC-MS indicated reaction completion. The solvent was removed and the crude product was purified by preparative, reverse phase HPLC to yield the final Biotin-tetrazine conjugate as a pink powder (gradient 10 -> 40% B in buffer A, 5 mg after lyophilisation).

^{1}H NMR (400 MHz, DMSO-d$_6$) δ = 1.26 - 1.66 (m, 6H), 2.16 (t, J = 7.3 Hz, 2H), 2.54 (d, J = 12.4 Hz, 1H), 2.79 (dd, *J* = 12.4, 5.1 Hz, 1H), 3.04 - 3.11 (m, 1H), 3.94 (d, 5.8 Hz, 2H), 4.05 - 4.16 (m, 1H), 4.28 (dd, *J* = 7.5, 5.2 Hz, 1H), 6.32 (bs, 1H), 6.38 (bs, 1H), 7.69 (ddd, *J* = 7.6, 4.8, 1.0 Hz, 1H), 8.11 (td, *J* = 7.8, 1.7 Hz, 1H), 8.22 (t, *J* = 5.8 Hz, 1H), 8.37 (dd, *J* = 8.7, 2.5 Hz, 1H), 8.53 - 8.57 (m, 1H), 8.60 (d, *J* = 8.7 Hz, 1H), 8.87 - 8.91 (m, 1H), 9.02 (d, *J* = 2.4 Hz, 1H), 10.63 (s, 1H). **^{13}C NMR (100 MHz, DMSO-d$_6$)** δ = 25.2, 28.1, 28.2, 34.9, 42.8, 55.4, 59.3, 61.1, 124.2, 125.0, 126.4, 126.6, 137.8, 138.2, 141.3, 144.1, 150.2, 150.6, 162.8, 163.1, 169.3, 172.9. NMR spectra are depicted in Sup. Fig. 13B. **LC-MS (m/z)**: calc. for $C_{24}H_{26}N_{10}O_3S$ [M+H$^+$]: 535.2; found: 535.2.

Transient transfections

Cell lysates for sfGFP^{N150DiazK} pulldown

700,000 stable HEK293T^{RS_DiazK} cells were seeded per p100 plate and after 2 h 0.5 mM DiazK was added. Cells were transfected after 4 h with 15 µg *4xPylT/sfGFP^{N150*}* plasmid using Lipofectamine3000 (Thermo Fisher Scientific) according to the manufacturer's instructions and incubated for 72 h before harvesting.

Cell lysates for Western blotting

500,000 stable HEK293T^{RS_DiazK} cells per 6-well were seeded into DiazK containing medium 4 h before transfection. Cells were transfected with 2.5 µg of the respective plasmid using Lipofectamine3000 (Thermo Fisher Scientific) according to the manufacturer's instructions and incubated for 24 h before harvesting.

Immunoaffinity purification of amber suppressed sfGFP^{N150DiazK} and analysis by mass spectrometry

Preparation of cellular extracts

Transiently transfected HEK293T^{RS_DiazK} cells were washed once in p100 plates with ice cold PBS, scraped on ice, resuspended in 1.5 mL ice cold PBS, and collected in 2 mL tubes by centrifugation at 500 g and 4°C for 6 min. Cell pellets were washed on ice two times by resuspending in 1 mL ice cold PBS and centrifugation at 500 g and 4°C for 6 min. Pellets were shock frozen in liquid N_2 and stored at -80°C.

Cell pellets from one p100 were lysed on ice for 30 min with gentle pulse-vortexing every 10 min in 500 µL of ice cold RIPA buffer (50 mM TRIS/HCl pH 8.0, 150 mM NaCl, 0.1% UltraPure™ SDS Solution (24730020, Invitrogen), 0.5% sodium deoxycholate detergent, 1% Triton X-100; freshly add 1x cOmplete™ EDTA-free Protease Inhibitor Cocktail (04693132001, Roche), 2 mM PMSF, 0.1 U/µL Benzonase). Lysates were cleared by centrifugation at 20,000 g and 4°C for 15 min and supernatants collected.

GFP-Trap pulldown

50 µL slurry of GFP-Trap Agarose (Chromotek) were washed three times in 750 µL IP wash buffer (20 mM TRIS/HCl pH 7.5, 150 mM NaCl, 0.5 mM EDTA) and lysates added. Samples were then incubated for 1.5 h at 4°C with end-over-end rotation. After 1.5 h, beads were collected by centrifugation for 2 min at 2,300 g and 4°C. Beads were washed three times on ice by resuspending in 750 µL IP wash buffer and centrifugation for 2 min at 2,300 g and 4°C. During the last washing step, GFP-trap beads were transferred to a fresh 1.5 mL tube. For mass spectrometry analysis, proteins were eluted from beads by resuspending in 30 µL 0.15% (v/v) Trifluoroacetic acid (TFA), incubation for 2 min on ice, and collection of supernatant by centrifugation for 2 min at 2,300 g and 4°C. Elution was repeated once and the sample stored at -20°C for subsequent analysis by mass spectrometry.

Mass spectrometry analysis

Protein MS measurement was performed on an Agilent Technologies 1260 Infinity LC-MS system with a Phenomenex JupiterTM C4 column (2 x 150 mm, 5 µm) and recording in positive mode. Protein MS was deconvoluted using OpenLab ChemStation Edition Software C.01.07.SR3 [465].

Labeling of amber suppressed endogenous proteins with fluorophore

Proteomic incorporation of BcnK at amber stop codons

Stable transgenic or wildtype mESCs were seeded at 10% confluency in T25 cell culture flasks. After 2 h 0.5 mM BcnK diluted in 3 volumes of 1 M HEPES (15630056, Thermo Fisher Scientific) was added and cells were grown for 42 h.

Chemoselective labeling of BcnK tagged proteomes with SiR-tetrazine conjugate in living cells

After 42 h, mESCs were washed once with Dulbecco's PBS (D8537, Sigma-Aldrich), incubated for 90 min with fresh medium, washed a second time with PBS, and incubated for 30 min with 1 µM SiR-tetrazine conjugate (SC008, Spirochrome) diluted in fresh medium. Subsequently, cells were washed once with PBS and incubated for 60 min with fresh medium. For harvesting, mESCs were washed once with PBS in T25 cell culture flasks, dissociated with 500 µL StemPro™ Accutase™ Cell Dissociation Reagent (A1110501, Gibco), resuspended in 4 ml ice cold PBS, and collected by centrifugation at 200 g and 4°C for 5 min. Cell pellets were washed on ice two times by resuspending in 5 mL ice cold PBS and centrifugation at 200 g and 4°C for 5 min. During the first wash, single cells were counted using a Scepter™ Cell Counter (Millipore). Pellets were shock frozen in liquid N_2 and stored at -80°C.

Visualization by SDS-PAGE

1.5 x 10^6 cells were lysed in 100 µL lysis buffer (10 mM HEPES pH 7.9, 1.5 mM $MgCl_2$, 10 mM KCl; freshly add 0.1% SDS, 2 mM phenylmethylsulfonyl fluoride (PMSF), 1x cOmplete™ EDTA-free Protease Inhibitor Cocktail (04693132001, Roche)) and sonicated at 4°C for 10 min in 1.5 mL tubes using a Bioruptor® Plus sonication device (Diagenode) with the following settings: high intensity, 30 s on/30 s off cycle. Lysates were then cleared by centrifugation at 20,000 g and 4°C for 15 min and supernatants boiled for 10 min at 95°C in 1x Laemmli supplemented with 20 mM DTT. Lysates were separated by 4-20% SDS-PAGE (Bio-Rad Laboratories) on a Mini-Protean Tetra system (Bio-Rad Laboratories) at 80 V for 30 min followed by 120 V for 80 min. PageRuler™ Plus Prestained (26620, Thermo Fisher Scientific) was used as protein marker. SDS-gels were imaged for in-gel fluorescence on a Typhoon Trio+ fluorescent imaging system (633 nm, filter BP670; GE Healthcare) and subsequently stained with Coomassie Blue.

Western blotting

3xFLAG-tagged H2A, H3*, and Dnmt3b**

Transiently transfected $HEK293T^{RS\_DiazK}$ cells were washed twice in 6-wells with ice cold PBS, scraped on ice, resuspended in 500 µL ice cold PBS, and collected in 1.5 mL tubes by centrifugation at 500 g and 4°C for 6 min. After aspirating the supernatant, pellets were shock frozen in liquid N_2 and stored at -80°C.

Cell pellets from one 6-well were lysed on ice for 30 min with gentle pulse-vortexing every 10 min in 4 volumes of ice cold RIPA buffer (50 mM TRIS/HCl pH 8.0, 150 mM NaCl, 0.1% UltraPure™ SDS Solution (24730020, Invitrogen), 0.5% sodium deoxycholate detergent, 1% Triton X-100; freshly add 1x cOmplete™ EDTA-free Protease Inhibitor Cocktail (04693132001, Roche)). Lysates were cleared by centrifugation at 20,000 g and 4°C for 15 min and supernatants collected. Protein concentrations were then determined using the Pierce™ BCA Protein Assay Kit (23225, Thermo Fisher Scientific) according to the manufacturer's instructions for microplate settings. Cleared cell lysates were

diluted with 1x Laemmli supplemented with 20 mM DTT to a final concentration of 2 mg/mL protein and boiled for 10 min at 95°C.

Lysates were separated by 8% (DNMT3B) or 15% (H2A/H3) SDS-PAGE on a Mini-Protean Tetra system (Bio-Rad Laboratories) at 80 V for 30 min followed by 120 V for 90 min. Wet transfer was performed for 90 min at 300 mA to a nitrocellulose membrane (0.2 µm pore size) in a Mini Trans-Blot cell (Bio-Rad Laboratories). After blotting and subsequent Ponceau S staining, the membrane was blocked for 1 h at room temperature with 5% (w/v) milk powder in TBS-T (50 mM TRIS/HCl pH 7.5, 150 mM NaCl, 0.075% Tween20). The primary monoclonal anti-FLAG antibody (mouse; F3165, Sigma-Aldrich) was diluted 1:1,000 in 1% (w/v) milk powder TBS-T and the membrane incubated overnight at 4°C. The secondary polyclonal anti-mouse antibody (rabbit; A9044, Sigma-Aldrich) conjugated to horseradish peroxidase (HRP) was diluted 1:5,000 in 1% (w/v) milk powder TBS-T and the membrane incubated for 1 h at room temperature. Membranes were washed six times for 10 min each at room temperature with TBS-T after primary and secondary antibody incubation. Washing and incubation steps were carried out in a 50 mL falcon with gentle rotation. PageRuler™ Prestained (26617, Thermo Fisher Scientific) or PageRuler™ Plus Prestained (26620, Thermo Fisher Scientific) were used as protein marker. Blots were visualized using the Amersham ECL Prime Western Blotting Detection Reagent (RPN2232, GE Healthcare) and imaged at an Amersham Imager 600 (GE Healthcare).

Biotin labeled proteomes

Input, unbound fraction, and eluate samples were separated by 4-12% SDS-PAGE (Smobio Technology) on a Mini-Protean Tetra system (Bio-Rad Laboratories) at 80 V for 15 min followed by 140 V for 60 min. Wet transfer was performed for 90 min at 300 mA to a nitrocellulose membrane (0.2 µm pore size) in a Mini Trans-Blot cell (Bio-Rad Laboratories). After blotting and subsequent Ponceau S staining, biotinylated proteins were detected using the Gelshift™ Chemiluminescent EMSA Kit (37341, Active Motif) with modifications. Briefly, the membrane was washed 3 times for 10 min each with 20 mL TBS-T (50 mM TRIS/HCl pH 7.5, 150 mM NaCl, 0.075% Tween20), incubated for 15 min with the provided Streptavidin-HRP conjugate diluted 1:300 in 15 mL of the provided Blocking Buffer solution, rinsed briefly with 20 mL TBS-T, washed 3 times for 10 min each with 20 mL TBS-T, incubated for 5 min in 15 mL of the provided Substrate Equilibration Buffer solution, and finally visualized using the provided Chemiluminescent Working solution according to the manufacturer's instructions. Each washing and incubation step was carried out in a fresh container at room temperature with gentle shaking on an orbital shaker. PageRuler™ Plus Prestained (26620, Thermo Fisher Scientific) was used as protein marker. Blots were imaged at an Amersham Imager 600 (GE Healthcare).

SUPPLEMENTARY TABLES

Supplementary Table 1: Plasmids cloned and used in this study.

Name (*alternative name*)	Use	Reference
PB_attB_NES-wtPylRS-Neo_4xPylT (*NES-wtPylS/4xPylT*)	Expression of *Methanosarcina mazei* wildtype pyrrolysyl-tRNA synthetase fused to a nuclear export signal (NES-wtPylRS). Stable integration via PiggyBac transposition (PB) or Bxb1-mediated recombination (attB). Neomycin (Neo) as selection marker. Co-expression of 4x $tRNA^{Pyl}_{CUA}$ (PylT).	This study (Addgene #167491)
PB_attB_NES-PylRS_DiazK-Neo_4xPylT (*NES-PylS_DiazK/4xPylT*)	See PB_attB_NES-wtPylRS-Neo_4xPylT. wtPylRS with Y306M, L309A, C348A mutations for DiazK aminoacetylation.	This study (Addgene #167492); *PylS* mutant (2)
PB_attB_NES-PylRS_BcnK-Neo_4xPylT (*NES-PylS_BcnK/4xPylT*)	See PB_attB_NES-wtPylRS-Neo_4xPylT. wtPylRS with Y306G, C348V mutations for BcnK aminoacetylation.	This study (Addgene #167493); *PylS* mutant (3)
PB_attB_NES-wtPylRS-Puro_4xPylT (*NES-wtPylS/4xPylT*)	Expression of *Methanosarcina mazei* wildtype pyrrolysyl-tRNA synthetase fused to a nuclear export signal (NES-wtPylRS). Stable integration via PiggyBac transposition (PB) or Bxb1-mediated recombination (attB). Puromycin (Puro) as selection marker. Co-expression of 4x $tRNA^{Pyl}_{CUA}$ (PylT).	This study (Addgene #167494)
PB_attB_NES-PylRS_DiazK-Puro_4xPylT (*NES-PylS_DiazK/4xPylT*)	See PB_attB_NES-wtPylRS-Puro_4xPylT. wtPylRS with Y306M, L309A, C348A mutations for DiazK aminoacetylation.	This study (Addgene #167495)
PB_attB_NES-PylRS_BcnK-Puro_4xPylT (*NES-PylS_BcnK/4xPylT*)	See PB_attB_NES-wtPylRS-Puro_4xPylT. wtPylRS with Y306G, C348V mutations for BcnK aminoacetylation.	This study (Addgene #167496)
PB_attP_mSc-P2A-GOI*-3xFLAG-P2A-mNG-Puro_4xPylT (*mSc-P2A-GOI*-P2A-mNG/4xPylT*)	Reporter construct to assess amber suppression efficiency of a C-terminally 3xFLAG tagged gene of interest harboring the amber stop codon (GOI*) based on mNG/mSc expression separated from GOI* by self-cleaving peptide P2A. Stable integration via PiggyBac transposition (PB) or Bxb1-mediated recombination (attP). Puromycin (Puro) as selection marker. Co-expression of 4x $tRNA^{Pyl}_{CUA}$ (PylT).	This study
PB_attP_mSc-P2A-wtH2A-3xFLAG-P2A-mNG-Puro_4xPylT	See PB_attP_mSc-P2A-GOI*-3xFLAG-P2A-mNG-Puro_4xPylT. Wildtype H2A (wtH2A) as GOI^{wt} or H2A containing an in-frame amber stop codon (H2A*) as GOI*.	This study (Addgene #167497)
PB_attP_mSc-P2A-wtH3-3xFLAG-P2A-mNG-Puro_4xPylT	See PB_attP_mSc-P2A-GOI*-3xFLAG-P2A-mNG-Puro_4xPylT. Wildtype H3 (wtH3) as GOI^{wt} or H3 containing an in-frame amber stop codon (H3*) as GOI*.	This study (Addgene #167498)
PB_attP_mSc-P2A-3xFLAG-wtDnmt3b-His-P2A-mNG-Puro_4xPylT	See PB_attP_mSc-P2A-GOI*-3xFLAG-P2A-mNG-Puro_4xPylT. Wildtype *de novo* DNA-methyltransferase 3b (wtDnmt3b) as GOI^{wt} or Dnmt3b containing an in-frame amber stop codon (Dnmt3b*) as GOI*. N-terminally 3xFLAG, C-terminally 6xHis tagged.	This study (Addgene #167499)
PB_attP_mSc-P2A-context*-P2A-mNG-Puro_4xPylT (*mSc-P2A-context*-P2A-mNG/4xPylT*)	Varying in-frame amber stop codon sequence contexts (nt positions -6 to +9 with TAG at +1, +2, +3) replace the 3xFLAG tag in PB_attP_mSc-P2A-wtH2A-3xFLAG-P2A-mNG-Puro_4xPylT.	This study

	Also see PB_attP_mSc-P2A-GOI*-3xFLAG-P2A-mNG-Puro_4xPylT.	
attB-GFP-Dnmt3b1-Poly(A)	*Dnmt3b* donor.	Mulholland et al., 2015 (Addgene #65531) (10)
pUC57-Gent	Subcloning.	Binder et al., 2014 (Addgene #54338) (6)
pPiggyBac_4×U6-PylT(U25C) /EF1-MmPylS-IRES-Neo (*4xPylT/PylS*)	Cloning of PB_attB_NES-PylRS-Neo_4xPylT vectors.	Elsässer et al., 2016 (1)
pPiggyBac_4×U6-PylT(U25C) /EF1-mCherry-TAG-EGFP-IRES-Puro (*4xPylT/mCherry-TAG-EGFP*)	Verification of amber suppression with mCherry-TAG-EGFP reporter in transient transfections.	Elsässer et al., 2016 (1)
pPiggyBac_4×U6-PylT(U25C) /EF1-mCherry-EGFP-IRES-Puro (*4xPylT/mCherry-EGFP*)	Control construct of the mCherry-TAG-EGFP reporter lacking the amber stop codon to constitutively express mCherry-EGFP.	This study
pPiggyBac_4×U6-PylT(U25C) /EF1-sfGFP(150TAG)-IRES-Puro (*4xPylT/sfGFP^{N150*}*)	Verification of amber suppression in sfGFP^{N150*}, which harbors the amber stop codon at position 150, after stable integration via PiggyBac transposition.	Elsässer et al., 2016 (1)
pCAG-NLS-HA-Bxb1	Bxb1 mediated recombination.	Hermann et al., 2014 (Addgene #51271) (13)
pSpCas9(BB)-2A-GFP	CRISPR/Cas9 genome engineering.	Ran et al., 2013 (Addgene #48138) (14)

Supplementary Table 2: Oligonucleotides used in this study.

Name	5'-sequence-3'	Use
BttB_CttP.fwd	CCGGCTTGTCGACGACGGCGGTCTCCGTCGTCAGGATCATCCTCGCGAACCCCAACTGGGGTAACCTTTGAGTTCTCTCAGTTGGGGGCC	Cloning of PB_attB_NES-PylRS-Neo_4xPylT
BttB_CttP.rev	GGCCCCCAACTGAGAGAACTCAAAGGTTACCCCAGTTGGGGTTCGCGAGGATGATCCTGACGACGGAGACCGCCGTCGTCGACAAGCCGG	
NES-PylRS.fwd	TGACCCTGGACGACAAGAAGCCCCTGAACA	
NES-PylRS.rev	CAGGGCAGGCCATGGTGGCAAGCTTC	
NES.fwd	CCATGGCCTGCCCTGTGCCTCTGCAGCTGCCTCCTCTGGAGAGGCTGACCCTGGACGACA	
NES.rev	TGTCGTCCAGGGTCAGCCTCTCCAGAGGAGGCAGCTGCAGAGGCACAGGGCAGGCCATGG	
mCh-GFP.fwd	ATGGCACCAATTAGCCATGGTGAGCA	Cloning of 4xPylT/mCherry-EGFP
mCh-GFP.rev	CTAATTGGTGCCATGGTGAAGCTTG	
SfaAI_mut.fwd	CGTCGGAGCTCGCACCAT	Cloning of PB_attP_mSc-P2A-GOI*-3xFLAG-P2A-mNG-Puro_4xPylT
SfaAI_mut.rev	GGTGCGAGCTCCGACGAT	
CttB_BttP.fwd	ACCGCGGTGCGGGTGCCAGGGCGTGCCCTTGGGCTCCCCGGGCGCGTACTCCTCGCGAGGTTTGTCTGGTCAACCACCGCGGTCTCAGTGGTGTACGGTACAAACC	
CttB_BttP.rev	GGTTTGTACCGTACACCACTGAGACCGCGGTGGTTGACCAGACAAACCTCGCGAGGAGTACGCGCCCGGGGAGCCCAAGGGCACGCCCTGGCACCCGCACCGCGGT	

Puro_NotI.fwd	AATTGCGGCCGCGGCCGCGCCCCTCTCC	
Puro_SalI.rev	GTCGACTTAGGCACCGGGCTTG	
H2A_P2A.fwd	GACGTGGAGGAAAACCCTGGACCTGGCGATCGCTCTGGACGCGGAAAG<br>(For H2A^{R3*}:<br>GACGTGGAGGAAAACCCTGGACCTGGCGATCGCTCTGGATAGGGAAAGCAG)	Cloning of PB_attP_mSc-P2A-H2A-3xFLAG-P2A-mNG-Puro_4xPylT
H2A_FLAG.rev	AATCACCGTCATGGTCTTTGTAGTCCTCGAGCTTCCCCTTGGCCTT	
H3_P2A.fwd	GACGTGGAGGAAAACCCTGGACCTGGCGATCGCGCTCGTACTAAGCAGACC<br>(for H3^{R2*}:<br>GACGTGGAGGAAAACCCTGGACCTGGCGATCGCGCTTAGACTAAGCAGACCG;<br>for H3^{Q5*}:<br>GACGTGGAGGAAAACCCTGGACCTGGCGATCGCGCTCGTACTAAGTAGACCG)	Cloning of PB_attP_mSc-P2A-H3-3xFLAG-P2A-mNG-Puro_4xPylT
H3_FLAG.rev	AATCACCGTCATGGTCTTTGTAGTCCTCGAGAGCCCTCTCCCCAC<br>(for H3^{R131*}: AATCACCGTCATGGTCTTTGTAGTCCTCGAGAGC CCTCTCCCCC;<br>for H3^{R134*}: AATCACCGTCATGGTCTTTGTAGTCCTCGAGAGCC TACTCCCCACG)	
Dnmt3b_TAA.fwd	TGAATAAGCGGCCGCAGTTAACG	Cloning of PB_attP_mSc-P2A-3xFLAG-Dnmt3b-His-P2A-mNG-Puro_4xPylT
Dnmt3b_TAA.rev	GGCCGCTTATTCACAGGCAAAGTAGTCCTTCAAG	
Dnmt3b_His.fwd	AAAGGATCCATGAAGGGAGACAGCAGAC	
Dnmt3b_His.rev	TTTTGCGGCCGCTTAGTGATGGTGATGATGATGGCCTTCACAGGCAAAGTAGTCC	
Dnmt3b_SfaAI.fwd	TTGCGATCGCAAGGGAGACAGCAGACATC	
Dnmt3b_EcoRI.rev	TTGAATTCGTGATGGTGATGATGATGG	
Dnmt3b_FLAG.fwd	GGATGACGATGACAAGAAGGGAGACAGCAGACATCT	
Dnmt3b_FLAG.rev	CATGGTCTTTGTAGTCGCGATCGCAACGAGG	
FLAG.fwd	GACTACAAAGACCATGACGG	
FLAG.rev	CTTGTCATCGTCATCCTTG	
R26_sgRNA	CCCGATCCCCTACCTAGCCG	sgRNA to generate R26^{MIN}
R26_toligo	GCCAATCAGCGGAGGCTGCCGGGGCCGCCTAAAGAAGAGGCTGTGCTTTGGGG<br>CTCCGGCTCCTCAGAGAGCCTCGGGTTTGTCTGGTCAACCACCGCGGTCTCAGT<br>GGTGTACGGTACAAACCGCTAGGTAGGGGATCGGGACTCTGGCGGGAGGGCGG<br>CTTGGTGCGTTTGCGGGGATGGGCGGCCGCGGCAGGCCCT	Donor to generate R26^{MIN}
R26_scr.fwd	GAGCGGAAACGCCACTGA	R26^{MIN}/R26^{RS} screening
R26_scr.rev	CTGTCTCACAGAACGGCTCC	R26^{MIN}/R26^{RS} screening
attL_scr.fwd	CCGGCTTGTCGACGACG	R26^{RS} screening
H2A_R3*.fwd	TGGATAGGGAAAGCAGGGTGGCA	Cloning of *H2A** amber mutants
H2A_R3*.rev	TTCCCTATCCAGACATGGTGGCG	
H2A_Q6*.fwd	AAAGTAGGGTGGCAAGGCCC	
H2A_Q6*.rev	CACCCTACTTTCCGCGTCCAGACA	
H2A_R11*.fwd	GGCCTAGGCTAAGGCCAAGACCCG	
H2A_R11*.rev	TAGCCTAGGCCTTGCCACCCTG	
H2A_R17*.fwd	GACCTAGTCCTCCCGGGCCG	
H2A_R17*.rev	AGGACTAGGTCTTGGCCTTAGCGC	
H2A_R20*.fwd	CTCCTAGGCCGGCCTGCAGTTC	
H2A_R20*.rev	CGGCCTAGGAGGAGCGGGTCTTGG	
H2A_Q24*.fwd	CCTGTAGTTCCCCGTAGGCCG	

H2A_Q24*.rev	GGAACTACAGGCCGGCCCG	
H2A_R29*.fwd	AGGCTAGGTGCATCGGCTGCTCC	
H2A_R29*.rev	GCACCTAGCCTACGGGGAACTGCA	
H2A_Q112*.fwd	CATCTAGGCCGTGCTGCTGC	
H2A_Q112*.rev	CGGCCTAGATGTTGGGCAGGACGC	
H2A_L116*.fwd	GCTGTAGCCCAAGAAGACCGAGAGCC	
H2A_L116*.rev	TGGGCTACAGCACGGCCTGGATG	
H2A_T120*.fwd	GAAGTAGGAGAGCCACCACAAGGGC	
H2A_T120*.rev	TCTCCTACTTCTTGGGCAGCAGCA	
H2A_H124*.fwd	CCACTAGAAGGCCAAGGGGAAGGG	
H2A_H124*.rev	CCTTCTAGTGGCTCTCGGTCTTCTTGG	
H3_R2*.fwd	GGCTTAGACTAAGCAGACCGCTCGC	Cloning of *H3** amber mutants
H3_R2*.rev	TAGTCTAAGCCATGGTGGCGACC	
H3_Q5*.fwd	TAAGTAGACCGCTCGCAAGTCC	
H3_Q5*.rev	CGGTCTACTTAGTACGAGCCATGGTGGC	
H3_R8*.fwd	CGCTTAGAAGTCCACCGGCGGC	
H3_R8*.rev	ACTTCTAAGCGGTCTGCTTAGTACGAGC	
H3_R17*.fwd	CCCGTAGAAGCAGCTGGCCACCA	
H3_R17*.rev	GCTTCTACGGGGCCTTGCCG	
H3_Q19*.fwd	CAAGTAGCTGGCCACCAAGGC	
H3_Q19*.rev	CCAGCTACTTGCGCGGGGCC	
H3_R26*.fwd	CGCCTAGAAGAGCGCCCCGGC	
H3_R26*.rev	TCTTCTAGGCGGCCTTGGTGG	
H3_R40*.fwd	TCACTAGTACCGTCCCGGCACC	
H3_R40*.rev	GGTACTAGTGAGGCTTCTTCACGCC	
H3_R131*.fwd	CATTTAGGGGGAGAGGGCTGGC	
H3_R131*.rev	CCCCCTAAATGCGGCGGGCG	
H3_R134*.fwd	GGAGTAGGCTGGCCATCATCATCAC	
H3_R134*.rev	CAGCCTACTCCCCACGAATGCG	
D3b_T107*.fwd	GTGAGTAGAAGGACACCAGGACGCG	Cloning of *Dnmt3b** amber mutants
D3b_T107*.rev	TCCTTCTACTCACGGGTGAGCTTTGG	
D3b_K108*.fwd	AGACCTAGGACACCAGGACGCG	
D3b_K108*.rev	GTCCTAGGTCTCACGGGTGAGCTTT	
D3b_H123*.fwd	CCCGATAGAGCAATGGGACCTCCAGC	
D3b_H123*.rev	TTGCTCTATCGGGTTCGGACAGCC	
D3b_K241*.fwd	AGATCTAGGGCTTCTCCTGGTGGC	
D3b_K241*.rev	GCCCTAGATCTTTCCCCACACGAGG	
D3b_P263*.fwd	CCATGTAGGGAATGCGCTGGGTACAG	
D3b_P263*.rev	ATTCCCTACATGGCCTGTCGCTTGG	
D3b_N392*.fwd	ACAACCTAGGACTCTGCTGCTTCTGAGTCCC	
D3b_N392*.rev	AGAGTCCTAGGTTGTGCGTCTTCGACTTTT	
D3b_R490*.fwd	AGGGCTAGGAACTGCTGCTGTGCAGTAACA	
D3b_R490*.rev	AGTTCCTAGCCCTCACAGCACACGG	
D3b_L492*.fwd	CCGTGAATAGCTGCTGTGCAGTAACACAAGCTG	
D3b_L492*.rev	CAGCAGCTATTCACGGCCCTCACAGCAC	

D3b_E562*.fwd	TGACCTGTAGGAATTTGAGCCACCCAAGTTGTACCC	
D3b_E562*.rev	CAAATTCCTACAGGTCAGGATCAGTAGTGAAGAAGTCTTGC	
D3b_L569*.fwd	CCCAAGTAGTACCCAGCAATTCCTGCAG	
D3b_L569*.rev	TGGGTACTACTTGGGTGGCTCAAATTCTTC	
D3b_Y671*.fwd	AGGTTTATAGGAGGGCACAGGAAGGCTCTTCTT	
D3b_Y671*.rev	TGCCCTCCTATAAACCTTTGCGGGCAGGATTG	
D3b_K691*.fwd	CGCCCCTAGGAGGGCGACAACCGTCC	
D3b_K691*.rev	CCCTCCTAGGGGCGGGTATAATTCAGCAAGTG	
Co_1.1_VLHG.fwd	tcgacGTACTCaagCATGGAc	Cloning of *context** amber mutants
Co_1.1_VLHG.rev	aattgTCCATGcttGAGTACg	
Co_3.5_PGLI.fwd	tcgacCCTGGGaagCTCATAc	
Co_3.5_PGLI.rev	aattgTATGAGcttCCCAGGg	
Co_-1.9_FNKQ.fwd	tcgacTTCAATaagAAACAAc	
Co_-1.9_FNKQ.rev	aattgTTGTTTcttATTGAAg	
Co_-2.3_LNKD.fwd	tcgacTTGAATaagAAAGATc	
Co_-2.3_LNKD.rev	aattgATCTTTcttATTCAAg	
Co_-1.4_IKIH.fwd	tcgacATAAAGaagATACATc	
Co_-1.4_IKIH.rev	aattgATGTATcttCTTTATg	
Co_-0.6_LMKE.fwd	tcgacTTAATGaagAAGGAGc	
Co_-0.6_LMKE.rev	aattgCTCCTTcttCATTAAg	
Co_0.3_VRQF.fwd	tcgacGTCAGAaagCAATTCc	
Co_0.3_VRQF.rev	aattgGAATTGcttTCTGACg	
Co_1.1_YLYK.fwd	tcgacTACTTGaagTACAAGc	
Co_1.1_YLYK.rev	aattgCTTGTActtCAAGTAg	
Co_2.0_HLYL.fwd	tcgacCACTTGaagTACCTGc	
Co_2.0_HLYL.rev	aattgCAGGTActtCAAGTGg	
Co_2.9_QGFR.fwd	tcgacCAGGGGaagTTCCGGc	
Co_2.9_QGFR.rev	aattgCCGGAActtCCCCTGg	
Co_3.7_HGLM.fwd	tcgacCATGGAaagCTCATGc	
Co_3.7_HGLM.rev	aattgCATGAGcttTCCATGg	
Co_0.9_HGKD.fwd	tcgacCATGGAaagAAAGATc	
Co_0.9_HGKD.rev	aattgATCTTTcttTCCATGg	
Co_0.8_LNLM.fwd	tcgacTTGAATaagCTCATGc	
Co_0.8_LNLM.rev	aattgCATGAGcttATTCAAg	
Co_-0.8_LGRR.fwd	tcgacTTGGGTaagAGACGTc	
Co_-0.8_LGRR.rev	aattgACGTCTcttACCCAAg	
Co_3.1_LGRR.fwd	tcgacCTTGGAaagCGCAGGc	
Co_3.1_LGRR.rev	aattgCCTGCGcttTCCAAGg	
Co_-0.6_SSAG.fwd	tcgacAGCAGTaagGCAGGTc	
Co_-0.6_SSAG.rev	aattgACCTGCcttACTGCTg	
Co_3.3_SSAG.fwd	tcgacTCCTCCaagGCCGGCc	
Co_3.3_SSAG.rev	aattgGCCGGCcttGGAGGAg	
Co_-0.7_SLRL.fwd	tcgacAGCCTTaagAGACTTc	
Co_-0.7_SLRL.rev	aattgAAGTCTcttAAGGCTg	
Co_2.3_SLRL.fwd	tcgacTCTTTAaagCGCTTGc	

Co_2.3_SLRL.rev	aattgCAAGCGcttTAAAGAg	
Co_0.0_RGLL.fwd	tcgacAGGGGTaagTTACTTc	
Co_0.0_RGLL.rev	aattgAAGTAActtACCCCTg	
Co_3.4_RGLL.fwd	tcgacCGTGGAaagCTCTTGc	
Co_3.4_RGLL.rev	aattgCAAGAGcttTCCACGg	
Co_-0.4_LALA.fwd	tcgacTTGGCTaagTTAGCTc	
Co_-0.4_LALA.rev	aattgAGCTAActtAGCCAAg	
Co_2.6_LALA.fwd	tcgacCTTGCAaagCTCGCGc	
Co_2.6_LALA.rev	aattgCGCGAGcttTGCAAGg	
Co_-0.2_RLPR.fwd	tcgacAGGCTTaagCCACGTc	
Co_-0.2_RLPR.rev	aattgACGTGGcttAAGCCTg	
Co_2.7_RLPR.fwd	tcgacCGTTTAaagCCCAGGc	
Co_2.7_RLPR.rev	aattgCCTGGGcttTAAACGg	
Co_-0.7_LLQL.fwd	tcgacTTGCTTaagCAACTTc	
Co_-0.7_LLQL.rev	aattgAAGTTGcttAAGCAAg	
Co_1.9_LLQL.fwd	tcgacCTTTTAaagCAGTTGc	
Co_1.9_LLQL.rev	aattgCAACTGcttTAAAAGg	
Co_-0.6_LGGR.fwd	tcgacTTGGGTaagGGACGTc	
Co_-0.6_LGGR.rev	aattgACGTCCcttACCCAAg	
Co_2.8_LGGR.fwd	tcgacCTTGGAaagGGCAGGc	
Co_2.8_LGGR.rev	aattgCCTGCCcttTCCAAGg	
Co_0.1_GGVR.fwd	tcgacGGGGGTaagGTACGTc	
Co_0.1_GGVR.rev	aattgACGTACcttACCCCCg	
Co_2.8_GGVR.fwd	tcgacGGTGGAaagGTCAGGc	
Co_2.8_GGVR.rev	aattgCCTGACcttTCCACCg	
Co_-0.9_LLSR.fwd	tcgacTTGCTTaagTCACGTc	
Co_-0.9_LLSR.rev	aattgACGTGActtAAGCAAg	
Co_2.2_LLSR.fwd	tcgacCTTTTAaagTCCAGGc	
Co_2.2_LLSR.rev	aattgCCTGGActtTAAAAGg	
Co_0.6_RGCL.fwd	tcgacAGGGGTaagTGTCTTc	
Co_0.6_RGCL.rev	aattgAAGACActtACCCCTg	
Co_3.0_RGCL.fwd	tcgacCGTGGAaagTGCTTGc	
Co_3.0_RGCL.rev	aattgCAAGCActtTCCACGg	
Co_-0.3_RGIR.fwd	tcgacAGGGGTaagATACGTc	
Co_-0.3_RGIR.rev	aattgACGTATcttACCCCTg	
Co_2.8_RGIR.fwd	tcgacCGTGGAaagATCAGGc	
Co_2.8_RGIR.rev	aattgCCTGATcttTCCACGg	
Co_-1.1_LLTG.fwd	tcgacTTGCTTaagACAGGTc	
Co_-1.1_LLTG.rev	aattgACCTGTcttAAGCAAg	
Co_1.8_LLTG.fwd	tcgacCTTTTAaagACCGGGc	
Co_1.8_LLTG.rev	aattgCCCGGTcttTAAAAGg	
Co_1.1_VLHG*.fwd	tcgacGTACTCtagCATGGAc	
Co_1.1_VLHG*.rev	aattgTCCATGctaGAGTACg	
Co_3.5_PGLI*.fwd	tcgacCCTGGGtagCTCATAc	
Co_3.5_PGLI*.rev	aattgTATGAGctaCCCAGGg	

Co_-1.9_FNKQ*.fwd	tcgacTTCAATtagAAACAAc
Co_-1.9_FNKQ*.rev	aattgTTGTTTctaATTGAAg
Co_-2.3_LNKD*.fwd	tcgacTTGAATtagAAAGATc
Co_-2.3_LNKD*.rev	aattgATCTTTctaATTCAAg
Co_-1.4_IKIH*.fwd	tcgacATAAAGtagATACATc
Co_-1.4_IKIH*.rev	aattgATGTATctaCTTTATg
Co_-0.6_LMKE*.fwd	tcgacTTAATGtagAAGGAGc
Co_-0.6_LMKE*.rev	aattgCTCCTTctaCATTAAg
Co_0.3_VRQF*.fwd	tcgacGTCAGAtagCAATTCc
Co_0.3_VRQF*.rev	aattgGAATTGctaTCTGACg
Co_1.1_YLYK*.fwd	tcgacTACTTGtagTACAAGc
Co_1.1_YLYK*.rev	aattgCTTGTActaCAAGTAg
Co_2.0_HLYL*.fwd	tcgacCACTTGtagTACCTGc
Co_2.0_HLYL*.rev	aattgCAGGTActaCAAGTGg
Co_2.9_QGFR*.fwd	tcgacCAGGGGtagTTCCGGc
Co_2.9_QGFR*.rev	aattgCCGGAActaCCCCTGg
Co_3.7_HGLM*.fwd	tcgacCATGGAtagCTCATGc
Co_3.7_HGLM*.rev	aattgCATGAGctaTCCATGg
Co_0.9_HGKD*.fwd	tcgacCATGGAtagAAAGATc
Co_0.9_HGKD*.rev	aattgATCTTTctaTCCATGg
Co_0.8_LNLM*.fwd	tcgacTTGAATtagCTCATGc
Co_0.8_LNLM*.rev	aattgCATGAGctaATTCAAg
Co_-0.8_LGRR*.fwd	tcgacTTGGGTtagAGACGTc
Co_-0.8_LGRR*.rev	aattgACGTCTctaACCCAAg
Co_3.1_LGRR*.fwd	tcgacCTTGGAtagCGCAGGc
Co_3.1_LGRR*.rev	aattgCCTGCGctaTCCAAGg
Co_-0.6_SSAG*.fwd	tcgacAGCAGTtagGCAGGTc
Co_-0.6_SSAG*.rev	aattgACCTGCctaACTGCTg
Co_3.3_SSAG*.fwd	tcgacTCCTCCtagGCCGGCc
Co_3.3_SSAG*.rev	aattgGCCGGCctaGGAGGAg
Co_-0.7_SLRL*.fwd	tcgacAGCCTTtagAGACTTc
Co_-0.7_SLRL*.rev	aattgAAGTCTctaAAGGCTg
Co_2.3_SLRL*.fwd	tcgacTCTTTAtagCGCTTGc
Co_2.3_SLRL*.rev	aattgCAAGCGctaTAAAGAg
Co_0.0_RGLL*.fwd	tcgacAGGGGTtagTTACTTc
Co_0.0_RGLL*.rev	aattgAAGTAActaACCCCTg
Co_3.4_RGLL*.fwd	tcgacCGTGGAtagCTCTTGc
Co_3.4_RGLL*.rev	aattgCAAGAGctaTCCACGg
Co_-0.4_LALA*.fwd	tcgacTTGGCTtagTTAGCTc
Co_-0.4_LALA*.rev	aattgAGCTAActaAGCCAAg
Co_2.6_LALA*.fwd	tcgacCTTGCAtagCTCGCGc
Co_2.6_LALA*.rev	aattgCGCGAGctaTGCAAGg
Co_-0.2_RLPR*.fwd	tcgacAGGCTTtagCCACGTc
Co_-0.2_RLPR*.rev	aattgACGTGGctaAAGCCTg
Co_2.7_RLPR*.fwd	tcgacCGTTTAtagCCCAGGc

Co_2.7_RLPR*.rev	aattgCCTGGGctaTAAACGg	
Co_-0.7_LLQL*.fwd	tcgacTTGCTTtagCAACTTc	
Co_-0.7_LLQL*.rev	aattgAAGTTGctaAAGCAAg	
Co_1.9_LLQL*.fwd	tcgacCTTTTAtagCAGTTGc	
Co_1.9_LLQL*.rev	aattgCAACTGctaTAAAAGg	
Co_-0.6_LGGR*.fwd	tcgacTTGGGTtagGGACGTc	
Co_-0.6_LGGR*.rev	aattgACGTCCctaACCCAAg	
Co_2.8_LGGR*.fwd	tcgacCTTGGAtagGGCAGGc	
Co_2.8_LGGR*.rev	aattgCCTGCCctaTCCAAGg	
Co_0.1_GGVR*.fwd	tcgacGGGGGTtagGTACGTc	
Co_0.1_GGVR*.rev	aattgACGTACctaACCCCCg	
Co_2.8_GGVR*.fwd	tcgacGGTGGAtagGTCAGGc	
Co_2.8_GGVR*.rev	aattgCCTGACctaTCCACCg	
Co_-0.9_LLSR*.fwd	tcgacTTGCTTtagTCACGTc	
Co_-0.9_LLSR*.rev	aattgACGTGActaAAGCAAg	
Co_2.2_LLSR*.fwd	tcgacCTTTTAtagTCCAGGc	
Co_2.2_LLSR*.rev	aattgCCTGGActaTAAAAGg	
Co_0.6_RGCL*.fwd	tcgacAGGGGTtagTGTCTTc	
Co_0.6_RGCL*.rev	aattgAAGACActaACCCCTg	
Co_3.0_RGCL*.fwd	tcgacCGTGGAtagTGCTTGc	
Co_3.0_RGCL*.rev	aattgCAAGCActaTCCACGg	
Co_-0.3_RGIR*.fwd	tcgacAGGGGTtagATACGTc	
Co_-0.3_RGIR*.rev	aattgACGTATctaACCCCTg	
Co_2.8_RGIR*.fwd	tcgacCGTGGAtagATCAGGc	
Co_2.8_RGIR*.rev	aattgCCTGATctaTCCACGg	
Co_-1.1_LLTG*.fwd	tcgacTTGCTTtagACAGGTc	
Co_-1.1_LLTG*.rev	aattgACCTGTctaAAGCAAg	
Co_1.8_LLTG*.fwd	tcgacCTTTTAtagACCGGGc	
Co_1.8_LLTG*.rev	aattgCCCGGTctaTAAAAGg	

REFERENCES

1. Elsässer,S.J., Ernst,R.J., Walker,O.S. and Chin,J.W. (2016) Genetic code expansion in stable cell lines enables encoded chromatin modification. *Nat. Methods*, **13**, 158–164.

2. Cigler,M., Müller,T.G., Horn-Ghetko,D., von Wrisberg,M.-K., Fottner,M., Goody,R.S., Itzen,A., Müller,M.P. and Lang,K. (2017) Proximity-Triggered Covalent Stabilization of Low-Affinity Protein Complexes In Vitro and In Vivo. *Angew. Chem. Int. Ed Engl.*, **56**, 15737–15741.

3. Mayer,S.V., Murnauer,A., von Wrisberg,M.-K., Jokisch,M.-L. and Lang,K. (2019) Photo-induced and Rapid Labeling of Tetrazine-Bearing Proteins via Cyclopropenone-Caged Bicyclononynes. *Angew. Chem. Int. Ed Engl.*, **58**, 15876–15882.

4. Mideksa,Y.G., Fottner,M., Braus,S., Weiß,C.A.M., Nguyen,T.-A., Meier,S., Lang,K. and Feige,M.J. (2020) Site-Specific Protein Labeling with Fluorophores as a Tool To Monitor Protein Turnover. *Chembiochem*, 10.1002/cbic.201900651.

5. Nikić,I., Estrada Girona,G., Kang,J.H., Paci,G., Mikhaleva,S., Koehler,C., Shymanska,N.V., Ventura Santos,C., Spitz,D. and Lemke,E.A. (2016) Debugging Eukaryotic Genetic Code Expansion for Site-Specific Click-PAINT Super-Resolution Microscopy. *Angew. Chem. Int. Ed Engl.*, **55**, 16172–16176.

6. Binder,A., Lambert,J., Morbitzer,R., Popp,C., Ott,T., Lahaye,T. and Parniske,M. (2014) A modular plasmid assembly kit for multigene expression, gene silencing and silencing rescue in plants. *PLoS One*, **9**, e88218.

7. Bindels,D.S., Haarbosch,L., van Weeren,L., Postma,M., Wiese,K.E., Mastop,M., Aumonier,S., Gotthard,G., Royant,A., Hink,M.A., *et al.* (2017) mScarlet: a bright monomeric red fluorescent protein for cellular imaging. *Nat. Methods*, **14**, 53–56.

8. Shaner,N.C., Lambert,G.G., Chammas,A., Ni,Y., Cranfill,P.J., Baird,M.A., Sell,B.R., Allen,J.R., Day,R.N., Israelsson,M., *et al.* (2013) A bright monomeric green fluorescent protein derived from Branchiostoma lanceolatum. *Nat. Methods*, **10**, 407–409.

9. Lo,C.-A., Kays,I., Emran,F., Lin,T.-J., Cvetkovska,V. and Chen,B.E. (2015) Quantification of Protein Levels in Single Living Cells. *Cell Rep.*, **13**, 2634–2644.

10. Mulholland,C.B., Smets,M., Schmidtmann,E., Leidescher,S., Markaki,Y., Hofweber,M., Qin,W., Manzo,M., Kremmer,E., Thanisch,K., *et al.* (2015) A modular open platform for systematic functional studies under physiological conditions. *Nucleic Acids Res.*, **43**, e112.

11. Chou,C., Uprety,R., Davis,L., Chin,J.W. and Deiters,A. (2011) Genetically encoding an aliphatic diazirine for protein photocrosslinking. *Chem. Sci.*, **2**, 480–483.

12. Lang,K., Davis,L., Torres-Kolbus,J., Chou,C., Deiters,A. and Chin,J.W. (2012) Genetically encoded norbornene directs site-specific cellular protein labelling via a rapid bioorthogonal reaction. *Nat. Chem.*, **4**, 298–304.

13. Hermann,M., Stillhard,P., Wildner,H., Seruggia,D., Kapp,V., Sánchez-Iranzo,H., Mercader,N., Montoliu,L., Zeilhofer,H.U. and Pelczar,P. (2014) Binary recombinase systems for high-resolution conditional mutagenesis. *Nucleic Acids Res.*, **42**, 3894–3907.

14. Ran,F.A., Hsu,P.D., Wright,J., Agarwala,V., Scott,D.A. and Zhang,F. (2013) Genome engineering using the CRISPR-Cas9 system. *Nat. Protoc.*, **8**, 2281–2308.
